# Evolution of ribosomal protein network architectures

**DOI:** 10.1038/s41598-020-80194-4

**Published:** 2021-01-12

**Authors:** Youri Timsit, Grégoire Sergeant-Perthuis, Daniel Bennequin

**Affiliations:** 1 Mediterranean Institute of Oceanography UM 110, Aix-Marseille Université, CNRS, IRD, Campus de Luminy, Marseille, France; 2 Institut de Mathématiques de Jussieu - Paris Rive Gauche (IMJ-PRG), UMR 7586, CNRS, Université Paris Diderot, Paris, France

**Keywords:** Systems biology, Biochemical networks

## Abstract

To perform an accurate protein synthesis, ribosomes accomplish complex tasks involving the long-range communication between its functional centres such as the peptidyl transfer centre, the tRNA bindings sites and the peptide exit tunnel. How information is transmitted between these sites remains one of the major challenges in current ribosome research. Many experimental studies have revealed that some r-proteins play essential roles in remote communication and the possible involvement of r-protein networks in these processes have been recently proposed. Our phylogenetic, structural and mathematical study reveals that of the three kingdom’s r-protein networks converged towards non-random graphs where r-proteins collectively coevolved to optimize interconnection between functional centres. The massive acquisition of conserved aromatic residues at the interfaces and along the extensions of the newly connected eukaryotic r-proteins also highlights that a strong selective pressure acts on their sequences probably for the formation of new allosteric pathways in the network.

## Introduction

Ribosome structures^1–4^ have evolved by the accretion of rRNA and ribosomal (r)-proteins around a universal core considered as a relic of ancient translation systems that co-evolved with the genetic code^5–9^. They followed distinct evolutionary pathways to form the bacterial, archaeal and eukaryotic ribosomes^10–14^ whose overall structures are well conserved within kingdoms^15–26^. A concomitant increase in the complexity of ribosome assembly processes, ribosome structures, efficiency and fidelity of protein production is observed from prokaryotes to eukaryotes^27–29^. While the past decade studies have provided a detailed mechanistic understanding of almost all of the translation steps, one of the major challenges in ribosome research is how information is transmitted and processed between remote functional sites such as the tRNA binding sites, the peptidyl-transfer centre (PTC) and the peptide exit tunnel, during protein synthesis.

Growing experimental evidences have shown that distant ribosomal functional sites not only continuously “sense” incoming molecular signals but also “transmit” them to each other. For example, long-range signalling between the decoding centre monitors the correct geometry of the codon-anticodon and other distant sites such as the Sarcin Ricin Loop (SRL) or the E-tRNA site^30^. The r-protein uL3 also plays a key role in the allosteric coordination of the peptidyl transferase centre (PTC)^31^ and the A-site^32^. Similarly, the r-proteins that sense the nascent peptide within the exit tunnel participate in the regulation of co-translational folding and communicate with remote functional sites such as the PTC^33,34^. Communication processes also coordinate the complex ribosomal movements during translation, such as the ratchet-like motion between the two subunits^35–37^.

In addition to their roles in rRNA folding and ribosome assembly^38–43^, extensions systematically form complex r-protein networks through tiny interactions, in the three kingdom ribosomes^2,3,44^. Unlike most protein networks that occur transiently in the cells^45^, r-protein networks are particular in that they are woven by tiny interactions in mature ribosomes, once the stages of their biogenesis is complete^28^. It has been suggested that they could contribute to information transfer and processing during the course of protein synthesis^44,46^.

Understanding how r-protein networks have evolved is an indispensable step to get further insights about their biological significance. Several questions remain to be answered: are the tiny interfaces structurally and phylogenetically conserved in the three domains of life? How has r-protein network connectivity evolved over time and does graph theory provide information about their evolution and functionality? Here, we present a global study of the evolution of r-protein networks, through a phylogenetic, structural and mathematical analysis of their architectures.

## Results

### r-Protein network conservation and expansion during evolution

Beyond their fascinating diversity, the interactions between ribosomal proteins (PPi) constitute variations around a common theme: how to maintain the tiniest interfaces between long filamentous extensions (ext) or globular domains (G) of a wide variety of r-protein structures (Supplementary Fig. 1). All of these interactions, which may also contact functional sites (mRNA, tRNAs, PTC or peptide exit tunnel), form complex interactomes that have been analysed from an evolutionary and a functional perspective.

**Figure 1:**
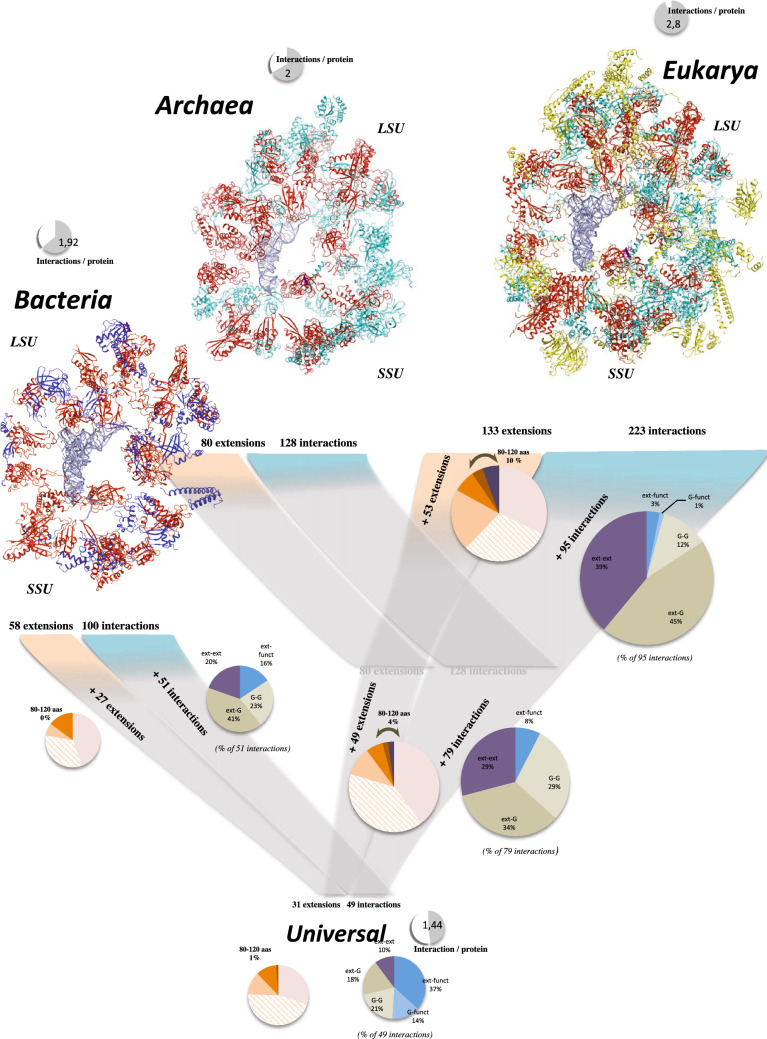
Evolution of the r-protein interactomes. In the three kingdoms’ networks, the r-protein components are represented according to their evolutionary status with the following colour code: red: universal; blue: bacteria; cyan: archaea; yellow: eukarya. Pie charts report the proportion of extension sizes and of contact types that have been acquired at each transition, respectively. Abbreviations: ext-ext: extension-extension; ext-G: extension-globular domain; ext-funct: extension-functional site; G-G: globular domain-globular domain; G-funct: globular domain-functional site.

Comparing the three kingdoms’ networks makes it possible to identify the evolutionary status of all their components (Supplementary Tables 1 and 2) and to trace how they have developed over time. Network archaeology has first revealed the existence of a universal (ABE) network, that consists of 49 strictly conserved connections probably present before the radiation of the bacteria and archaea^12^ (Supplementary Fig. 2). From this common core, the Bacterial (B), Archaeal (A) and Eukaryotic (E) networks have gradually developed through the addition of new proteins and/or new connections. Each kingdom ribosome displays a network with distinct but well-conserved architecture (Supplementary Fig. 3). Although tiny, PPi interfaces are both structurally and phylogenetically well conserved within each kingdom (Supplementary Fig. 4–8). Noticeably, conservation mapping shows that interfaces are significantly better conserved in eukaryotes, especially in the small subunit.

**Figure 2:**
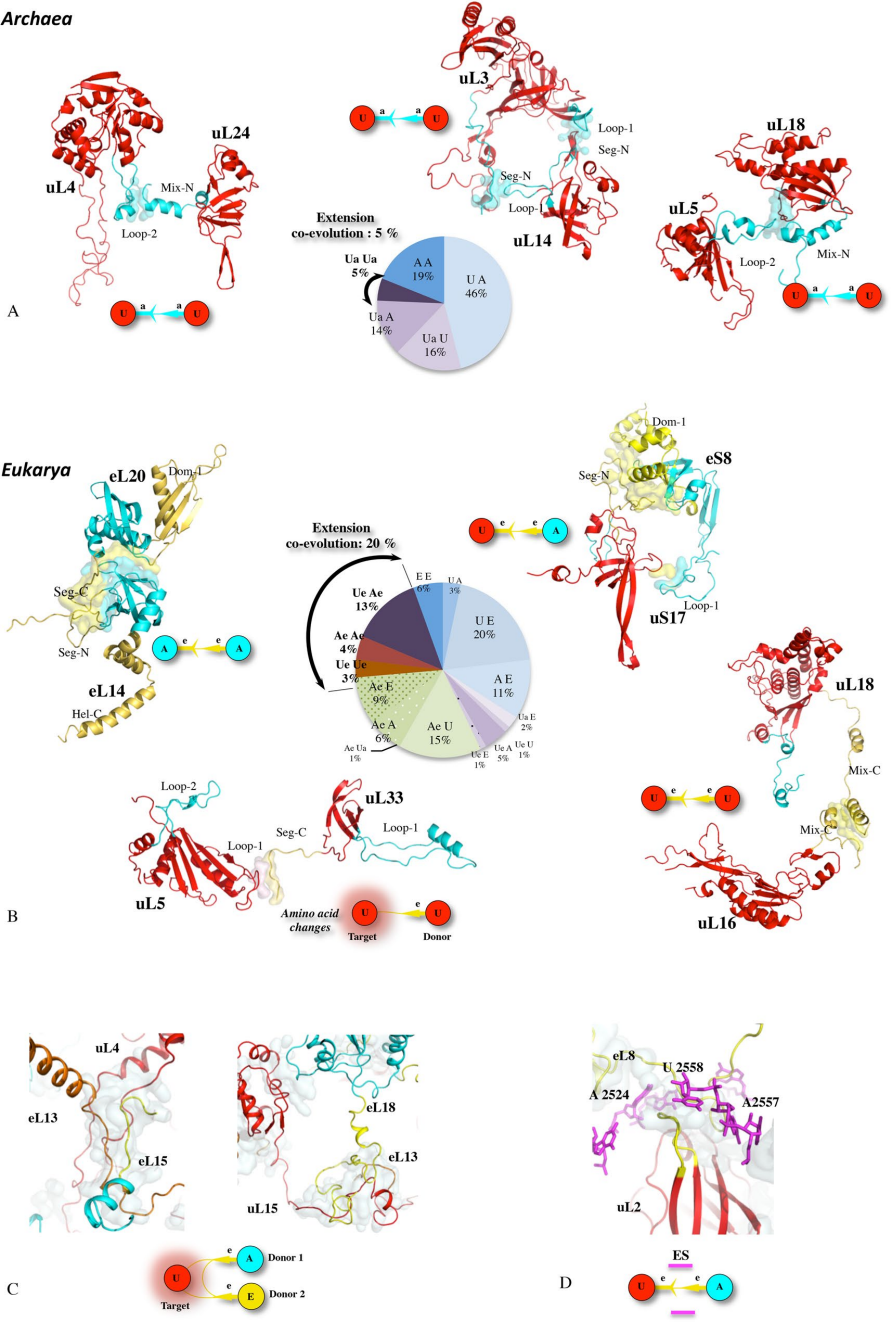

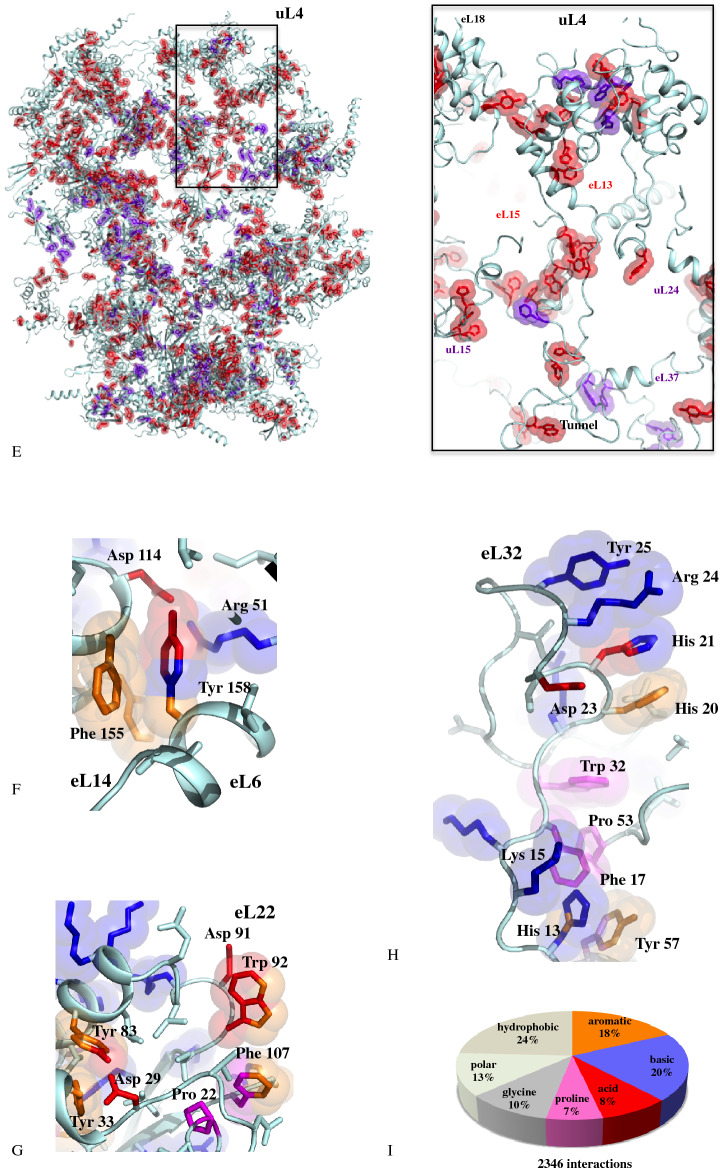
Coevolutions for networking. Extension coevolutions in archaea (**A**) and eukarya (**B**). The extensions and r-proteins are coloured according to their evolutionary status. Pie charts report the proportions of the connections specifically acquired in the A and E networks according to their evolutionary status. (**C**) Multiple extension coevolutions toward a common “ancient” target. (**D**) Coevolution of a eukaryotic PPi interface (uL2–eL8) with unpaired bases of ES. Abbreviations: U, A, B and E correspond to universal, archaeal, bacterial and eukaryotic r-proteins. Ub: Universal protein with an extension acquired in (**B**) bacteria. Ua: Universal protein with an extension acquired in (**A**) archaea. Ue: Universal protein with an extension acquired in (**E**) eukarya. Ae: Archaeal protein with an extension acquired in (**E**) eukarya. (**E**) Massive acquisition of new conserved aromatic residues in the eukaryotic ribosome. New conserved and “ancient” (already conserved in archaea) aromatic residues are represented with red and purple blue spheres, respectively. The rectangle represents a close-up around the uL4 r-protein. (**F**) Interactions between conserved aromatic residues and other conserved amino acids at the interface eL6–eL14. (**G**) Intra molecular anion-π and proline-π interactions between conserved aromatic residues in the globular domain of r-protein eL22. (**H**) Cluster of conserved aromatic residues in the extension of eL32 and their interactions with various amino acids. From F to H, the atoms of the aromatic residues (orange) are coloured in function of their interacting partners: blue: basic (lys, arg); red: acidic (glu, asp) and magenta: proline. (**I**) Proportions of conserved aromatic residues involved in intra or intermolecular interactions with other types of conserved amino acids.

**Figure 3:**
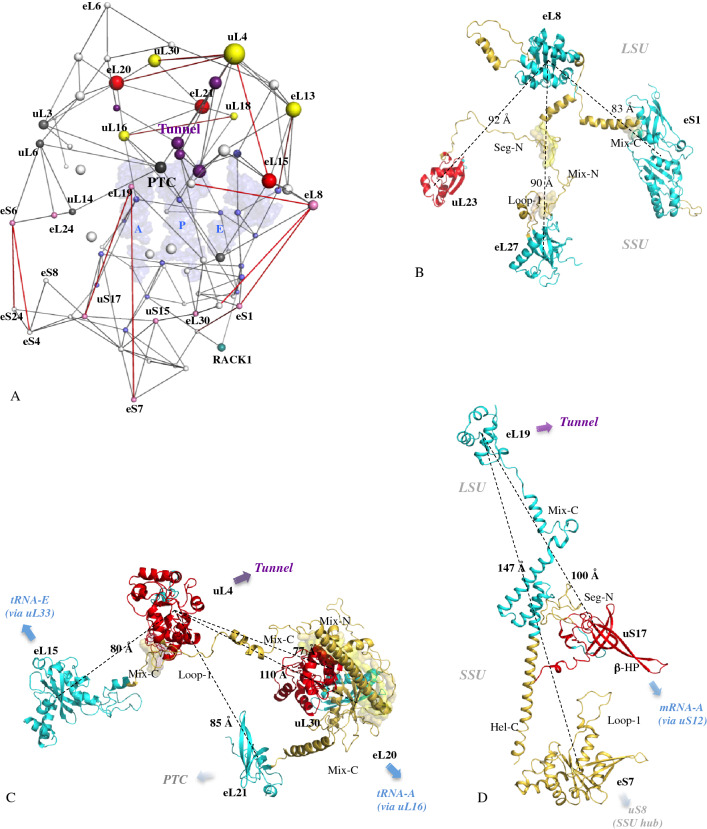
Extensions coevolve to connect remote functional modules. (**A**) Graph of the eukaryotic network with the largest distances between interconnected centres of mass are represented in red. The sphere radius are proportional to the values of the eigenvector centrality and are coloured according to their functional status (Supplementary Fig. 11): light blue: mRNA or tRNA functional modules; gray: PTC functional module; violet: tunnel functional module; light pink: subunit brides; yellow: node that bridges two distinct functional modules; red: node that bridges three distinct functional modules. The three tRNAs are represented by pale blue transparent surfaces. (**B**-**D**) Interconnections of the most distant r-proteins by co-evolution of their extensions. R-protein components are represented according to their evolutionary status.

**Figure 4:**
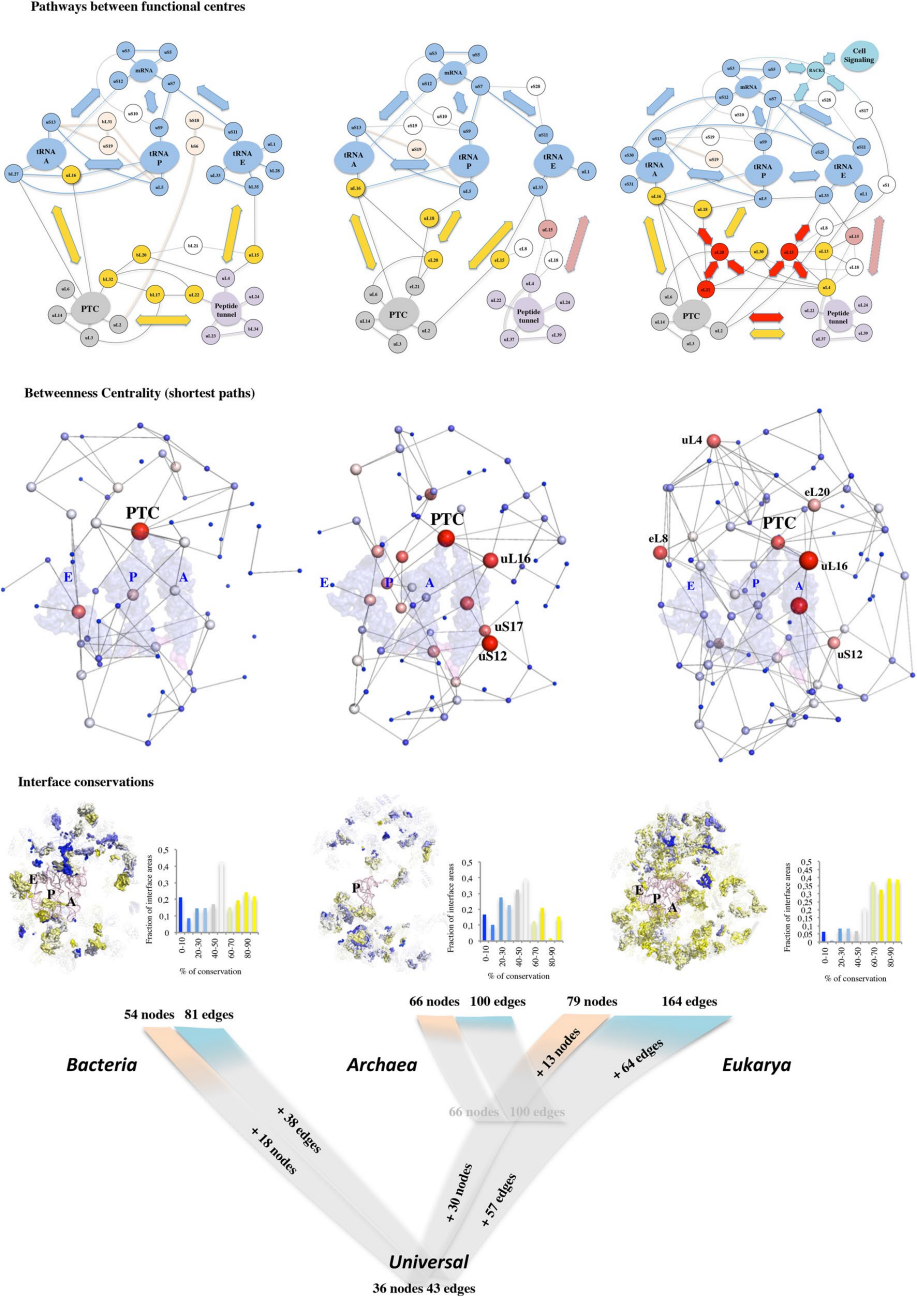
Congruence of evolutionary traits in network expansions. The three kingdoms’ networks are represented according to (from top to bottom) their functional, mathematical and phylogenetic properties. The colour codes corresponding to the functional status of the nodes is the same as in Fig. 3. The graphs are represented by connected spheres whose radius and colour gradient (white > red) are proportional to the betweenness centrality values. Largest red sphere correspond to maxima of betweenness centrality. The three tRNAs A, P and E and the mRNA are represented by transparent surfaces. The mapping of the interfaces and the distribution of the conserved surfaces are represented by a colour gradient from blue (0 conservation) to yellow (100% conservation).

Except some minor variations, the interactomes of each kingdom are very well conserved even in distant clades. In some bacterial species, the acquisitions of new extensions are generally associated with the formation of new inter-protein contacts. For example, the “new” C-mix extension of *M. smegmatis* bS16 interacts with uS4^24^ and the *E. coli* uS14 internal loop (15–54; *E. coli* numbering) interacts with uS19^21,22^ (Supplementary Fig. 9). In these two cases, rRNA structures of the different specie’s ribosomes are conserved and cannot simply justify changes in protein network. In another hand, variations in network connectivity may be also associated with changes in rRNA structures. In the *M. smegmatis* ribosome, the path of bL17 extension differs from that of the other bacterial species: instead of contacting uL3, the bL17 N-seg returns to its own globular domain and stabilizes a specie’s specific extra-helical adenine.

The architecture of the eukaryotic network is also well conserved in the three distant clades *opistokhonts*, *chromoalveota* and *excavata* analysed (Supplementary Fig. 10A,B). A particularly interesting feature of *plasmodium* ribosome network^19^ is the direct connection between uL4 with uL16 (Supplementary Fig. 10C). In the human ribosome^20^, eL6 has a different trajectory that complicates the interaction network compared to yeast (Supplementary Fig. 10D). In the *leishmania* ribosome, the new extensions that have been also reported for the r-proteins uL13 and eL33 are thought to stabilize the fragmented rRNAs specific to this particular clade^23^ (Supplementary Figs. 10E,F). Thus, while the networks are very well preserved even in very distant taxa within each kingdom^12,47^, they also display minor variations in some species where the new connections may reflect particular adaptations of the translation systems.

Another way of understanding the networks is their functional organization. As a first approximation, r-proteins can be distinguished according to the contacts they form with the different categories of functional centres (Supplementary Fig. 11). While some proteins cluster in modules around the main functional centers of the ribosome: mRNAs, tRNAs, PTCs and the peptide tunnel, others build bridges between these modules or between ribosome subunits. Mainly developed in the small subunit (SSU), the ABE network contains a high proportion of connections between r-proteins and functional sites. On the contrary, bridges develop in the later phases of ribosome evolution. Different categories of bridges can be distinguished according to the number and type of functional sites they connect. For example, proteins that bridge two different functional sites are common in bacteria and archaea. In contrast, proteins such as eL15, eL20 and eL21 that link together three functional sites are only found in eukaryotes (Supplementary Figs. 11D-E).

### Molecular mechanisms of network expansions

How new incoming r-proteins or newly acquired extensions contribute to network expansions? Systematic comparison of the networks made it possible to identify, one by one, the events that marked the expansion of the networks at each evolutionary transition. The networks are progressively woven by a combination of interactions between protein components (G or ext) of different evolutionary statuses (ABE, B, A or E) (Fig. 1). The new interactions either reinforce existing links between r-proteins, or connect nodes that were not previously connected. Each subunit starts its evolutionary history from different network architectures: an already densely connected SSU and a poorly connected LSU in the ABE network. Whereas the number of connections of the two subunits evolved roughly symmetrically in archaea, bacteria and eukarya underwent a spectacular development of the LSU connectivity (Supplementary Fig. 12 and Supplementary Table 3). Network expansion is also characterized by an increase in multiple connections between protein pairs (Supplementary Table 4). Noticeably, the r-proteins that display the highest number of multiple connections correspond to mRNA binders (uS3, uS5).

Early (ABE- > B, ABE- > A) and late (A- > E) network expansions display specific molecular evolutionary features (Fig. 1; Supplementary Fig. 12). Whereas a massive incorporation of new interacting r-proteins characterizes the early evolutionary stages, it becomes a minority in the A- > E transition where ancient r-proteins become more connected to each other. During the ABE- > B transition, most of the new interactions of the bacterial network (58% U-B interactions) occur between the universal proteins (U) (G or ext) and the newly acquired bacterial r-proteins (B). Interactions between incoming bacterial proteins (B-B) only represent 19% of the interactions and a minority of contacts involve bacterial acquired extensions of universal proteins (Ub) with universal r-protein (12% Ub-U) or with bacterial proteins (9% Ub-B). Thus, in the bacterial ribosome, extensions acquired in bacteria (Ub) only play a minor role in networking. During the ABE- > A transition, the majority of new contacts are also formed between the universal proteins (U) and the newly acquired archaeal proteins (A) (47% U-A interactions). Similar to bacteria, 19% of the interactions are observed between the newly acquired proteins (A-A). However, a greater contribution (35%) of the archaeal specific extensions (Ua) contributes to the formation of the A network. The A- > E transition displays new evolutionary traits. Although it is the most connected, the eukaryotic network incorporates only 11 new proteins, the smallest number of new incoming r-proteins and most of the new contacts (57%) are mediated by the newly acquired extensions in universal and archael r-proteins (Ue and Ae). The A- > E transition therefore displays a sharp increase of connections between ancient and previously unconnected r-proteins (universal or archaeal) that have acquired new extensions.

As a result, each evolutionary transition is distinguishable by different proportions of contact types. For example, while virtually all interactions between r-proteins and functional sites have already been established in the universal network (45% of the total interactions), the connections to functional centres sharply decrease from 14% in B, to 4% and 2% in A and E networks, respectively. Another remarkable evolutionary feature is ever-increasing number of contact involving extensions during evolution. For example, the eukaryotic networks have the lowest proportion of G-G interactions and the highest G-ext (45%) and ext-ext (39%) interactions. Among them, the majority of new contacts in E involve C-terminal extensions. The eukaryotic LSU network evolution is unique in that it displays the highest number of extension-extension interactions.

Thus, networking gradually depends on the emergence of new extensions (Supplementary Fig. 13) and a majority of r-proteins develop extensions that are systematically involved in network interactions (Supplementary Table 5). A majority of r-proteins (uL3, uL4, uL18, uL22, uL23 and uL33) that have acquired new extensions at all evolutionary stages belongs to the LSU. The categories of newly acquired extensions also vary according to evolving transitions (Supplementary Fig. 14). While acquiring internal extensions (loops and β-hairpins) is an early evolutionary trait, a strong bias towards C-terminal extensions is observed during the A- > E transition. The increase of the proportion of “mix” shows that the unstructured segments (seg) acquire gradually more β-helical regions. A net increase of extension sizes is also observed during evolution (Supplementary Fig. 14). The percentage of extensions greater than 80 amino acids increases from 1 to 10% from the ABE to E networks. The longer extensions interconnect more distant r-proteins in the networks: the distances between the interconnected proteins gradually increase in course of the evolution (Supplementary Fig. 15). Extensions can therefore be considered as an evolutionary solution for inter-connecting remote network nodes while preserving the ribosome or protein overall structures and sizes.

Unlike the growing roles of extensions in protein networks, extension-rRNA interactions only slightly increase from prokaryotes to eukaryotes (Supplementary Fig. 16A,B) and are very moderate compared to the jump in inter-protein connectivity. Moreover, the eukaryotic ribosome reveals that eukaryotic specific extensions rather interact with other r-proteins than with eukaryotic specific RNA expansion segments (Supplementary Fig. 16C). Also, extensions acquired in eukaryotes contribute little to RNA interactions (22–26%, for SSU and LSU, respectively) relatively to their archaeal or universal counterparts (78%). This demonstrates that the acquisition of new extensions during evolution are mainly linked to their roles in protein–protein interactions.

#### Co-evolution for networking

The expansion of networks also relies on a curious phenomenon of coordinated acquisition of extensions or r-proteins that establish new contacts (extension-protein: Ub-B, Ua-A, Ue-E; extension-extension: Ua-Ua, Ue-Ue, Ae-Ae) (Fig. 2; Supplementary Table 3). At each evolutionary transition, extensions have been concurrently acquired, or coevolved^48^ for connecting remote r-proteins. In the bacterial ribosome, the bacterial extensions acquired by the universal r-proteins (uL5-mix-N, uL13-seg-N, uL23-β-HP) coincide with the incorporation of new bacterial proteins (bL31, bL20-bL21, bL34, respectively) to which they are connected. In the ABE- > A transition, several coordinated acquisitions of new extensions such as uL4-loop-2 and uL24-mix-N that interact together are observed (Fig. 2A). This phenomenon is significantly amplified in the A- > E transition where many new E specific extensions have been acquired in both universal and archaeal r-proteins to inter-connect them (Fig. 2B). Coevolutions between multiple r-proteins or rRNA partners have been detected in eukaryotic ribosomes. Tripartite assembly of co-occurred extensions on a third target r-protein (eL13-eL15 > uL4 and of eL13-eL18 > uL15) or with rRNA ES have been observed (Fig. 2C). A remarkable structural motif is formed by the interaction of the extensions of uL2 and eL8 whose interface is literally caged inside a sphere formed by unpaired bases of ES (Fig. 2D). Most of the coevolved extensions display highly conserved residues along themselves and in the interfaces they form with their partners (Supplementary Table 6).

Co-evolution is also observed at the amino acid level (Fig. 2E). The A > E transition provides the unique opportunity to follow the evolution of r-proteins common to both kingdoms, at the amino acid level, according to their change in connectivity in the network. We have tracked changes in all target proteins that receive new contacts in eukaryotes while remaining in a similar rRNA context (Supplementary Table 3). This reveals that the formation of new contacts systematically correlate with the apparition of new conserved residues, mainly aromatic ones, not only at the new interfaces but also regularly spread along the r-proteins (Supplementary Fig. 17). As a result, a massive acquisition of new conserved aromatic residues parallels the spectacular jump in connectivity of the eukaryotic network and likely accounts for the higher conservation rate of the eukaryotic r-protein and PPi (Fig. 2E; Supplementary Table 7). A strong selective pressure is therefore exerted on the sequence of the newly interconnected proteins. Firstly, conserved amino acids that were associated with contacts already present in archaea are not only still conserved, but very often change from "similar" (conservation by amino acid type: aromatic, basic, acidic, polar or hydrophobic) to strictly conserved. Second, the new aromatic amino acids most often form clusters and structural motifs where they participate in combined π–π, cation-π, anion-π or proline-π interactions^49–52^ (Fig. 2F-I). Thirdly, and surprisingly, the newly conserved amino acids most often appear, not only at the new interfaces, but also evenly distributed along the newly connected proteins (Fig. 2E-I). A similar correlation between the appearance of new aromatic residues and the formation of new contacts has been also observed in intra-kingdom evolutions as for example in uS4 of *M. smegmatis* (Supplementary Fig. 9B) or uS19 of *E. coli* (Supplementary Fig. 9F). It is therefore likely that these new conserved amino acids have been selected to play a role both in the formation of interfaces but also in the constitution of new allosteric pathways. The complexity and particular electrostatic properties of these conserved structural motifs merit further study and reinforces the previous hypotheses that they may play a particular role in the transfer of information between the proteins in the network^44,46^.

A special case is the “distant” approach of the extensions of uL4 and uL15, without forming direct contact in both archaea and eukaryotes (Supplementary Fig. 18). Yet, despite the fact that there is no direct contact between them, aromatic residues are retained, at the level of their closer approach (10 Å), on both partners. But although the bacteria have developed a longer extension on uL15 that establishes true contact with uL4, they have also retained this structural motif and the aromatic residues (Supplementary Fig. 18). This suggests that even aromatic residues that are not in direct contact could participate to allosteric communication. Thus, this structural motif could help to establish an important functional interconnection between the tRNA-E and tunnel modules (Supplementary Fig. 11).

### Graph theory highlights node functionalities

Graph theory^45^ has been used to further characterize the evolution of r-protein networks. Previous studies in which rRNA was considered as nucleotide network revealed interesting correlations between graph properties and ribosome functionality^53^. Here, the r-protein networks have been modelled as undirected graphs where the nodes correspond to the centres of mass of the r-protein globular domains or the functional centres and the edges represent their interactions (a single connection between a protein pair) (Fig. 3A; Supplementary Fig. 19). In the three kingdom’s networks, the node inter-connectivity and their centrality values are significantly distinct from that of random graphs (Supplementary Table 8). Secondly, interesting correspondences between the graph theory and the ribosome biology have been found. For example, betweenness centrality (BC) that defines the number of shortest paths passing through a node is a key determinant of network functionality in that it measures the extent to which a node influences the information spread on the network. Remarkably, without knowing ribosome biology, mathematics indicates that in the three kingdom’s networks, the BC maxima correspond to the PTC, which catalyses the peptidyl-transfer reaction during protein synthesis (Fig. 4; Supplementary Fig. 19). This reveals that r-protein network architectures have evolved convergently, so that the PTC becomes a major player in information spread, in perfect agreement with its central role in ribosome catalysis^31^ and allostery^53,54^. In eukaryotic ribosomes, network control is also distributed to other actors including uL16, tRNA-A, eL8 and uL4. Interestingly, uL16 has been also highlighted by biochemical studies as a key player in information transmission of the eukaryotic ribosome^36^ and disease^55^. In a similar way, the eigenvector centrality (EV) maxima that illustrate the importance of a node (by its property of being itself connected to important nodes) correspond to the key proteins that establish bridges between functional modules in eukaryotes (Fig. 3A; Fig. 4; Supplementary Fig. 19A).

Tracking the fate of the network nodes during the evolutionary transitions also highlights that those undergoing the most important jumps in centrality values, correspond to the proteins that play a critical role in bridging functional modules (Supplementary Fig. 19B,C). Furthermore, the distances between these particular nodes are among the largest in the network and are the result of the co-evolution of their extensions (Fig. 3). This observation therefore provides a functional meaning to the increase in the size of the extensions and the progressive increase in the distances of the interconnected r-proteins over the course of evolution (Supplementary Table 9): they gradually interconnect distant functional centres. The intra- and inter-connectivity of the functional modules grows indeed steadily over the course of evolution (Fig. 4). Within functional clusters, some r-proteins such as uL4 or uL16 have a special fate in that they are selected by the evolution to establish a bridge with other clusters. But another phenomenon consists in the appearance of new bridges between functional modules by proteins that develop proteins between them. Eukaryotes are the only ones to have proteins such as eL15, eL20, eL21 that bridge three distinct categories of functional sites such as PTC, the tunnel and a tRNA binding site (Fig. 4).

Overall, graph theory shows that the interconnectivity of the network is not the result of chance, but rather the result of selective pressure that has led to a functional optimization of its architecture for bridging and spreading information between functional modules. Thus, to better understand the evolution of r-proteins, it is necessary to take into account both the increase in their connectivity but also their changing mathematical and functional status in the network.

### Discussion

The role of r-proteins and their extensions has long been enigmatic. It has been suggested that due to their charged and dynamic nature, extensions could contribute to ribosome assembly in facilitating the rRNA folding^38–43^. Mutagenic studies have shown, for example, that the N-terminal extensions of uS12, eL8, uL29 and uL30 play a role in the assembly of the bacterial SSU^56^ or the eukaryotic LSU^57^. However, other studies demonstrated that extensions participate in both translation and ribosomal assembly^58–61^ and many studies from Dinman’s group have revealed that they play essential roles in long-range communication between functional sites^32,62–64^. Several studies have also highlighted the role of r-protein communication for efficient translation and its link to yeast signalling pathways^65^. The dramatic expansion of r-protein networks during evolution fits well with these studies and opens new perspectives towards more complex extension functions than rRNA assembly or stabilization.

Our study reveals that network expansion is produced by the collective evolution of r-proteins. The asymmetrical evolution of the two subunits, the heterogeneous landscape of the r-protein evolutionary history and graph theory show that the evolution of network connectivity did not occur at random. On the contrary, the concerted acquisition of new extensions and connections gradually relates the functional modules, progressively differentiate the functional status of the nodes and places the functional centres in central positions of the network. Moreover, in the course of evolution, certain connections involving key r-proteins belonging to functional modules are also strengthened and give rise to multiple binding. The strong selective pressure that is also expressed at the amino acid establishes an interesting link between the network architectures and the r-protein phylogeny (Supplementary Table 7) and suggests that the networks have gradually evolved sophisticated allosteric pathways within and between r-proteins. The congruence between independent evolutionary traits indicates that the network architectures evolved to relate and optimize the information spread between functional modules.

Thus, r-proteins may have evolved towards multiple functions. While they likely retained their primary functions devoted to the assembly and stabilization of the rRNA, they may have acquired new functions related to the increasing complexity of ribosome tasks in the course of evolution. Previous studies have revealed that rRNA can also participate in remote communication between functional sites^53^. rRNA and r-protein networks may have also co-evolved for optimizing not only assembly processes but also r-protein synthesis^46^. The expansion of network connectivity indeed follows the increasing accuracy and complexity of the ribosome’s tasks from prokaryotes to eukaryotes^27,29^. The burst of LSU connectivity parallels its gradual specialization in regulating multiple sub-functions that co-emerged with the growth of cellular complexity.

Our study predicts that any changes in the r-proteins that may affect network connectivity should modify the translation efficiency and accuracy and provides new hints to understand both ribosome heterogeneity that plays a key role in translation regulation^66^ and diseases associated with r-protein mutations^55^. In addition, the network shortcuts in parasitic or virulent species such as the uL4-uL16 interaction that connect directly the tRNA-A to the tunnel modules in *plasmodium* or the new bS16-uS4 connection in *mycobacterium* opens a conceptual framework for therapeutic perspectives. Thus, networking can circumvent the physical constraints associated with growth and the addition of new constituents in an evolving system. Our study suggests that accretion and networking probably co-existed since the earliest life forms.

## Methods

### Selection of ribosome structures in the PDB

The highest resolution X-ray and cryo-EM ribosome structures representative of the three kingdoms (B: Bacteria; A: Archaea; E: Eukarya) have been selected from the Protein Data Bank (PDB)^67^ and used to analyse the r-protein networks and the interactions between the r-protein components: the globular domains (G), the extensions (Ext) or the functional sites (PTC, tunnel, mRNA, tRNAs)^16–21^ (Supplementary Table 10). The ribosomes in the three kingdoms of life were structurally aligned using the program *pymol*^68^. The r-proteins have been named according the new nomenclature^69^.

### Analysis of r-protein extension evolution

Protein extensions are distinct parts of the protein structure that protrude from globular domains^13,14,43,44^. They have been divided into five categories according to their structural properties: segments, mix (segments and α-helices), α-helices, loops, and β-hairpins. Internal extensions such as loops or β-hairpins result from insertions within the peptidic chain. External extensions have been added to the N- or C-terminal ends. Equivalent proteins may also differ by extra-domains (dom) across kingdoms. Insertion and deletions (Indels) events in proteins are often used as taxon specific markers^70^. The comparison of the superimposed r-proteins of A, B and E ribosomes has made it possible to follow the evolutionary history of their extensions and domains. The gradual acquisition of extensions during the major evolutionary transitions (ABE—> A, ABE—> B and A—> E) has been then inferred. As previously observed^41^, this procedure indicated that bL33 and eL42 that co-locate close to the E-site have the same globular domain but developed different extensions in A, B and E. They were renamed “universal protein uL33”. The fact that bL33 belongs to a set of bacterial specific genes located in the clusters containing the universal protein genes^71^ supports this hypothesis.

The r-proteins, their extensions and their inter-connections have been compared in the three kingdom’s ribosomes and have been coloured according to their evolutionary status (Supplementary Table 2). The same colour code is used throughout the whole manuscript: **red**: Universal (ABE); **blue**: Bacteria (B); **cyan**: common between Archaea and Eukarya (A); **yellow**: Eukarya (E). The statistics of extension types and sizes in the three kingdoms are based on extensions that are visible in the high-resolution ribosome structures (Supplementary Table 10). Although some extension size variability has been observed in phylogenetic studies^10,13,14,72^, the extensions identified in our structural analysis correspond to a representative extension set of each kingdom^12^.

### Phylogenetic analysis

Due to their complex evolutionary history^13,14^ r-proteins are difficult to align across kingdoms. Thus, r-proteins in their entirety (G + ext) were aligned in each kingdom separately, to characterize their conservation profiles. Eubacterial and archaeal r-proteins sequence alignments have been kindly provided by U. Wolf^72^. A representative dataset of eukaryotic r-proteins covering most of the eukaryotic taxons^47^ has been selected from NR and aligned with the program Muscle^73^. The aligned sequences have been then visualized, inspected and analysed with the program Jalview^74^. The quantitative values of the conservation profiles have been retrieved from the LOGOs to calculate the proportions of conserved residues at the PPi interfaces. Residues that occurred in the same position of more than 80% of the sequence were considered as conserved.

### Analysis of the inter-molecular contacts

The networks include all the protein–protein and protein-functional sites (PTC, tunnel, tRNA-sites, mRNA) interactions^4,46,75^. The intermolecular interactions constituting the networks have been calculated in the 3 kingdoms’ ribosome structures (Supplementary Table 10). The selected high-resolution structures correspond ribosomes trapped in the elongation cycle. Due to possible X-ray diffraction or cryo-electron-microscopy experimental errors, parts of the structures may be missing or not well defined in the PDB models. Hence, the networks determined here may have omitted a few interactions that have not been detected in the ribosome structures. Scripts systematically calculated the contacts using *areaimol* from CCP4^76^ with the default parameters (*diffmode:* “compare” and probe radius 1.4 Å) (Supplementary Fig. 4A). This provided an exhaustive list of protein–protein interactions (PPi) for each r-protein network. The distributions and the average values of the interface areas of the whole ribosome, large subunit (LSU), small subunit (SSU) networks shows that they remain globally tiny throughout the evolution (Supplementary Fig. 4B,C).

Most of the network contacts are “permanent” within the mature ribosome structure. However transient contacts occur during the elongation cycle. For example, interactions between the two-subunit bridges are formed and broken during the ratchet like motion (uL5-uS13 form different contacts in the two rotational states). In the current models, transiently bound translation factors (such as EF-Tu and EF-G in bacteria) are not included. The universal network described here is a minimal network that only contains the r-proteins conserved in the three kingdoms. However, there are several examples of r-proteins that occupy equivalent sites across kingdom (Supplementary Fig. 3). It is therefore possible that a precursor of such proteins also existed in the ancestral network.

### Network representations

To highlight the evolution of connectivity, the networks have been represented in two-dimensional cartoons where the evolutionary statuses of r-proteins, extensions and connections are indicated by different colours and representation codes (Supplementary Fig. 3). The interactions between r-protein and functional sites have been taken from the literature and from the present analysis^2–4^. All of the kingdom specific interactions have been listed (Supplementary Table 3). Simplified 3D network models have been used to describe the expansion of the network architecture during evolution. The centres of mass (COM) of the globular domains of interconnected proteins have been linked. The COM of r-proteins devoid of globular domains was calculated using the entire protein coordinates. The distances between the COM of each protein pairs of the network have been systematically calculated. Connections between the three tRNAs and the mRNA are drawn but are not reported in the statistics of r-protein connectivity.

### Graph theory analysis

To characterize the networks, several notions of centralities have been defined; centrality indices are a way to capture the centrality of a node in a graph and there are different definitions of centralities (degree, closeness, betweenness, eigenvector…) as there are different notions of “centrality” to capture. These quantities may be an indicator of the graph functionality as for example, whether a graph can transfer and treat information. In order to accept a hypothesis or claim, one needs to have strong evidence against the opposite claim. We therefore assumed that the networks we observed were nothing but random, in other words a typical realisation of a random graph (following an Erdős–Rényi model)^77,78^; it is our null hypothesis. By studying the law of the centralities under this hypothesis of randomness, we built a test statistic (and a test) that enabled us to decide if it is plausible that the networks we are studying are purely random or not. The answer is that one can reject the hypothesis that the networks are generated randomly (Supplementary Table 8). As one does not have access to the expression of the probability of the centralities under the null hypothesis, one needs to sample with respect to an Erdős–Rényi model the law of the test statistic and from there build a test. This leads to a new class of statistical hypothesis testing and the natural notion of coherence (all the details can be found in the Supplementary Graph Theory).

## Supplementary Information

Supplementary Information 1.

Supplementary Information 2.

Supplementary Information 3.

Supplementary Information 4.
