# Supplementary material for: Evolution of ribosomal protein network architectures
Source: Sci Rep. 2021 Jan 12;11:625. doi: 10.1038/s41598-020-80194-4 (PMC7804294; doi:10.1038/s41598-020-80194-4)
Supplement: Supplementary file 1 — Supplementary Information 1. [file 41598_2020_80194_MOESM1_ESM.pdf]

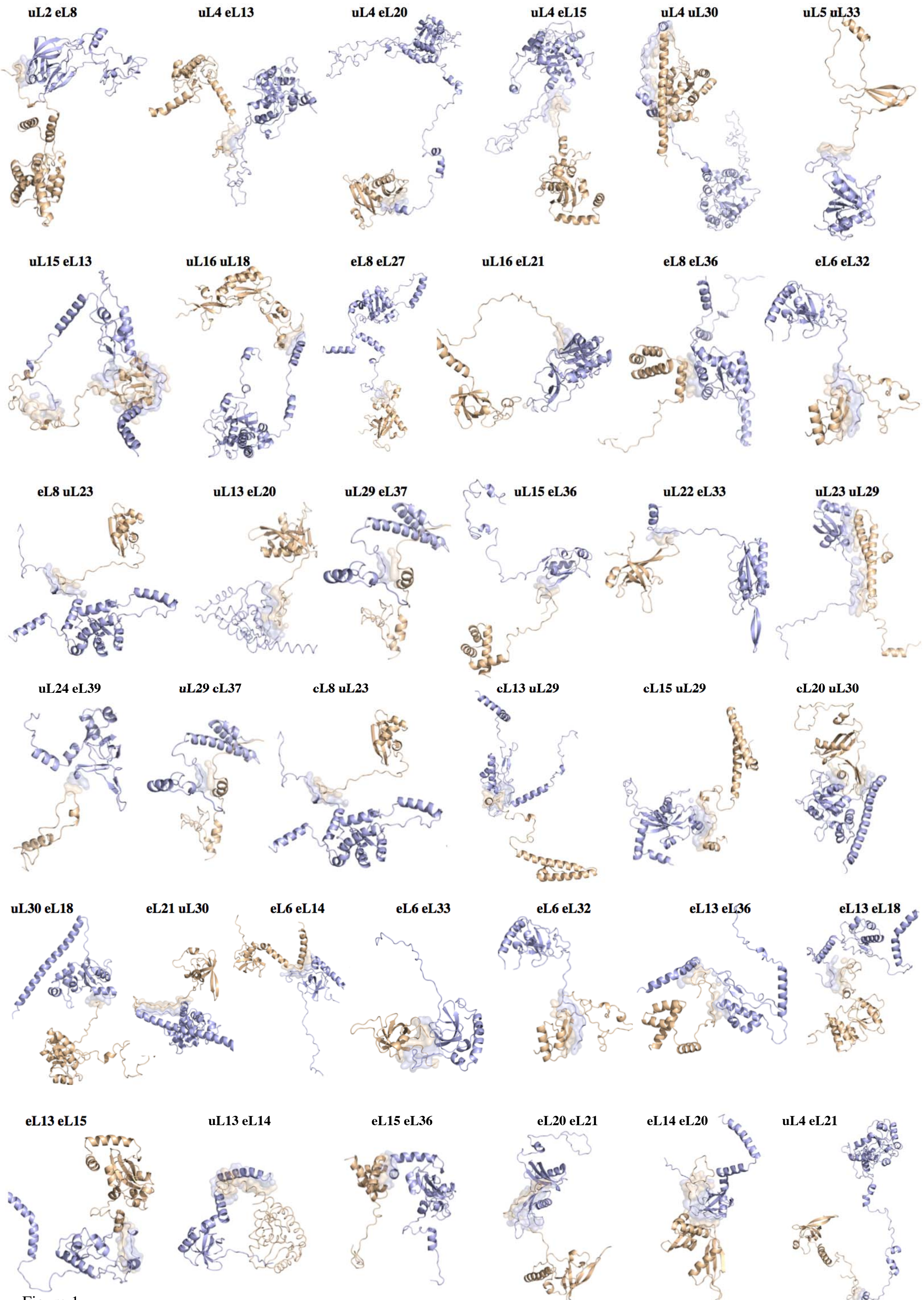
uL2 eL8
uL4 eL13
uL4 eL20
uL4 eL15
uL4 uL30
uL5 uL33
uL15 eL13
uL16 uL18
eL8 eL27
uL16 eL21
eL8 eL36
eL6 eL32
eL8 uL23
uL13 eL20
uL29 eL37
uL15 eL36
uL22 eL33
uL23 uL29
uL24 eL39
uL29 cL37
cL8 uL23
cL13 uL29
cL15 uL29
cL20 uL30
uL30 eL18
eL21 uL30
eL6 eL14
eL6 eL33
eL6 eL32
eL13 eL36
eL13 eL18
eL13 eL15
uL13 eL14
eL15 eL36
eL20 eL21
eL14 eL20
uL4 eL21

Figure 1

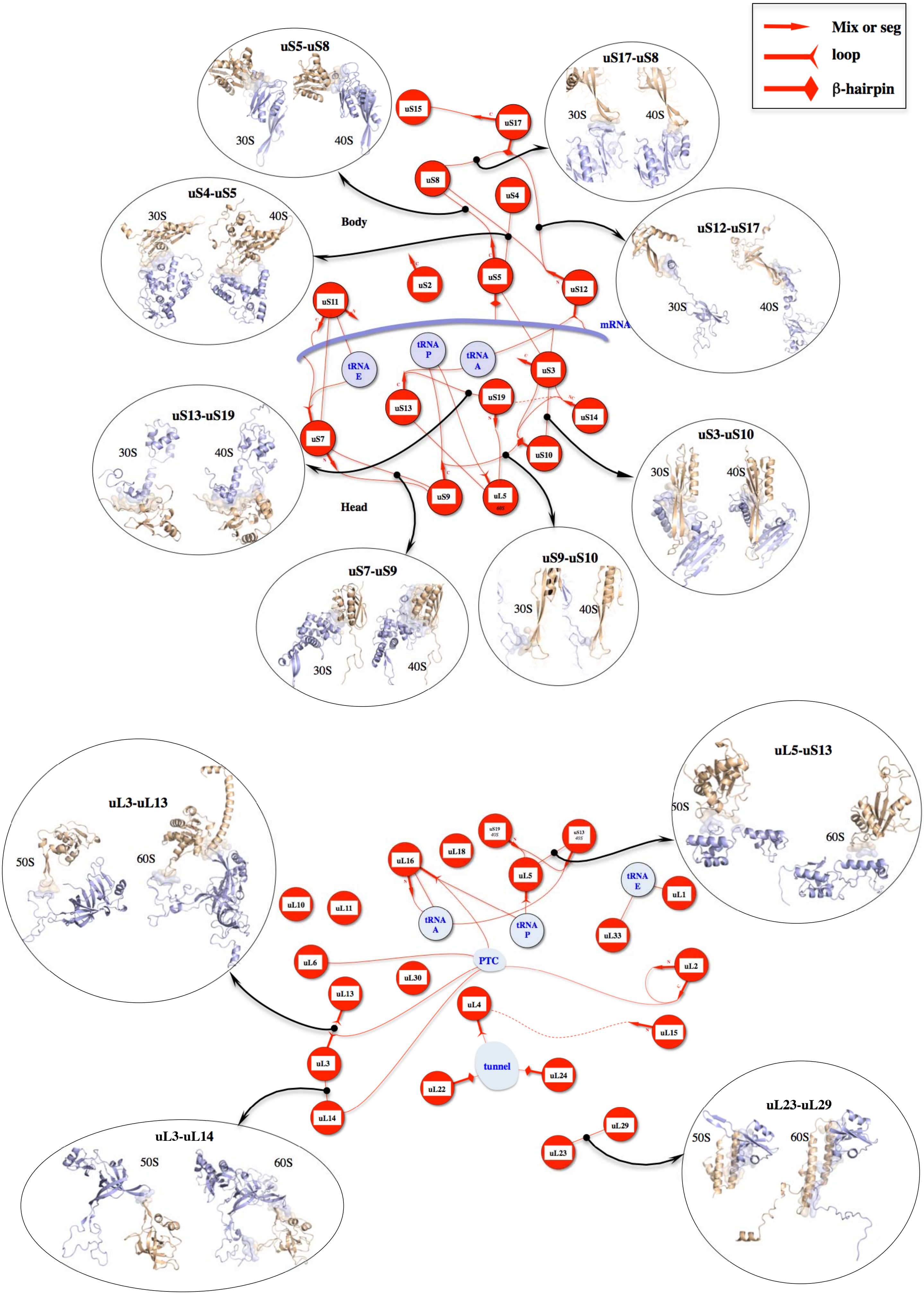

Mix or seg
loop
β-hairpin
uS5-uS8
30S
40S
uS17-uS8
30S
40S
uS4-uS5
30S
40S
uS12-uS17
30S
40S
Body
uS15
uS17
uS8
uS4
uS2
uS5
uS12
uS11
mRNA
tRNA P
tRNA A
tRNA E
uS3
uS13
uS19
uS14
uS7
uS10
uS9
uL5
60S
uS13-uS19
30S
40S
uS3-uS10
30S
40S
Head
uS9-uS10
30S
40S
uS7-uS9
30S
40S
uL3-uL13
50S
60S
uL5-uS13
50S
60S
uS19
40S
uS13
40S
uL16
uL18
uL5
tRNA E
uL1
uL10
uL11
tRNA A
tRNA P
uL33
uL6
PTC
uL2
uL30
uL13
uL4
uL15
uL3
tunnel
uL22
uL24
uL14
uL29
uL23
uL3-uL14
50S
60S
uL23-uL29
50S
60S

Figure 2

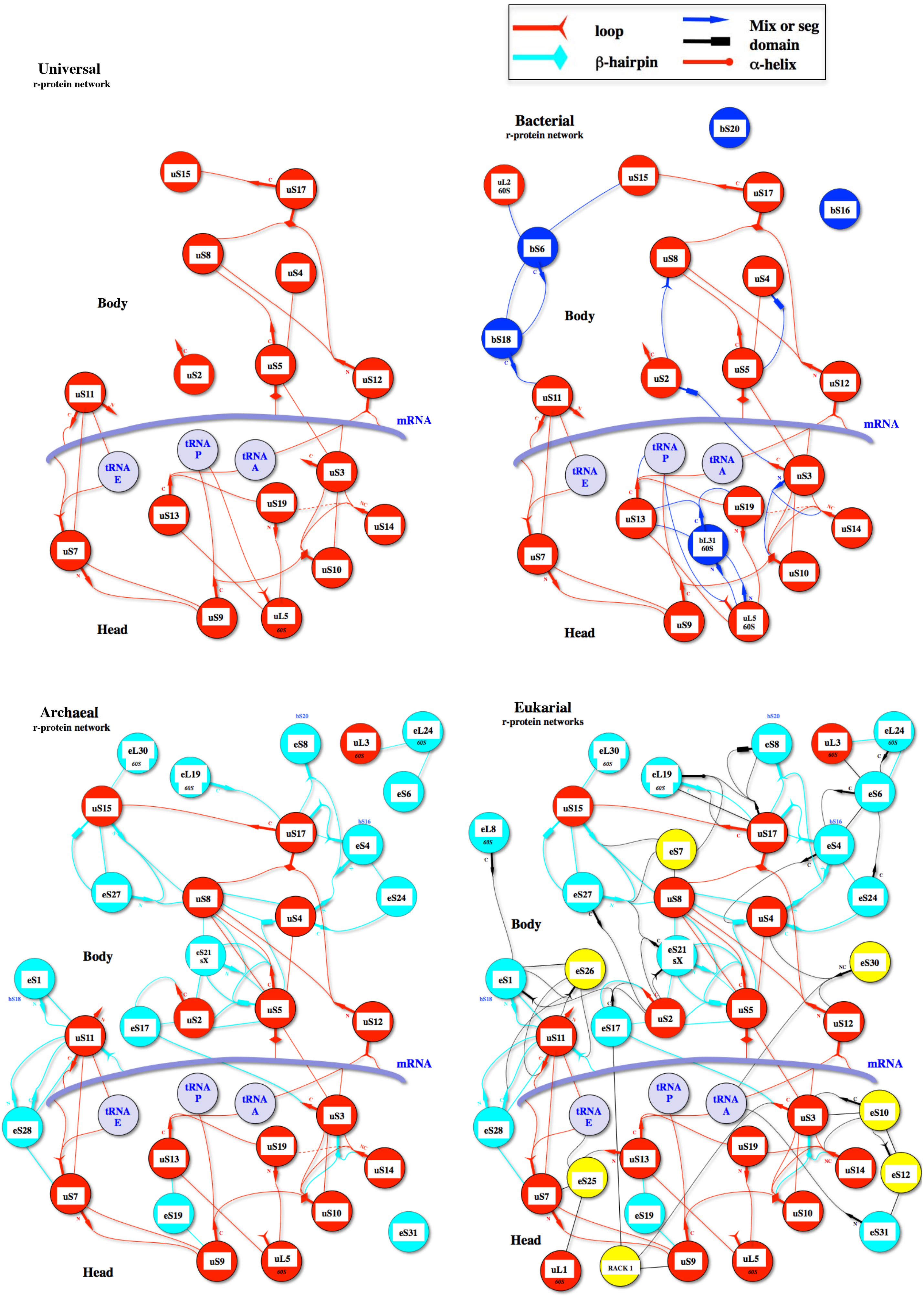
loop
β-hairpin
Mix or seg
domain
α-helix
Universal
r-protein network
Body
Head
mRNA
tRNA E
tRNA P
tRNA A
uS15
uS17
uS8
uS4
uS2
uS5
uS12
uS11
uS3
uS13
uS19
uS14
uS7
uS10
uS9
uL5 60S
Bacterial
r-protein network
bS20
bS16
uL2 60S
bS6
bS18
bL31 60S
Archaeal
r-protein network
eL30 60S
eL19 60S
eS8
uL3 60S
eL24 60S
eS6
eS4
eS27
eS24
eS21 sX
eS1
eS17
eS28
eS19
eS31
bS20
bS16
bS18
Eukarial
r-protein networks
eL8 60S
eS7
eS26
eS30
eS10
eS12
eS25
uL1 60S
RACK 1

Figure 3

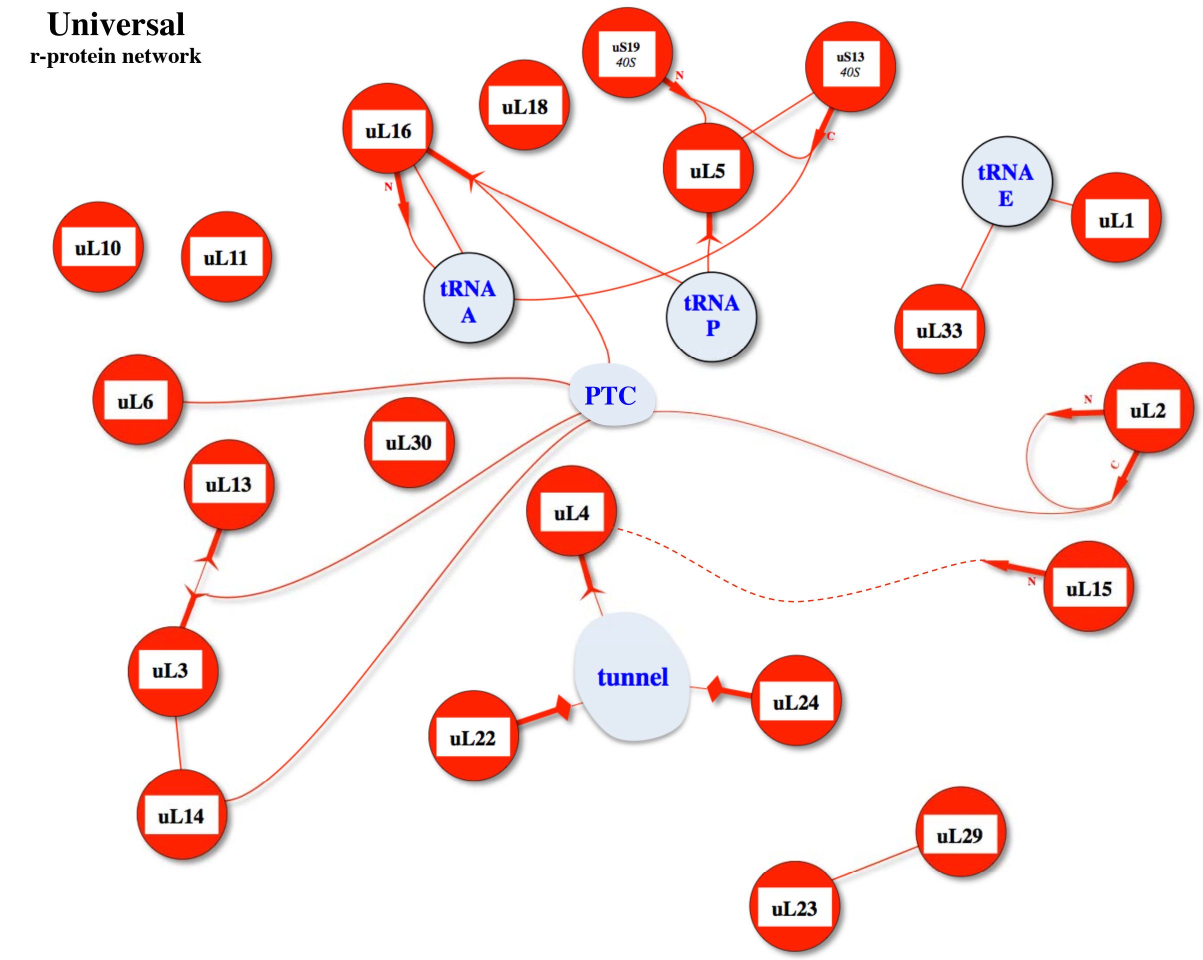

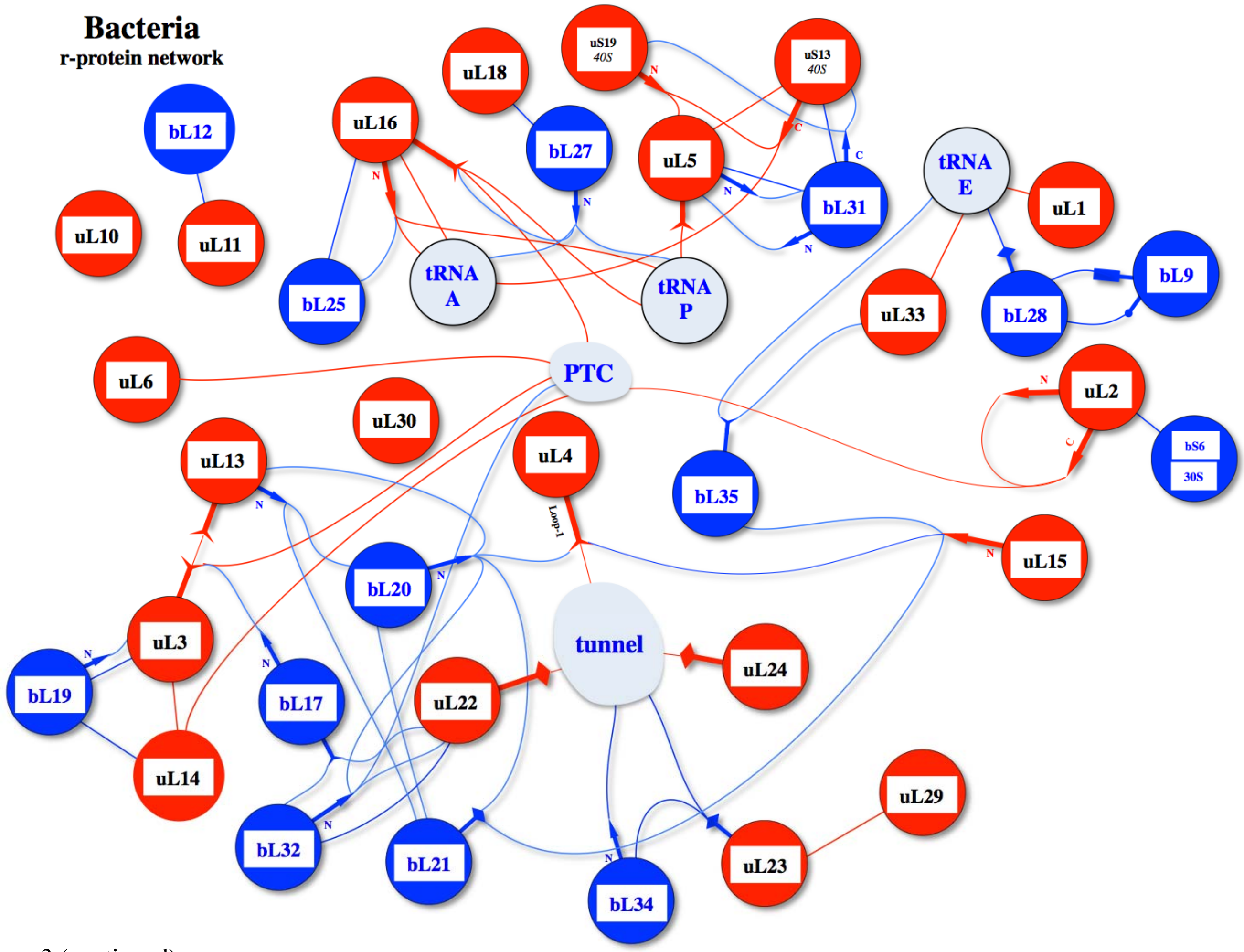

Figure 3 (continued)

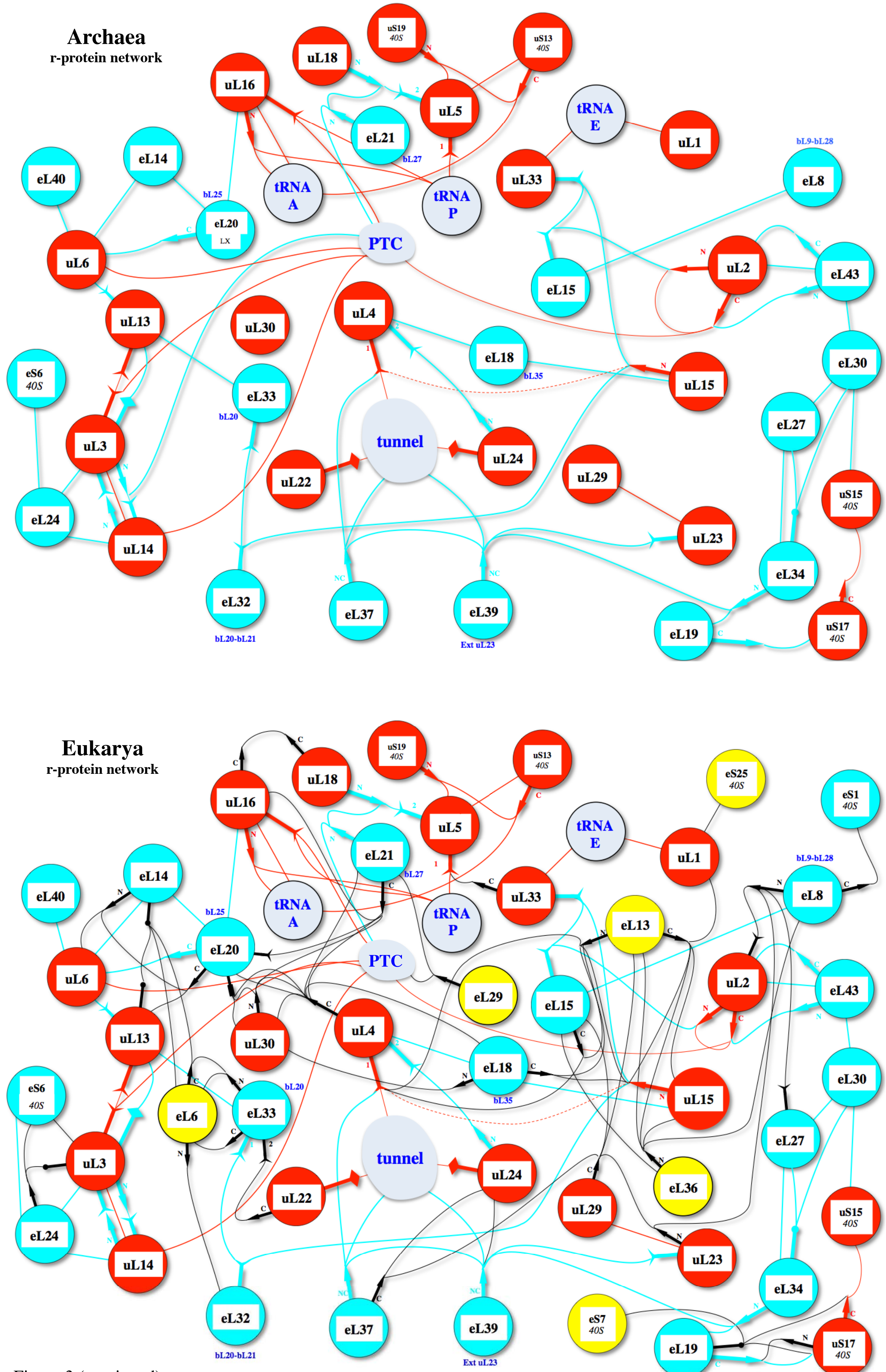

Figure 3 (continued)

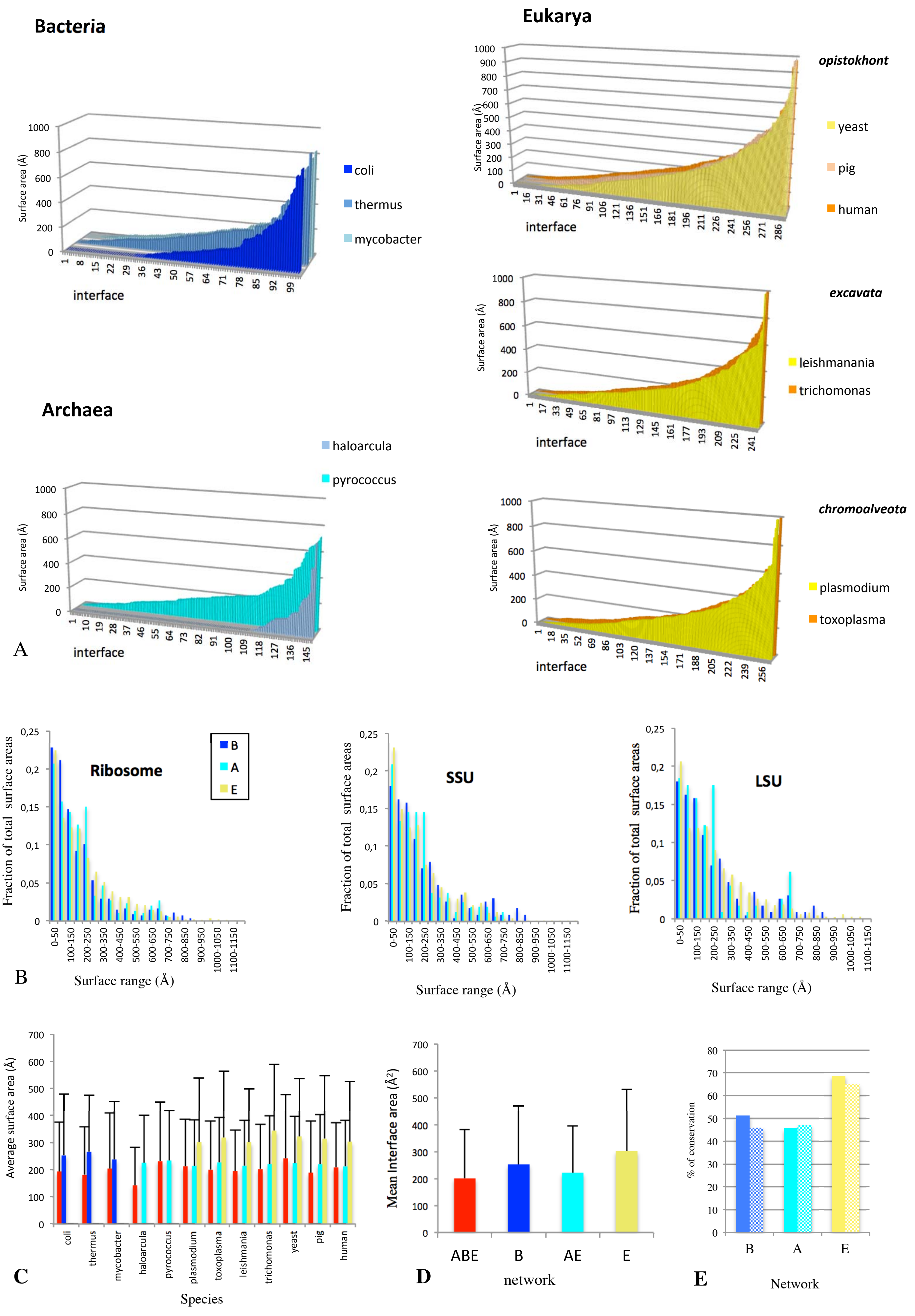
Bacteria
Surface area (Å)
interface
coli
thermus
mycobacter
Eukarya
opistokhont
yeast
pig
human
excavata
leishmanania
trichomonas
chromoalveota
plasmodium
toxoplasma
Archaea
haloarcula
pyrococcus
A
Ribosome
SSU
LSU
B
A
E
Fraction of total surface areas
Surface range (Å)
Average surface area (Å)
Species
C
Mean Interface area (Å²)
ABE
B
AE
E
network
D
% of conservation
Network
E

Figure 4
