# Supplementary material for: Evolution of ribosomal protein network architectures
Source: Sci Rep. 2021 Jan 12;11:625. doi: 10.1038/s41598-020-80194-4 (PMC7804294; doi:10.1038/s41598-020-80194-4)
Supplement: Supplementary file 2 — Supplementary Information 2. [file 41598_2020_80194_MOESM2_ESM.pdf]

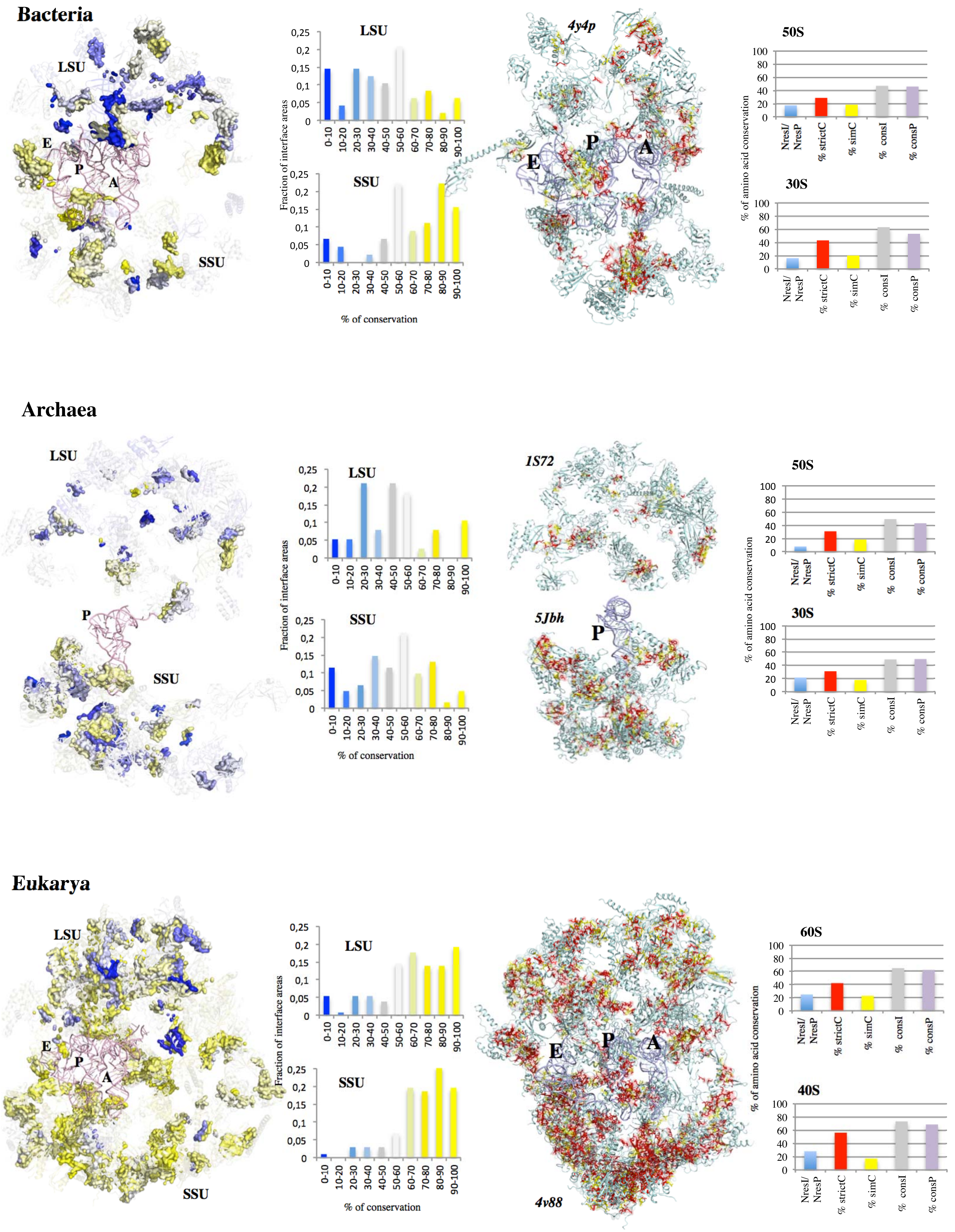
Bacteria
LSU
E
P
A
SSU
LSU
SSU
Fraction of interface areas
0,25
0,2
0,15
0,1
0,05
0
0-10
10-20
20-30
30-40
40-50
50-60
60-70
70-80
80-90
90-100
% of conservation
4y4p
E
P
A
50S
30S
% of amino acid conservation
100
80
60
40
20
0
NresI/ NresP
% strictC
% simC
% consI
% consP
Archaea
LSU
P
SSU
LSU
SSU
Fraction of interface areas
% of conservation
1S72
5Jbh
P
50S
30S
% of amino acid conservation
NresI/ NresP
% strictC
% simC
% consI
% consP
Eukarya
LSU
E
P
A
SSU
LSU
SSU
Fraction of interface areas
% of conservation
E
P
A
4v88
60S
40S
% of amino acid conservation
NresI/ NresP
% strictC
% simC
% consI
% consP

Figure 5

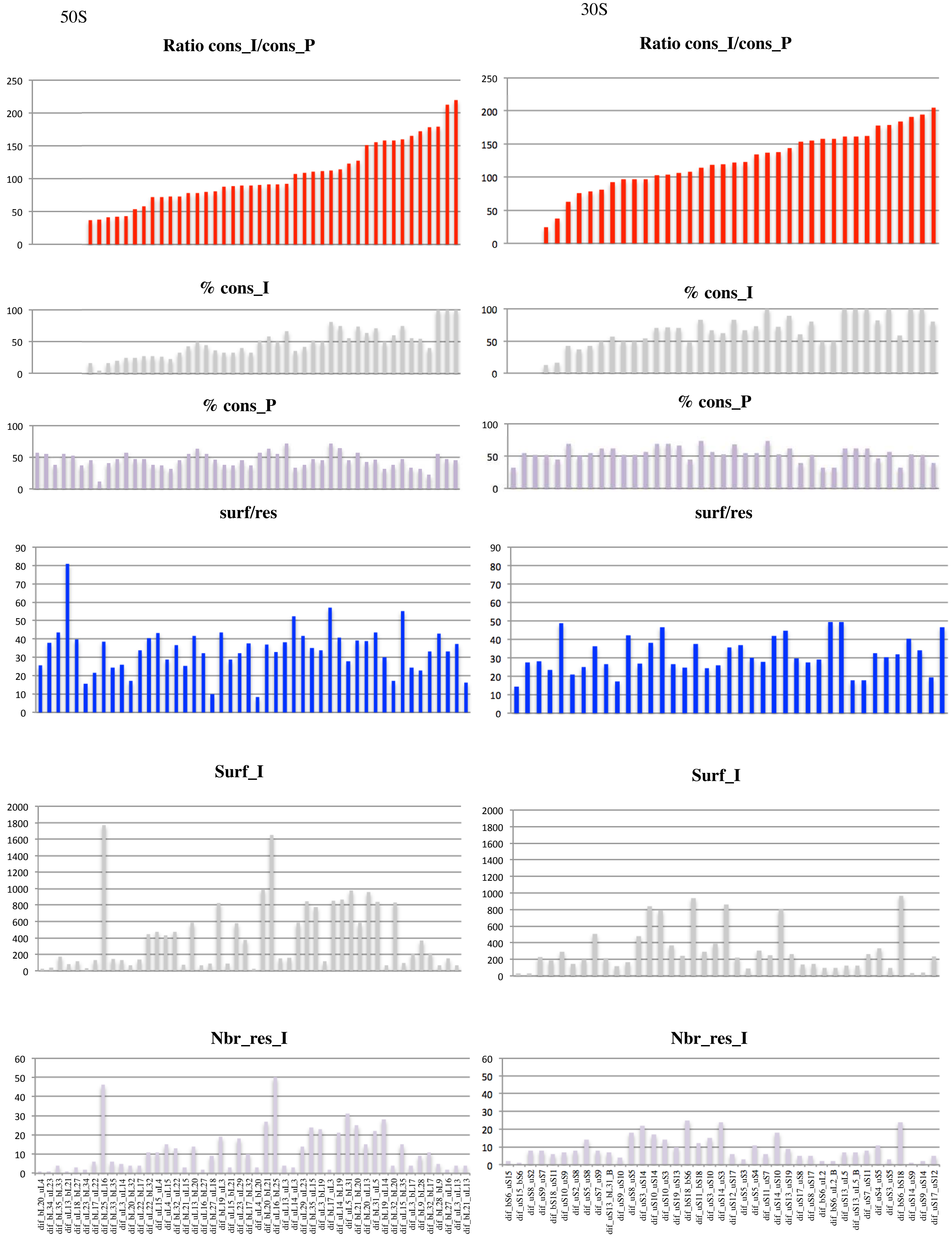
50S
Ratio cons_I/cons_P
% cons_I
% cons_P
surf/res
Surf_I
Nbr_res_I
30S
Ratio cons_I/cons_P
% cons_I
% cons_P
surf/res
Surf_I
Nbr_res_I

Figure 6

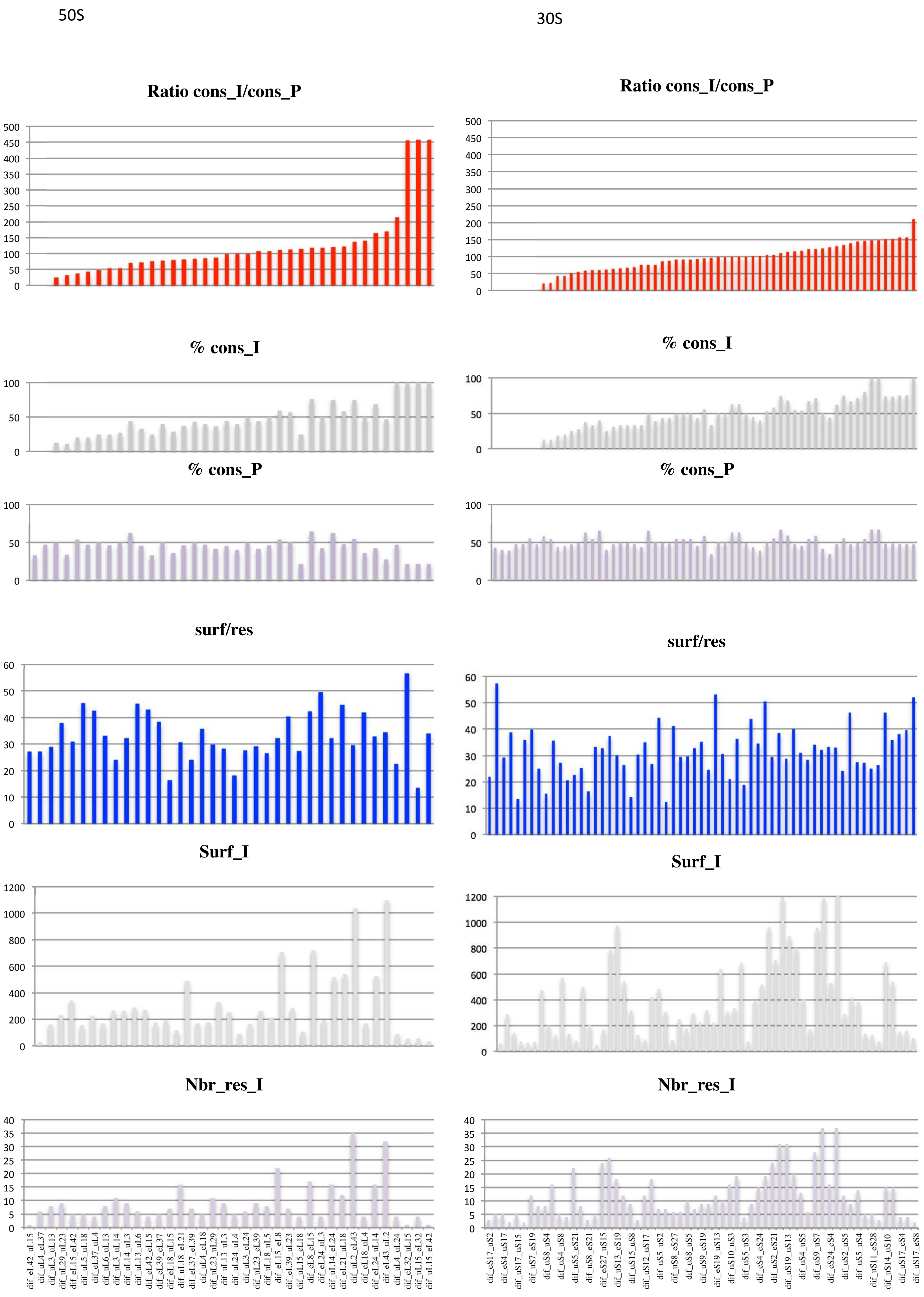
50S
30S
Ratio cons_I/cons_P
% cons_I
% cons_P
surf/res
Surf_I
Nbr_res_I

Figure 7

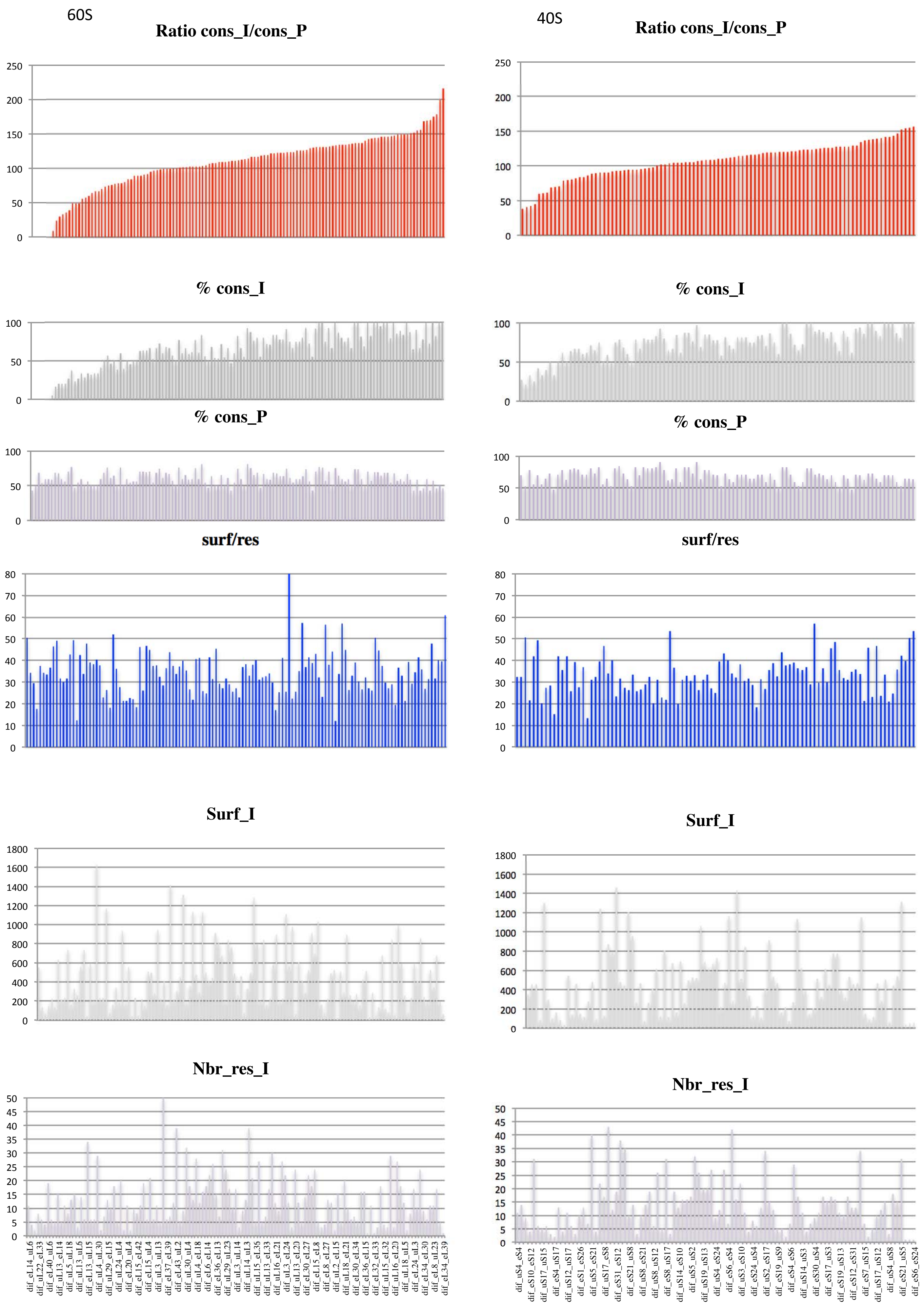
60S
Ratio cons_I/cons_P
% cons_I
% cons_P
surf/res
Surf_I
Nbr_res_I
40S
Ratio cons_I/cons_P
% cons_I
% cons_P
surf/res
Surf_I
Nbr_res_I

Figure 8

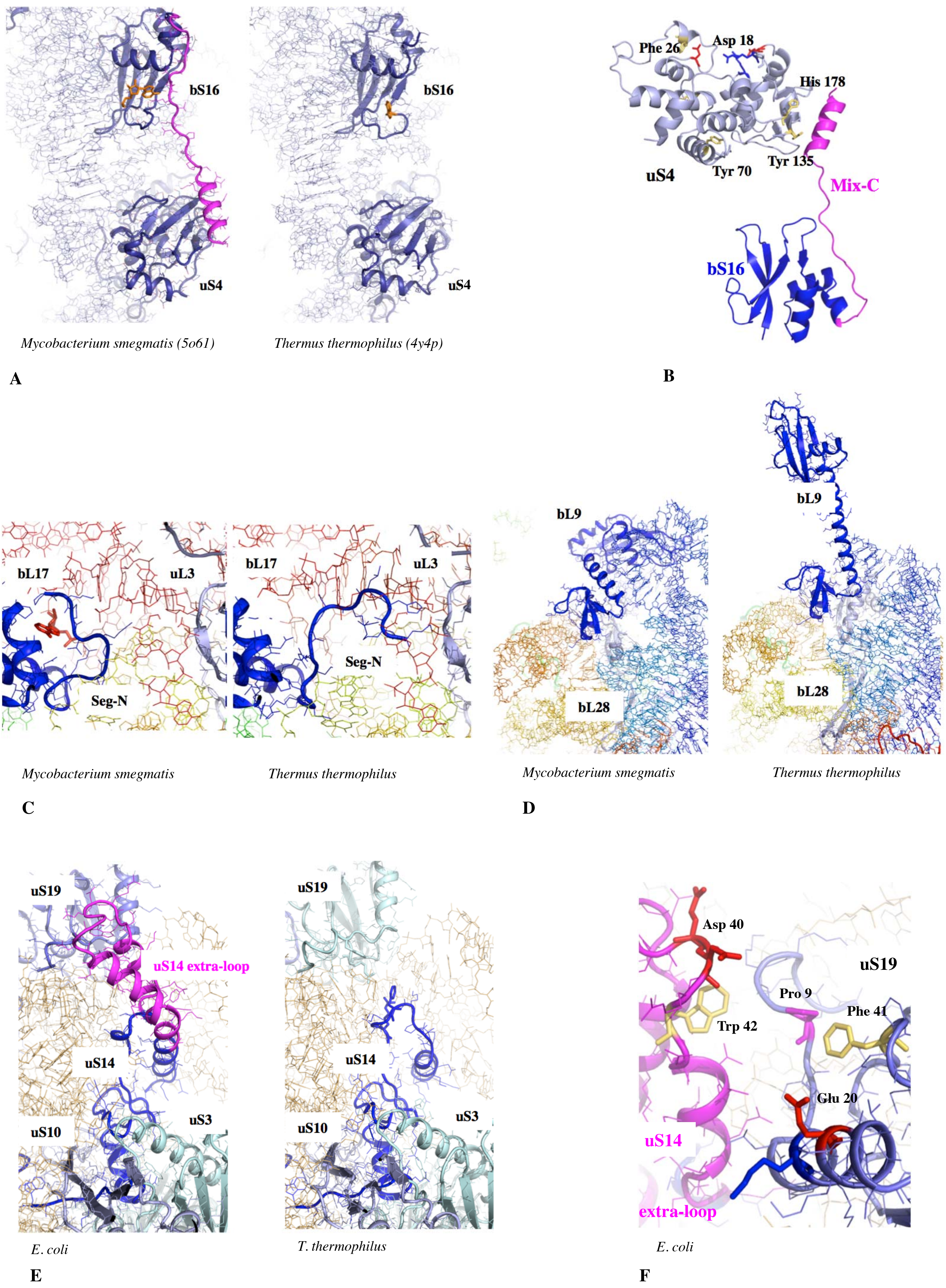
bS16
uS4
bS16
uS4
Mycobacterium smegmatis (5o61)
Thermus thermophilus (4y4p)
A
Phe 26
Asp 18
His 178
uS4
Tyr 70
Tyr 135
Mix-C
bS16
B
bL17
uL3
Seg-N
bL17
uL3
Seg-N
Mycobacterium smegmatis
Thermus thermophilus
C
bL9
bL28
bL9
bL28
Mycobacterium smegmatis
Thermus thermophilus
D
uS19
uS14 extra-loop
uS14
uS10
uS3
uS19
uS14
uS10
uS3
E. coli
T. thermophilus
E
Asp 40
uS19
Pro 9
Phe 41
Trp 42
Glu 20
uS14
extra-loop
E. coli
F

Figure 9

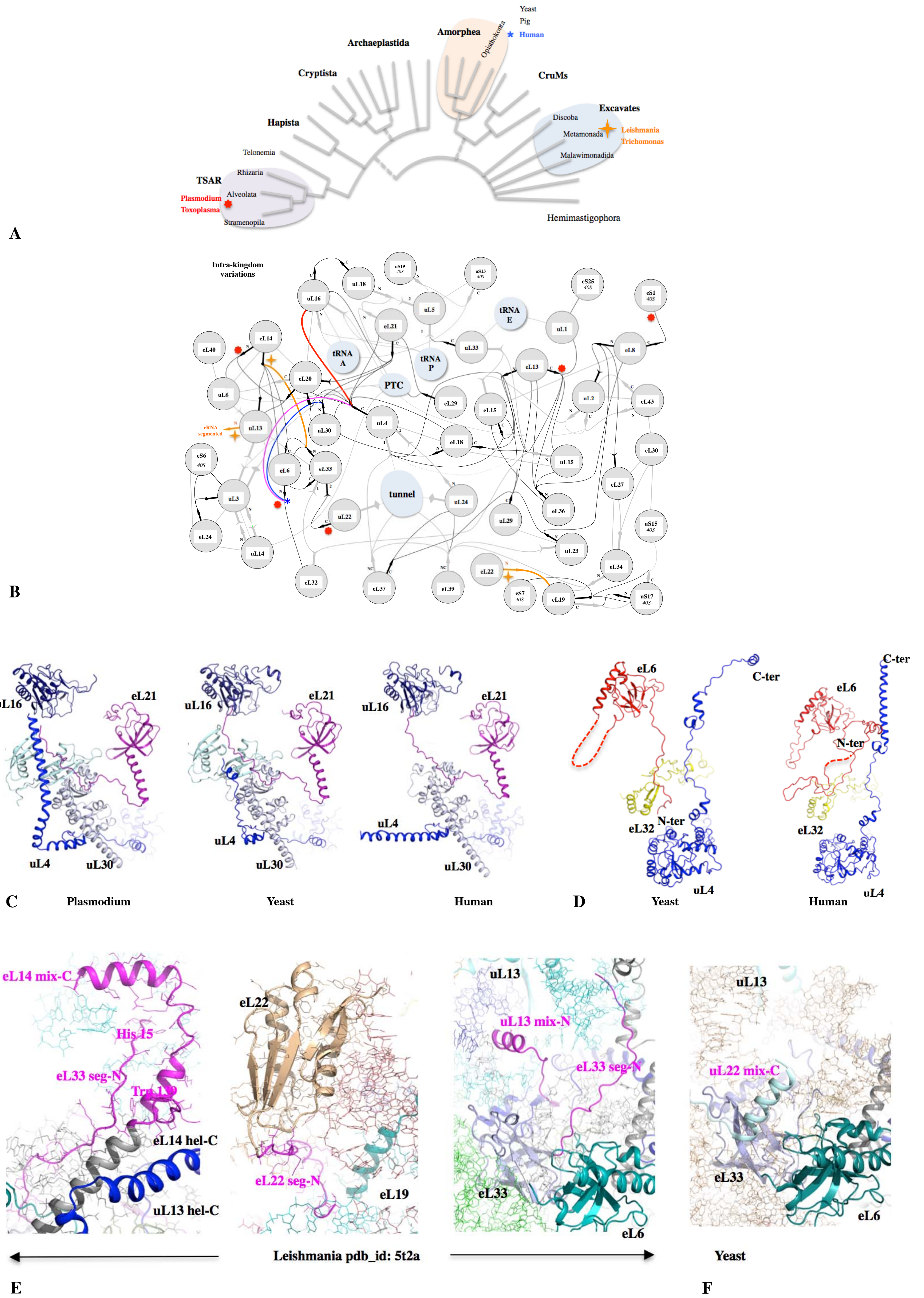
A
B
C
D
E
F
Yeast
Pig
Human
Amorphea
Opisthokonta
Archaeplastida
Cryptista
CruMs
Excavates
Discoba
Metamonada
Leishmania
Trichomonas
Malawimonadida
Hapista
Telonemia
Rhizaria
TSAR
Alveolata
Plasmodium
Toxoplasma
Stramenopila
Hemimastigophora
Intra-kingdom variations
tRNA A
tRNA P
tRNA E
PTC
tunnel
rRNA segmented
Plasmodium
Yeast
Human
uL16
eL21
uL4
uL30
eL6
eL32
C-ter
N-ter
eL14 mix-C
His 15
eL33 seg-N
eL14 hel-C
uL13 hel-C
eL22
eL22 seg-N
eL19
uL13
uL13 mix-N
eL33
uL22 mix-C
Leishmania pdb_id: 5t2a

Figure 10

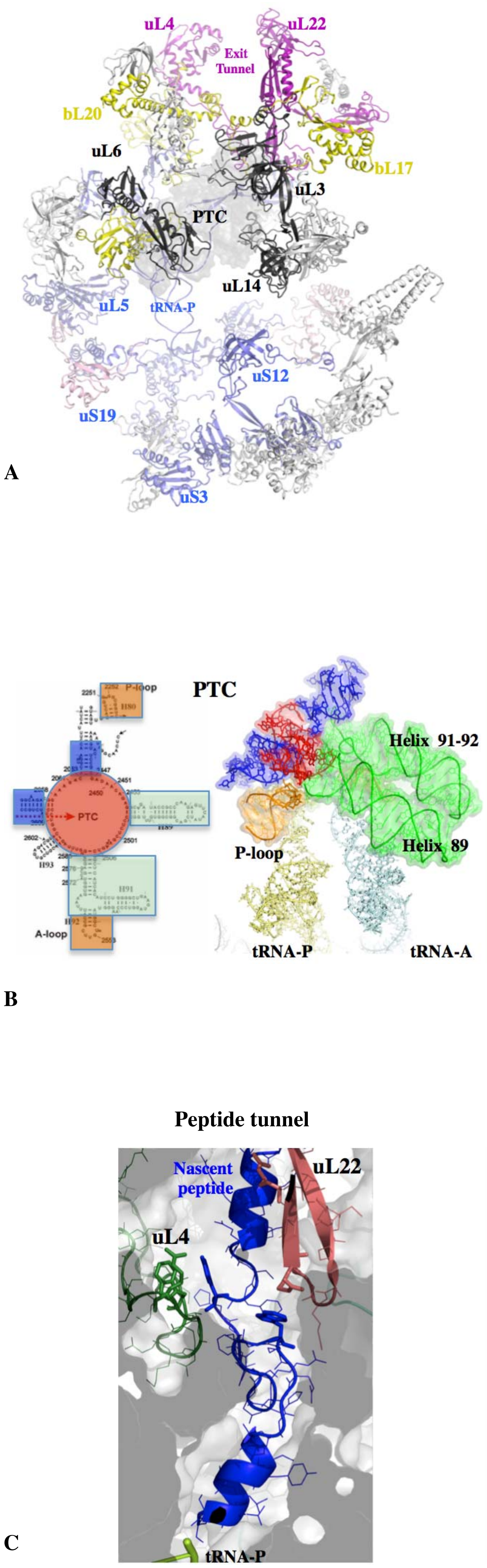
uL4
uL22
Exit Tunnel
bL20
bL17
uL6
uL3
PTC
uL14
uL5
tRNA-P
uS12
uS19
uS3
A
PTC
P-loop
A-loop
H93
Helix 91-92
Helix 89
P-loop
tRNA-P
tRNA-A
B
Peptide tunnel
Nascent peptide
uL22
uL4
tRNA-P
C
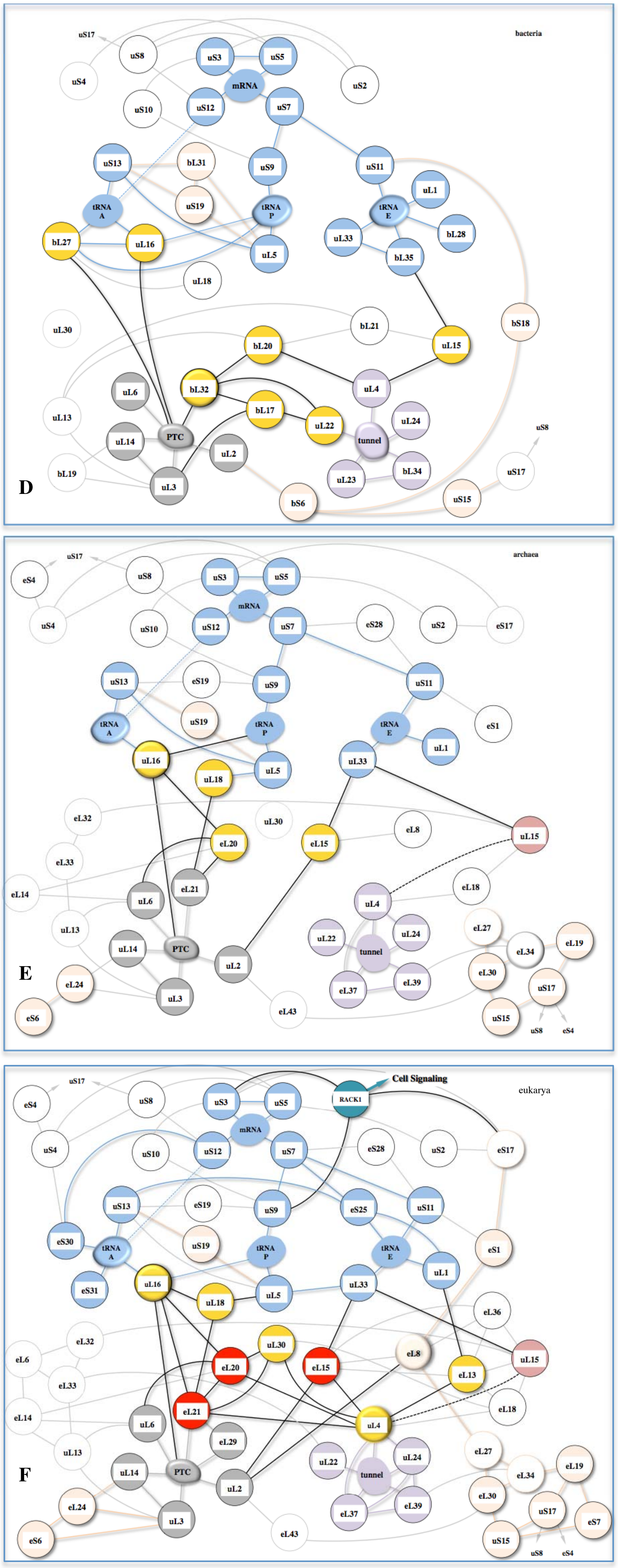
bacteria
D
archaea
E
eukarya
Cell Signaling
RACK1
F
mRNA
tRNA A
tRNA P
tRNA E
PTC
tunnel

Figure 11

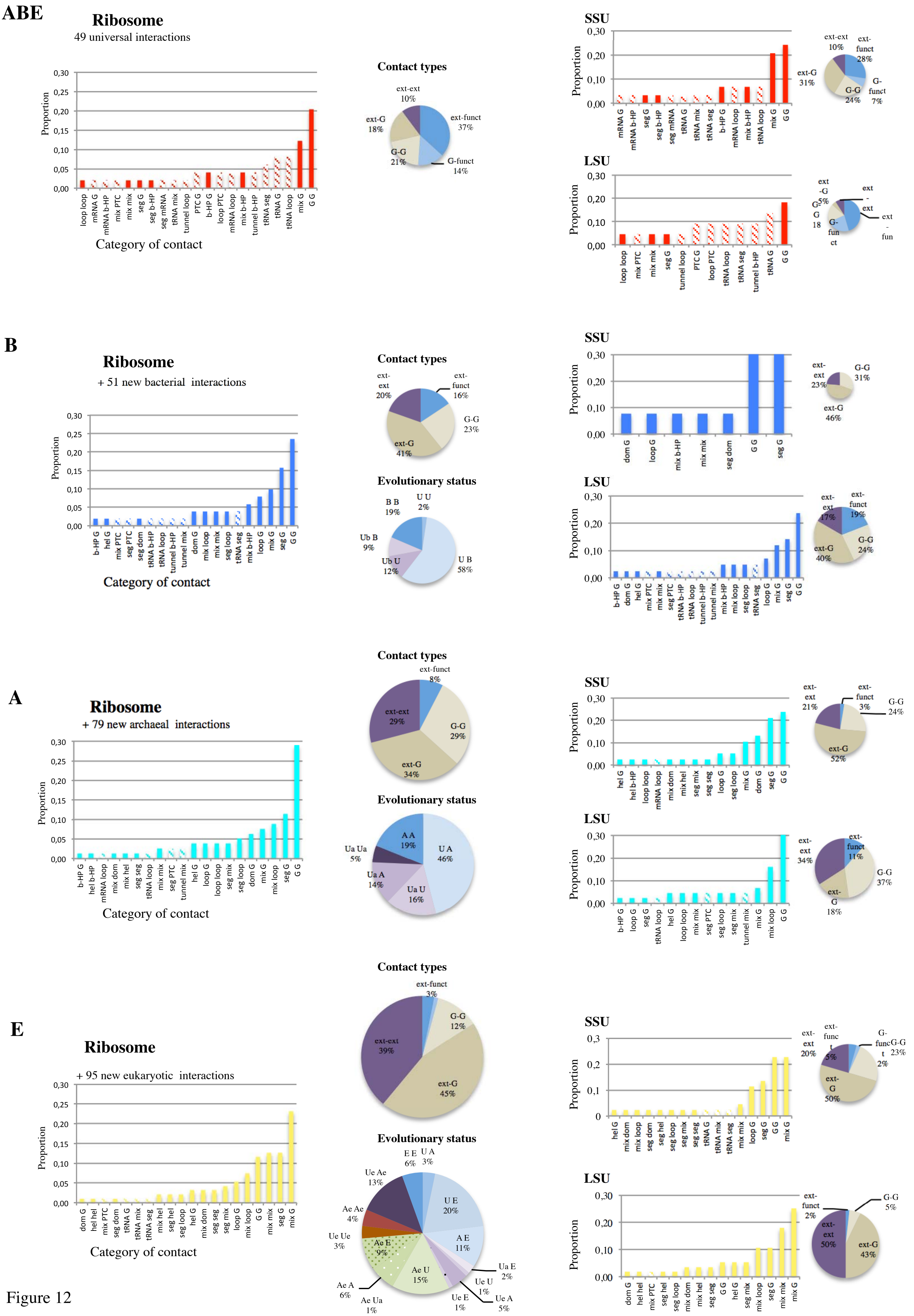
ABE
Ribosome
49 universal interactions
Proportion
Category of contact
Contact types
ext-ext 10%
ext-funct 37%
ext-G 18%
G-G 21%
G-funct 14%
SSU
LSU
B
Ribosome
+ 51 new bacterial interactions
Evolutionary status
B B 19%
U U 2%
Ub B 9%
Ub U 12%
U B 58%
A
Ribosome
+ 79 new archaeal interactions
ext-funct 8%
ext-ext 29%
G-G 29%
ext-G 34%
A A 19%
U A 46%
Ua Ua 5%
Ua A 14%
Ua U 16%
E
Ribosome
+ 95 new eukaryotic interactions
ext-funct 3%
G-G 12%
ext-ext 39%
ext-G 45%
E E 6%
U A 3%
Ue Ae 13%
U E 20%
Ae Ae 4%
A E 11%
Ue Ue 3%
Ae E 9%
Ae U 15%
Ua E 2%
Ue U 1%
Ae A 6%
Ae Ua 1%
Ue E 1%
Ue A 5%

Figure 12

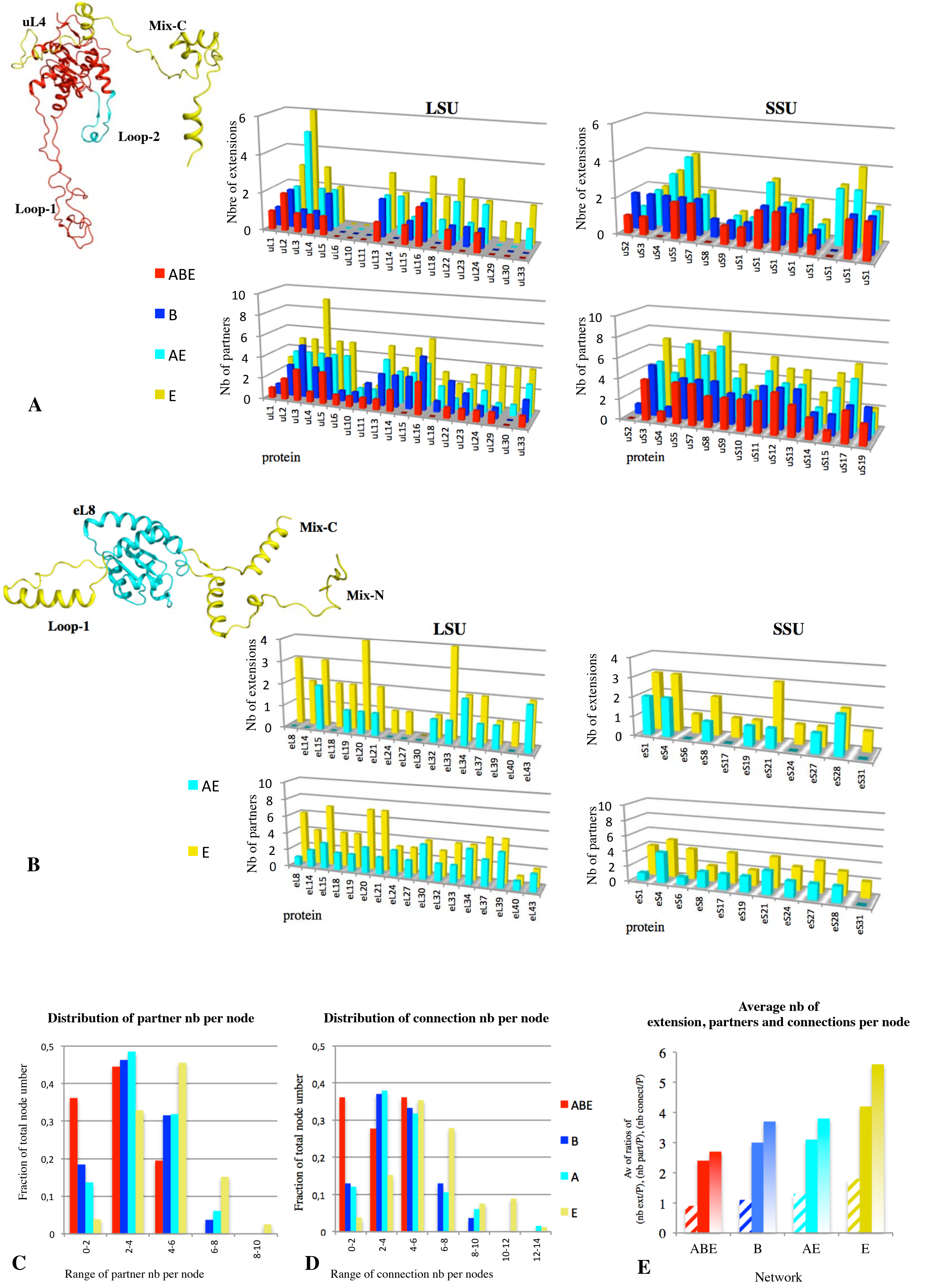

uL4
Mix-C
Loop-2
Loop-1
LSU
SSU
Nbre of extensions
Nb of partners
protein
ABE
B
AE
E
A
uL1
uL2
uL3
uL4
uL5
uL6
uL10
uL11
uL13
uL14
uL15
uL16
uL18
uL22
uL23
uL24
uL29
uL30
uL33
uS2
uS3
uS4
uS5
uS7
uS8
uS9
uS10
uS11
uS12
uS13
uS14
uS15
uS17
uS19
eL8
Mix-C
Mix-N
Loop-1
Nb of extensions
B
eL8
eL14
eL15
eL18
eL19
eL20
eL21
eL24
eL27
eL30
eL32
eL33
eL34
eL37
eL39
eL40
eL43
eS1
eS4
eS6
eS8
eS17
eS19
eS21
eS24
eS27
eS28
eS31
Distribution of partner nb per node
Fraction of total node umber
Range of partner nb per node
C
Distribution of connection nb per node
Fraction of total node umber
Range of connection nb per nodes
D
Average nb of
extension, partners and connections per node
Av of ratios of
(nb ext/P), (nb part/P), (nb conect/P)
Network
E

Figure 13

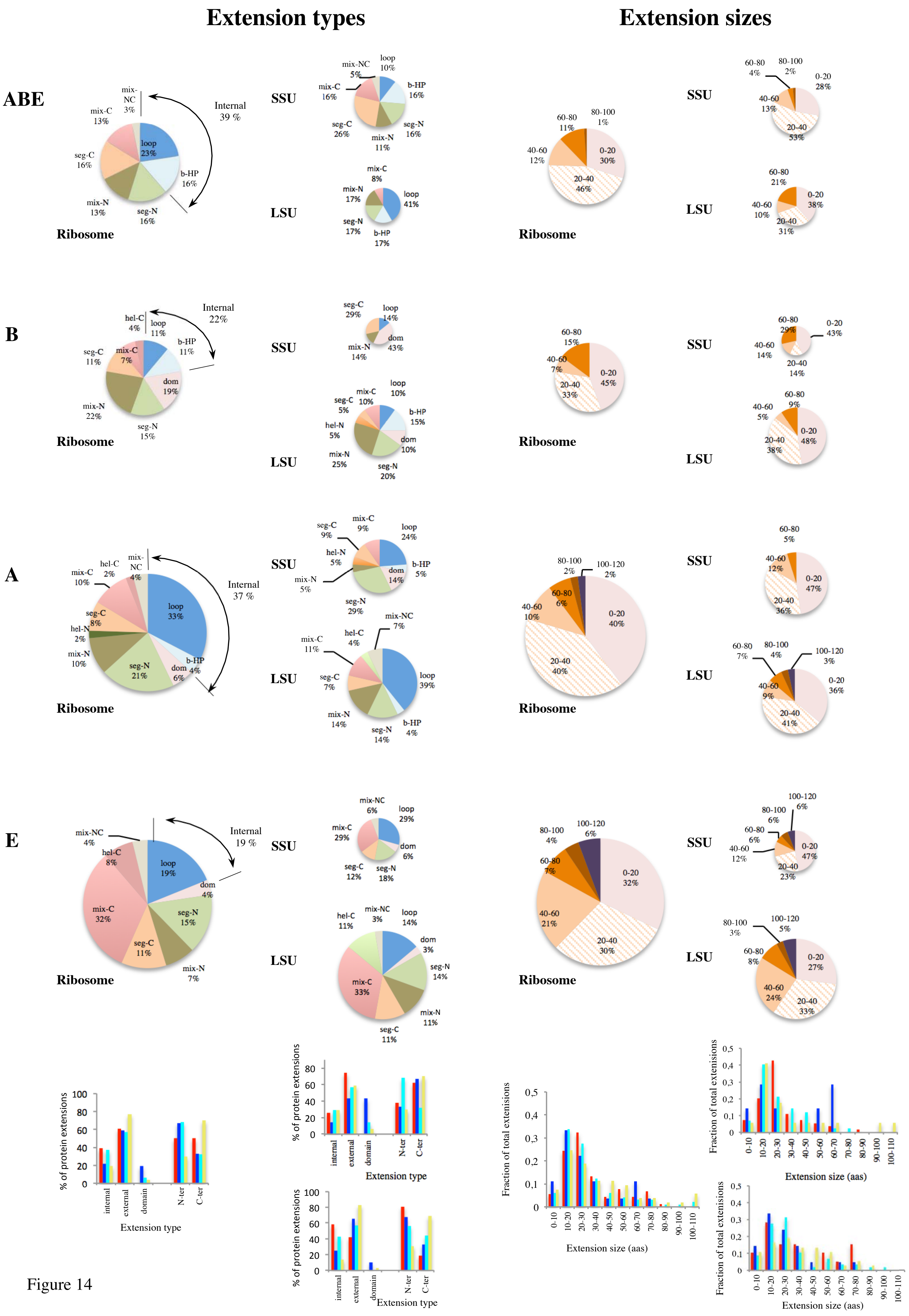
Extension types
Extension sizes
ABE
B
A
E
Ribosome
SSU
LSU
Internal 39 %
Internal 22%
Internal 37 %
Internal 19 %
loop 23%
b-HP 16%
seg-N 16%
mix-N 13%
seg-C 16%
mix-C 13%
mix-NC 3%
0-20 30%
20-40 46%
40-60 12%
60-80 11%
80-100 1%
% of protein extensions
internal
external
domain
N-ter
C-ter
Extension type
Fraction of total extensions
Extension size (aas)
0-10
10-20
20-30
30-40
40-50
50-60
60-70
70-80
80-90
90-100
100-110

Figure 14

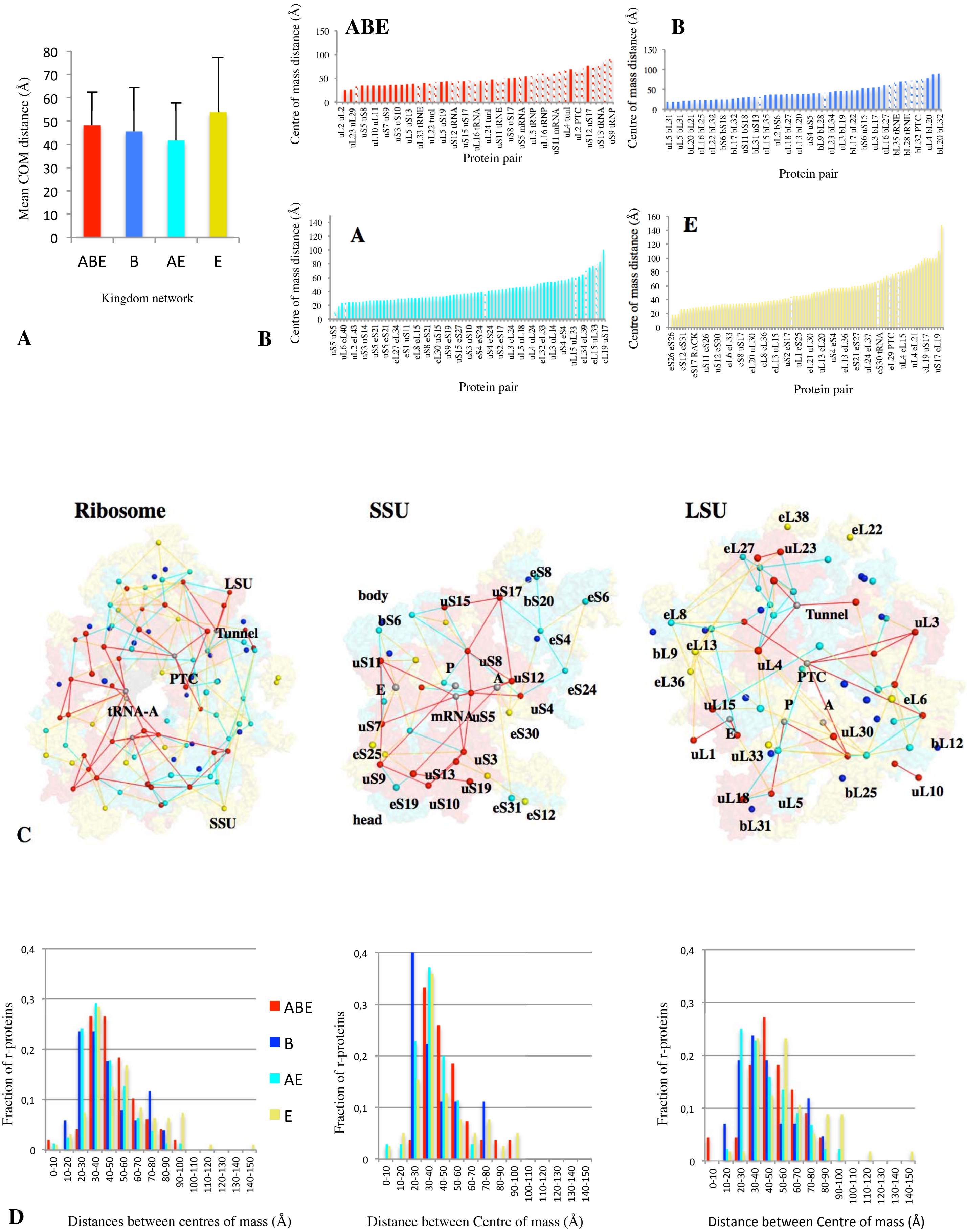
Mean COM distance (Å)
ABE
B
AE
E
Kingdom network
A
B
Centre of mass distance (Å)
Protein pair
ABE
B
A
E
C
Ribosome
LSU
Tunnel
PTC
tRNA-A
SSU
body
head
mRNA
P
A
E
uS5
uS15
uS17
bS20
eS8
eS6
bS6
uS8
uS12
eS4
eS24
uS11
uS4
eS30
uS7
eS25
uS3
uS9
uS13
uS19
eS19
uS10
eS31
eS12
eL38
eL22
eL27
uL23
eL8
uL3
bL9
eL13
uL4
eL36
uL15
uL1
uL33
uL30
eL6
bL12
uL18
uL5
bL25
uL10
bL31
D
Fraction of r-proteins
Distances between centres of mass (Å)
Distance between Centre of mass (Å)

Figure S15
