# Supplementary material for: Evolution of ribosomal protein network architectures
Source: Sci Rep. 2021 Jan 12;11:625. doi: 10.1038/s41598-020-80194-4 (PMC7804294; doi:10.1038/s41598-020-80194-4)
Supplement: Supplementary file 3 — Supplementary Information 3. [file 41598_2020_80194_MOESM3_ESM.pdf]

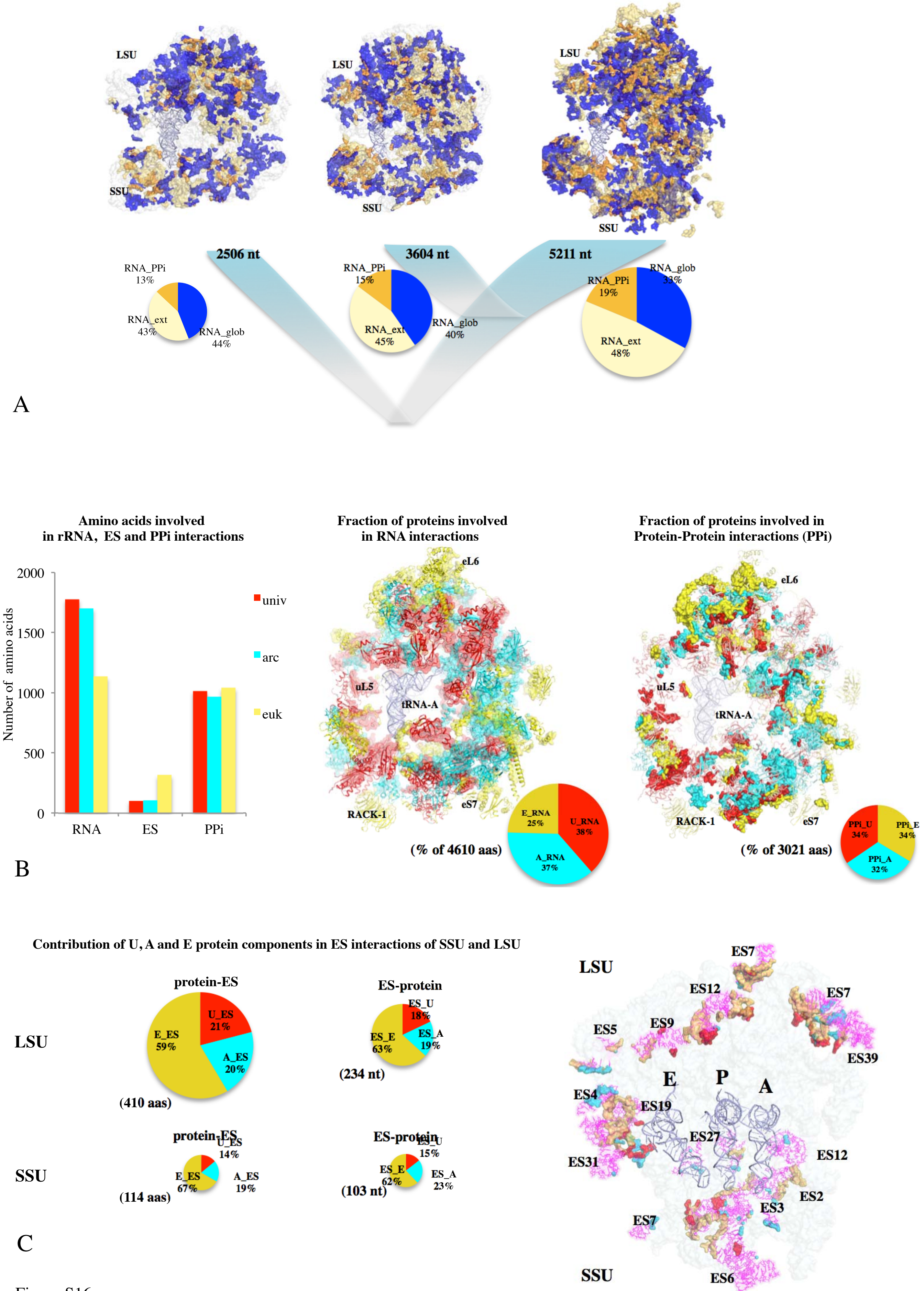

LSU
SSU
LSU
SSU
LSU
SSU
2506 nt
3604 nt
5211 nt
RNA_PPi 13%
RNA_ext 43%
RNA_glob 44%
RNA_PPi 15%
RNA_ext 45%
RNA_glob 40%
RNA_PPi 19%
RNA_glob 33%
RNA_ext 48%
A
Amino acids involved in rRNA, ES and PPi interactions
Number of amino acids
2000
1500
1000
500
0
RNA
ES
PPi
univ
arc
euk
Fraction of proteins involved in RNA interactions
eL6
uL5
tRNA-A
RACK-1
eS7
E_RNA 25%
U_RNA 38%
A_RNA 37%
(% of 4610 aas)
Fraction of proteins involved in Protein-Protein interactions (PPi)
eL6
uL5
tRNA-A
RACK-1
eS7
PPi_U 34%
PPi_E 34%
PPi_A 32%
(% of 3021 aas)
B
Contribution of U, A and E protein components in ES interactions of SSU and LSU
protein-ES
ES-protein
LSU
U_ES 21%
E_ES 59%
A_ES 20%
(410 aas)
ES_U 18%
ES_E 63%
ES_A 19%
(234 nt)
SSU
protein-ES
U_ES 14%
E_ES 67%
A_ES 19%
(114 aas)
ES-protein
ES_U 15%
ES_E 62%
ES_A 23%
(103 nt)
C
LSU
ES7
ES12
ES7
ES5
ES9
ES39
E
P
A
ES4
ES19
ES27
ES31
ES12
ES2
ES3
ES7
SSU
ES6

Figure S16

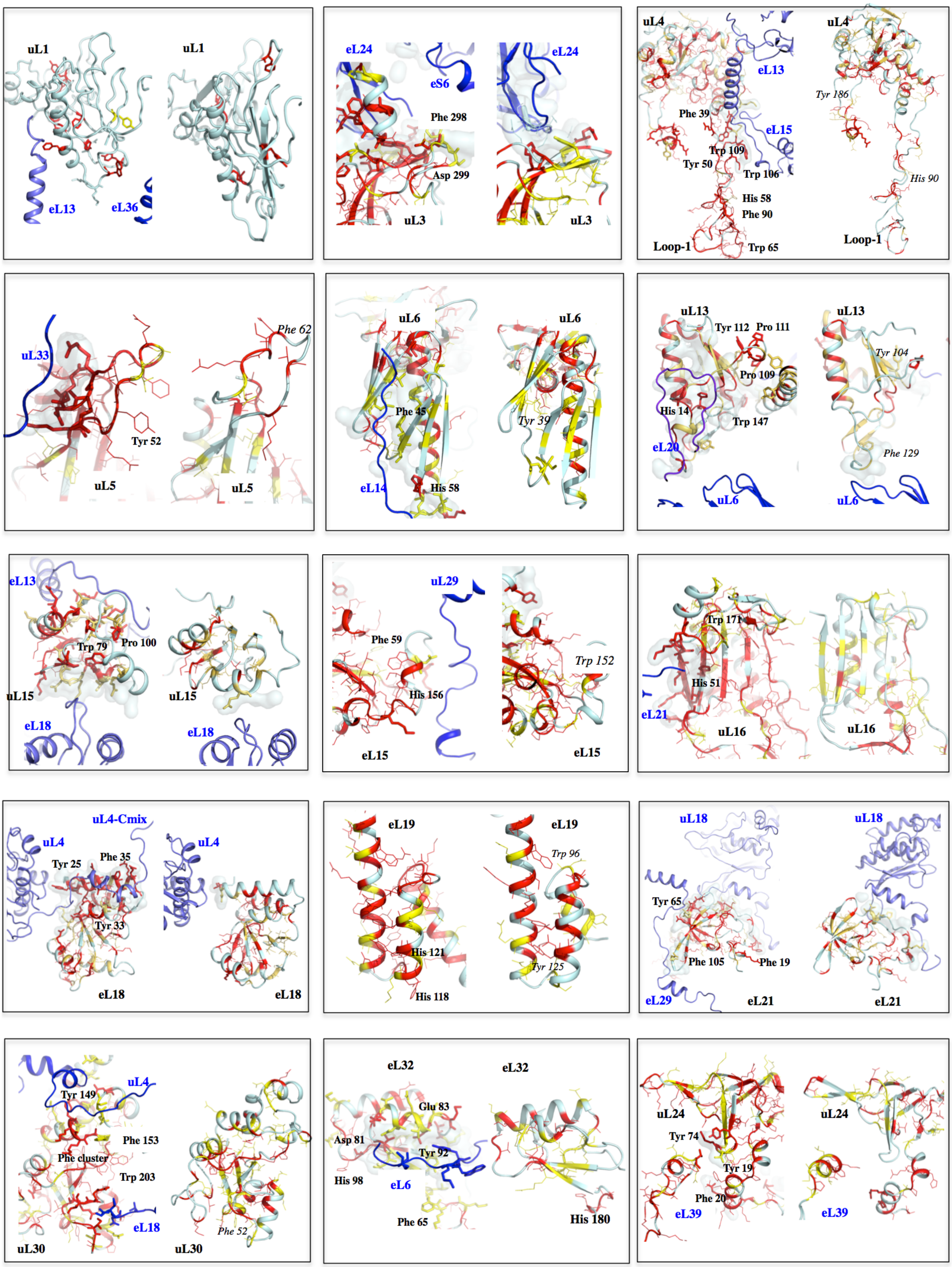
uL1
uL1
eL13
eL36
eL24
eS6
Phe 298
Asp 299
uL3
eL24
uL3
uL4
eL13
Phe 39
eL15
Trp 109
Tyr 50
Trp 106
His 58
Phe 90
Loop-1
Trp 65
uL4
Tyr 186
His 90
Loop-1
uL33
Phe 62
Tyr 52
uL5
uL5
uL6
Phe 45
eL14
His 58
uL6
Tyr 39
uL13
Tyr 112
Pro 111
Pro 109
His 14
Trp 147
eL20
uL6
uL13
Tyr 104
Phe 129
uL6
eL13
Trp 79
Pro 100
uL15
eL18
uL15
eL18
uL29
Phe 59
His 156
eL15
Trp 152
eL15
Trp 171
His 51
eL21
uL16
uL16
uL4-Cmix
uL4
Tyr 25
Phe 35
Tyr 33
eL18
uL4
eL18
eL19
His 121
His 118
eL19
Trp 96
Tyr 125
uL18
Tyr 65
Phe 105
Phe 19
eL29
eL21
uL18
eL21
uL4
Tyr 149
Phe 153
Phe cluster
Trp 203
eL18
uL30
Phe 52
uL30
eL32
Glu 83
Asp 81
Tyr 92
His 98
eL6
Phe 65
eL32
His 180
uL24
Tyr 74
Tyr 19
Phe 20
eL39
uL24
eL39

Figure 17

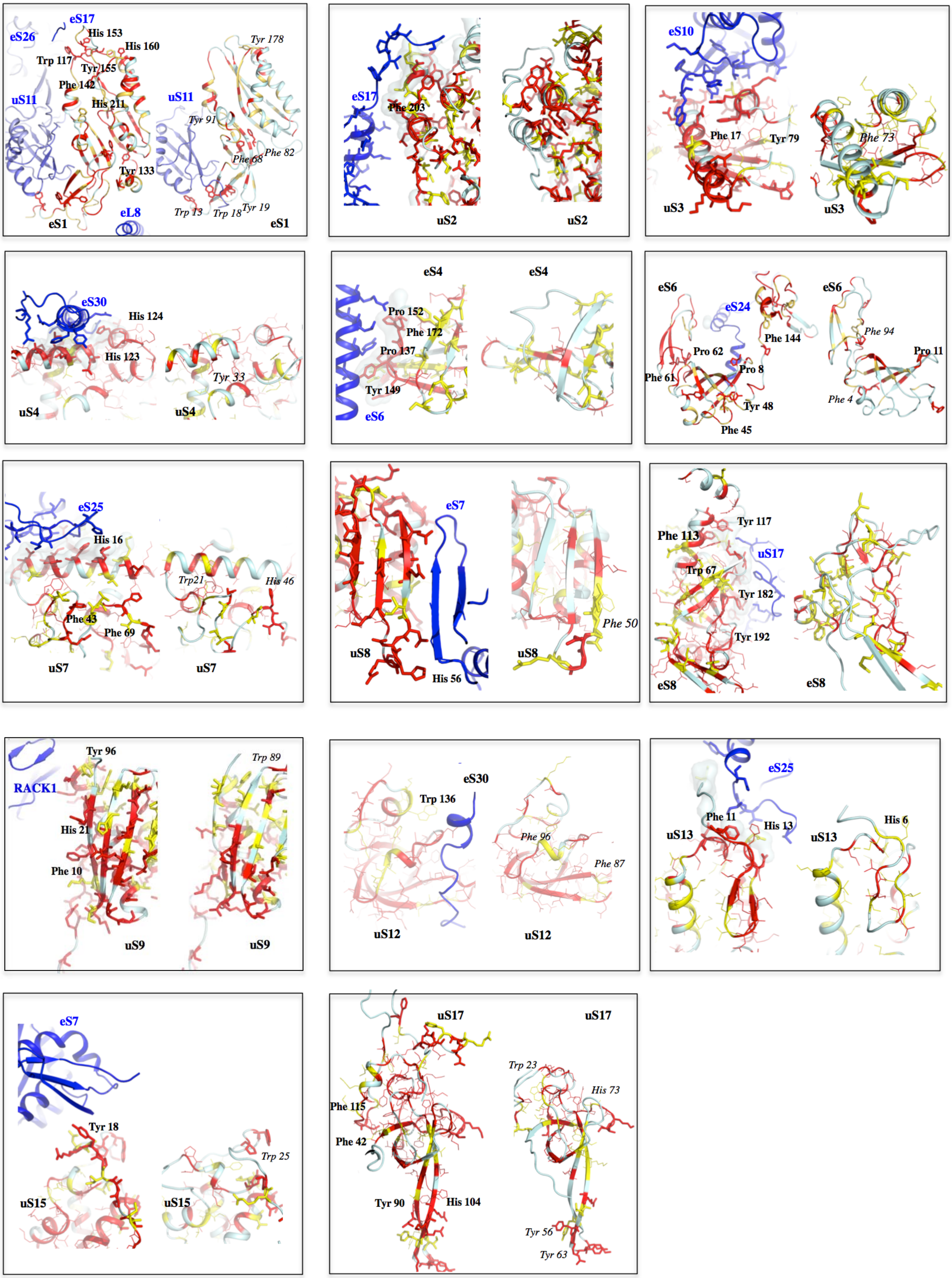

Figure 17 (continued)

Eukarya

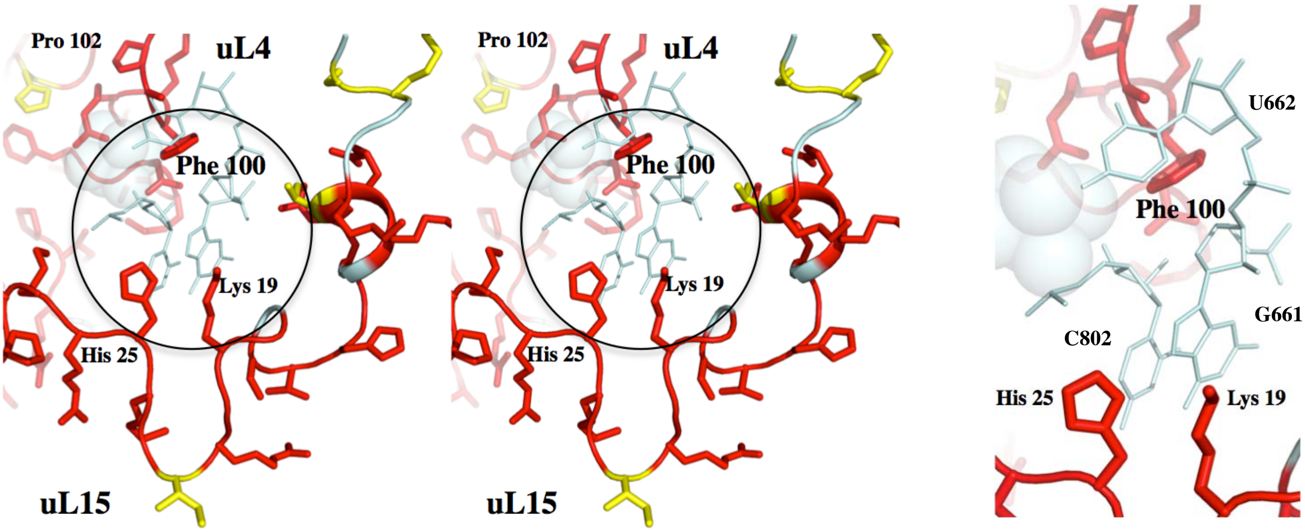

Archaea

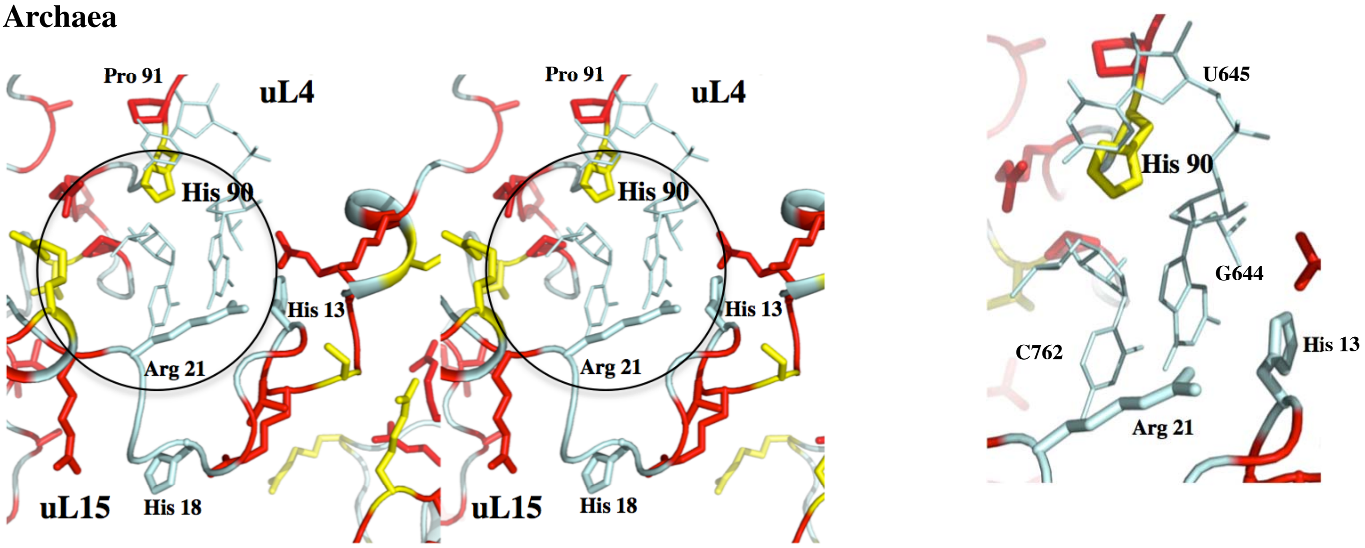

Bacteria

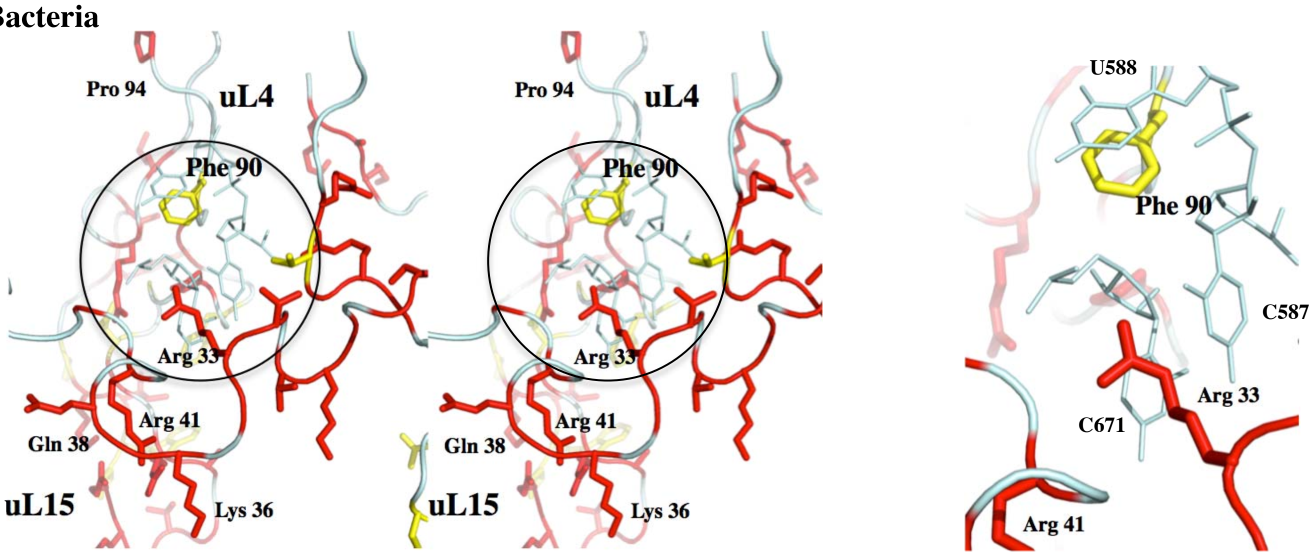

Figure 18

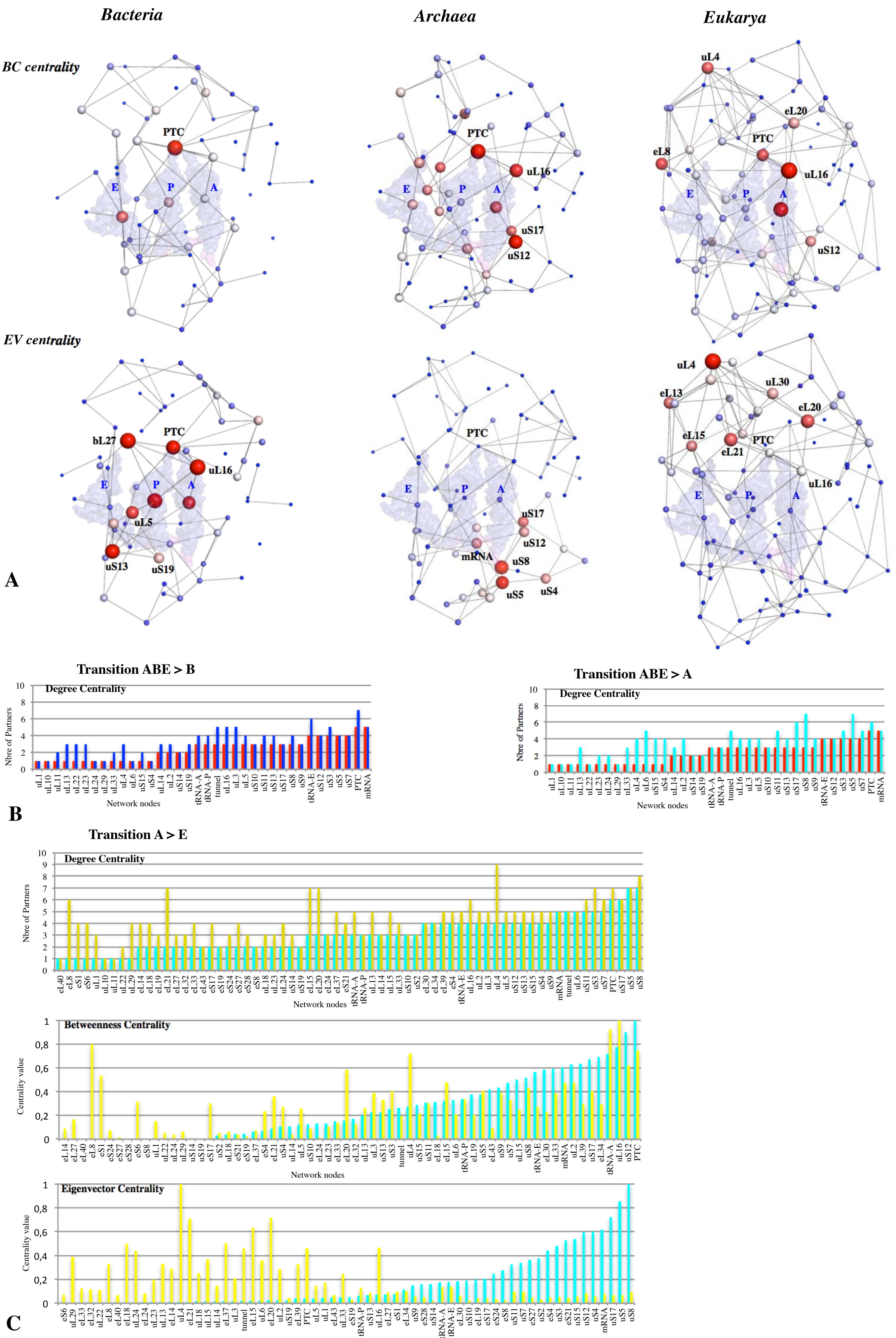
Bacteria
Archaea
Eukarya
BC centrality
EV centrality
PTC
E
P
A
uL16
uS17
uS12
uL4
eL20
eL8
bL27
uL5
uS13
uS19
mRNA
uS8
uS5
uS4
uL30
eL13
eL15
eL21
A
Transition ABE > B
Degree Centrality
Nbre of Partners
Network nodes
Transition ABE > A
Nework nodes
B
Transition A > E
Betweenness Centrality
Centrality value
Eigenvector Centrality
C
Figure 19
