# Supplementary material for: Evolution of ribosomal protein network architectures
Source: Sci Rep. 2021 Jan 12;11:625. doi: 10.1038/s41598-020-80194-4 (PMC7804294; doi:10.1038/s41598-020-80194-4)
Supplement: Supplementary file 4 — Supplementary Information 4. [file 41598_2020_80194_MOESM4_ESM.pdf]

Supplementary information

for

Evolution of ribosomal protein network architectures

Youri Timsit[1], Grégoire Sergeant-Perthuis[2] and Daniel Bennequin[2]

[1]Mediterranean Institute of OceanographyUM 110, Aix-Marseille Université, CNRS, IRD, Campus de Luminy, Marseille, France
[2]Institut de Mathématiques de Jussieu - Paris Rive Gauche (IMJ-PRG) UMR 7586 CNRS-Université Paris Diderot, Paris, France

Table Captions

Table 1: Number of r-proteins, extensions and their partners in the three kingdoms' networks
Summary of the r-proteins, extensions and connections in the ABE, B, A and E r-protein networks. Unconnected r-proteins are also included. Abbreviations for extensions evolutionary status Ub: extension acquired in bacteria on a universal r-protein; Ua: extension acquired in archaea on a universal r-protein; Ue: extension acquired in eukarya on a universal r-protein; Ae: extension acquired in eukarya on a archaeal protein.

Table 2: Evolutionary status of the r-proteins, their extensions and their partners in the network
The r-proteins and their extensions are represented according to their evolutionary status. ABE: red; B: blue; A: cyan; E: yellow. The columns 4-6 indicate the full size of the r-protein (number of amino acids) in the lines corresponding to their globular domains (boxes with grey background); the extension ranges (position of amino acids in the sequence) are indicated in front of the corresponding extensions (boxes with white background). R-proteins or extensions that occupy a similar position but in a different kingdom ribosome is indicated in parenthesis. "NV" means that the corresponding amino acids are not visible in the electron density maps. The reference PDB structures are: 4v88 for eukaryotes, 4v6u for archaea and 4y4p for bacteria (see supplementary table 10). A star "*" indicates that a r-protein is not present in all the eukaryote species.

Table 3: Three kingdoms' specific interactomes
List of the kingdom specific interactions between the r-protein components and/or the functional sites (Globular domains or extensions) coloured according to the evolutionary status of the interacting partners. ABE universal, Bacterial (B), Archaeal (A) and Eukaryotic (E) networks. The r-proteins that are not connected are not indicated. Bacterial total interactome is the sum of ABE+B; Archaeal total interacome is the sum of ABE+A; Eukaryotic total interactome is the sum of ABE+A+E interactions. The evolutionary patterns giving rise to co-evolutionary phenomena are also indicated.

Table 4: Multiple connections in the three kingdoms' networks
The r-protein pairs that have more than one connection are represented. Each r-protein is coloured according to its functional module: light blue: mRNA or tRNA functional modules; grey: PTC functional module; violet: tunnel functional module; light pink: subunit bridges; yellow: node that bridges two distinct functional modules; red: node that bridges three distinct functional modules (see Fig. 3 and Supplementary Fig. 11).

Table 5: Extension acquisitions at evolutionary transitions
a. Acquisition of new extensions in universal r-proteins
b. Acquisition of new extensions in archaeal proteins
c. and d. Bacterial and eukaryote specific proteins and their extensions

Table 6: Properties of coevolved extensions in eukaryotic ribosomes
The 4 first columns reports the coevolved partners, the 5th column reports the conserved amino acids of the extensions (**bold = strictly conserved**; normal = conservation by amino acid type (aromatic, acid, basic, polar, HP hydrophobic), the 6th column reports the rRNA ES bases that interact with the coevolved interfaces (the "*" indicates unstacked rRNA bases) and the last one describe the third "ancient" interacting partners. Coevolutions involving new conserved aromatic amino acids are coloured in orange. Note that since the coevolved extensions have been specifically acquired in the eukaryotic r-proteins, their conserved residues do not have equivalent in archaea.

Table 7: Evolution of connectivity, centrality, phylogenetic profiles and function of each r-protein nodes of the B, A and E network.
For each r-protein (1st column): (Ne): number of extensions, (Np): number of partners, (Nc): number of connection, (Bet): Betweenness centrality, (Eig): Eigenvector centrality, (size): protein size in aa, (cons): overall % of protein conservation (number of conserved amino acids/total number of amino acids), (N_"amino acid" or "character"= aromatic, acid, basic, polar, HP hydrophobic): number of strictly conserved "amino acid" or

“character”, (R_”amino acid” or “character”= aromatic, acid, basic, polar, HP hydrophobic): ratio of strictly conserved “amino acid” or “character”/protein size. The detailed number of strictly conserved amino acids is also reported. The last column reports functional property of the node.

Table 8: Randomness of the r-protein graphs.
P-Values for the betweenness, degree and closeness centralities in the universal (ABE), archaeal (A), eukaryotic (E) and bacterial (B) networks. N: number of trials in the statistical test.

Table 9: Largest distances between centre of mass of interconnected r-proteins
The r-proteins are coloured according to their functional status (see legend of Supplementary Fig. 4)

Table 10: Summary of high resolution X-ray and cryo-EM ribosome structures used in this work.

Figure Captions

Figure 1: Diversity of r-protein interactions
r-protein pairs specific of the eukaryotic network (pdbid: 4v88) are represented by cartoons

Figure 2: the universal network
The Universal network: the universal interacting pairs (observed in the bacterial (left) and eukaryotic (right) ribosome) of the SSU and LSU are represented. The 2D resulting network shows symbolically the interacting components between the r-proteins (globular domains (circles) or extensions (lines) and/or the functional sites. The legend of the line codes for extensions is indicated in the box. Thin lines indicate the interactions between extensions or between globular domains and extensions or between globular domains.

Figure 3: ABE, B, A and E networks.
Coloured thin lines symbolize an interaction between the r-protein extensions (the legend of the line codes for the extensions is indicated in the box). Lines between two circles symbolize an interaction between two globular domains. The colours of the lines follow the code for the evolutionary status adopted throughout the manuscript except for eukarya specific connection that are represented with black lines, for clarity. “N” or “C” indicate if the seg or mix are N-terminal or C-terminal extensions. NC indicates proteins without a globular domain (uS14, eL29, eS30, eL37 and eL39). Loop numbers correspond to that of Table 1. Functional sites (PTC, Tunnel, tRNAs and mRNA) are represented in light blue. In A and E networks, the names of bacterial proteins which, by convergence, occupy a position similar to that of E or A r-proteins, are shown in blue below the circles.

Figure 4: properties of the PPi interactions of the 3 kingdoms’ networks

A. Sorted interface areas of the interacting r-protein components (globular domains and/or extensions) calculated in 12 structures of the three kingdom ribosomes (Table 10)
B. Distribution of the interface areas of the three kingdom ribosomes, SSU and LSU
C. Average of interface areas distributed according their evolutionary status of each studied ribosome’s species
D. Averaged of interface areas of ABE, B, A and E networks
E. Averaged % of conservation of interfaces (left rectangle) and of the r-proteins (right rectangle) in B, A and E networks

Figure 5: conservation of the ribosomal PPi interfaces in the tree of life
Left: Mapping and distributions of the phylogenetic conservation of the interfaces in the three kingdoms’ r-protein networks. The r-proteins are depicted by transparent light blue cartoons. The tRNA sites are indicated with pink cartoons. The protein-protein interfaces are represented by surfaces coloured according to their percentage of amino acid conservation (a blue-white-yellow palette correspond a gradient from 0 to 100 % amino acid conservation of each interface). The distribution of the % of conservations is depicted by the histograms in front of the corresponding r-protein network (LSU or SSU), with the same colour scheme.
Right: Cartoon representations of the 3 kingdoms’ ribosomes showing the strictly conserved residues (red) and residues conserved by type (yellow) at the PPi and histograms reporting quantitatively the overall interface properties. **NresI/NresP**: the ratio of the total numbers of amino acids found in the protein-protein interfaces

versus the total numbers of amino acids (aas) of the r-proteins; **% strictC**: the percentage of strictly conserved amino acids at the interfaces (number of strictly conserved aas in the interface/ total number of aas at the interfaces); **% simC**: the percentage of similar amino acids (conservation by type: basic, acidic, aromatic, polar and HP) at the interfaces (number of similar aas in the interface/ total number of aas at the interfaces); **% consI**: %strict + %similar amino acids at the interfaces; **% consP**: %strict + %similar amino acids of the full r-proteins.

Figure 6: Phylogenetic conservation and properties of the interfaces of the bacterial network. Histograms showing for each interfaces (from bottom to top): the number of amino acids per interface (Nbr_res_I); the surface in Å^2 (Surf_I); the surface per amino acid (surf/res); the percentage of conservation of the protein containing the interface (% cons_P); the percentage of conservation of the interface (% cons_I); the ratio of conservation of the interface conservation versus the protein conservation (ratio cons_I/ cons_P).

Figure 7: Phylogenetic conservation and properties of the interfaces of the archaeal network. The legend is the same as in Figure 6.

Figure 8: Phylogenetic conservation and properties of the interfaces of the eukaryotic network. The legend is the same as in Figure 6.

Figure 9: Intra-kingdom variations in bacterial networks

A. Comparison of the interactions between uS4 and bS16 in *M. smegmatis* (PDB_id:5o61) (left) and *T. thermophilus* (PDB_id:4y4p) (right) ribosomes, in the context of their SSU rRNA.
B. Cartoon view of the interaction between uS4 and bS16 r-proteins in *M. smegmatis*. The C-mix segment specifically observed in *M. smegmatis* is represented in magenta. The changes in uS4 residues that have been identified to appear concomitantly with the new contacts are represented by coloured sticks and are annotated.
C. Comparison of the interactions between bL17 and uL3 r-proteins in *M. smegmatis* (PDB_id:5o61) (left) and *T. thermophilus* (PDB_id:4y4p) (right) ribosomes, in the context of their LSU rRNA. The N-seg segment of bL17 adopts a different conformation in *M. smegmatis* and turns back towards the bL17 globular domain instead of contacting uL3. In this new conformation, the N-seg encircles a extrahelical adenine (orange stick) that is absent in other known bacterial ribosomes.
D. Comparison of the structure of bL9 in *M. smegmatis* (left) and *T. thermophilus* (right) in the context of their LSU rRNA.
E. Comparison of the structures and interactions of uS14 and uS19 r-proteins of *E. coli* (PDB_id:4ybb) (left) and *T. thermophilus* (PDB_id:4y4p) (right). In *E. coli*, uS14 has an additional loop (magenta) that directly contacts uS19.
F. Close up of the uS14-uS19 interface in *E. coli* ribosome. The new uS19 and uS14 residues that have been identified to appear concomitantly with the new contact are represented with sticks and are annotated.

Figure 10: Intra-kingdom variations in eukaryotic networks.
A. Positions of the eukaryotic ribosomes analysed here, in the eukaryotic tree (adapted from Burki et al. 2020)
B. Variations of extensions and network connectivity in the eukaryotic LSU network. The colour of the variable interactions correspond to that of species represented in the tree in A
C. Comparison of uL4 C-mix connectivity in *yeast* (4v88), *plasmodium* (3j79) and *human* (4ug0) ribosomes.
D. Variations of eL6 extension sizes and connectivity in *yeas*t (4v88) and human (4ug0) ribosomes.
E. Variations of uL13, eL14 and eL33 extensions and connectivity in *leishmania* (5t2a) ribosomes. The specific extensions of eL14 and eL33 are coloured in magenta
F. Variations of uL22 extensions and connectivity in *yeast* ribosomes (4v88)

Figure 11: Functional representations of the r-protein networks.
A. Cartoon view of the bacterial ribosome (4y4p) in which the r-protein are coloured according to the functional site they connect: light blue: mRNA or tRNA functional modules; grey: PTC functional module; violet: tunnel

functional module; light pink: subunit bridges; yellow: node that bridges two distinct functional modules; red: node that bridges three distinct functional modules.
B. 2D and 3D structures of the Peptidyl Transfer Center adapted from Polacek & Mankin (2005)
C. A view of the nascent peptide in the peptide tunnel adapted from Timsit and Bennequin (2009)
D. Functional clusters of modules in the bacterial network
E. Functional clusters of modules in the archaeal network
F. Functional clusters of modules in the eukaryotic network

Figure 12: Evolution of contact types in r-protein networks.
Left: proportion of contact types in the interactions of the ABE, and the new set of interactions acquired in B, A and E networks. Hatched rectangles correspond to the interactions with functional sites.
Middle: for each network, top: pie charts reporting the proportion of contact types for the ribosome. Abbreviations: ext-ext: extension-extension; ext-G: extension-globular domain; ext-funct: extension-functional site; G-G: globular domain-globular domain; G-funct: globular domain-functional site. Bottom: pie chart reporting the proportions of the evolutionary status of the connections specifically acquired in the B, A and E networks. Abbreviations: U, A, B and E correspond to universal, archaeal, bacterial and eukaryotic r-proteins. Ub: Universal protein with an extension acquired in bacteria. Ua: Universal protein with an extension acquired in archaea. Ue: Universal protein with an extension acquired in eukarya. Ae: Archaeal protein with an extension acquired in eukarya.
Right: proportion of contact types for the SSU and LSU network of each kingdom.

Figure 13: the fate of r-protein extensions and interactions during network evolution.
A. Evolution of universal r-proteins. An example of the gradual acquisition of extensions in the universal r-protein uL4 whose extensions are coloured according to their evolutionary status. Histograms reporting the evolution of extensions (top) and partners (bottom) for the LSU (left) and SSU (right).
B. Evolution of archaeal r-proteins. An example of the gradual acquisition of extensions in the archaeal r-protein eL8 whose extensions are coloured according to their evolutionary status. Histograms reporting the evolution of extensions (top) and partners (bottom) for the LSU (left) and SSU (right) archaeal proteins in the A and E networks.
C. Evolution of the distribution of the number of partners per nodes in the ABE, B, A and E networks.
D. Evolution of the distribution of the number of connections per nodes in the ABE, B, A and E networks.
E. Average of the numbers of extensions (left rectangle), partners (middle rectangle) and connections (right rectangle) in the ABE, B, A and E networks.

Figure 14: evolution of the extension types and sizes
Left: Pie charts of the proportion of extension types calculated on the extension set specifically acquired in each network, in the ribosome (left), SSU (middle) and LSU (right). Bottom: histograms comparing the proportions of extension types in each network.
Right: Proportion and distribution of extension sizes in the ABE, B, A and E networks. The proportions have been calculated on 31 (ribosome) 19 (SSU) and 12 (LSU) extensions of the ABE network; 27 (ribosome), 7 (SSU) and 20 (LSU) extensions acquired in the bacterial network; 49 (ribosome), 21 (SSU) and 28 (LSU) extensions acquired in the archaeal network and 53 (ribosome), 15 (SSU) and 36 extensions acquired in the E network

Figure 15: evolution of the geometrical properties of the r-protein classical graphs
A. Average distances between the centres of mass of connected proteins in the four networks.
B. Sorted distances between the centres of mass of connected r-proteins in ABE, B, A and E graphs. The hatched rectangles correspond to connections with functional sites.
C. 3D representations of the ribosome, SSU and LSU of the suprimposed ABE, B, A and E networks, respectively. The spheres correspond to the centre of mass of the r-protein globular domains. The spheres and connections are coloured according to the evolutionary status of the connections (red: ABE; blue: B; cyan A and yellow: E). The transparent surfaces corresponding to the r-proteins and extensions are also coloured according to their evolutionary status. For clarity, the surfaces of bacterial r-proteins are not included.
D. Distribution of the distances between the centres of mass in the ribosome, SSU and LSU for the ABE, B, A and E graphs.

Figure 16: evolution of the r-protein extensions rRNA interactions

A. Evolution of the contributions of different r-protein components in rRNA-proteins interactions. Comparison of the proportion of rRNA nucleotides (nt)involved in interactions with globular domains, extensions and Protein-Protein interfaces (PPi) interactions in bacterial (4y4p), archaeal (4v6u) and eukaryal (3j7r) ribosomes. The structures of rRNA are represented with surface coloured according to their interactions with specific parts of r-proteins: (blue) globular domains; (pale yellow) extensions; (orange) protein resides involved in PPi interactions. The Pie charts display the proportions of rRNA involved with these three r-protein components and are coloured with corresponding codes.

B. (left) Comparison of the number of amino acids of different evolutionary statuses involved in the intermolecular contacts of the eukaryotic ribosomes (rRNA, PPi and RNA expansion segments). **(Middle)** Cartoon and surface representations of the eukaryotic ribosome showing the proportions of protein components coloured according to their evolutionary status that interact with rRNA. The Pie charts display the proportions of r-proteins of different evolutionary statuses, universal (red), archaeal (cyan) and eukaryotic (yellow) interacting with rRNA**. (Right)** rRNA that interacts with r-proteins are represented with coloured surfaces according the evolutionary status of the interacting r-proteins. The Pie charts display the proportions of rRNA interacting with universal (red), archaeal (cyan) and eukaryotic (yellow) r-proteins.

C. **(left)** The Pie charts represent the proportion of the universal, archaeal and eukaryotic r-protein components involved in interaction with Expansion Segments (ES) in the LSU and the SSU. **(Right)** Stereo view of the interactions r-proteins with rRNA Expansion Segments (ES) in the eukaryotic ribosome (yeast 4v88). The ES are represented in magenta. The ES that interact with r-proteins are represented with surfaces coloured according to the evolutionary status of the interacting r-proteins.

Figure 17: coevolutions at the amino acid level.

Comparison of the conserved amino acids in r-proteins common to archaea and eukarya in the eukaryotic ribosomes (left of the box) and the archaeal ribosomes (right of the box). Strictly conserved residues are depicted in red sticks. Amino acids conserved by type (aromatic, basic, acid, polar of hydrophobic) are represented by yellow sticks. Amino acid conserved and common in both archaea and eukarya are noted in *italic.* New conserved amino acids in eukarya are noted in **bold normal characters**. New interacting partners in eukaryotes are represented with blue cartoons.

Figure 18: the special case of uL15 - uL4 "distant" interaction"

Stereo views of the distant approach and conserved residues in the uL4-uL15 interactions in E,A and B ribosomes. Strictly conserved are represented by red stick and similar residues (aa type) are represented by yellow sticks.

Figure 19: Evolution of the node properties

A. Comparison of the node centrality (Betweenness (BC) and Eigenvector (EV)) in the B, A and E networks.
B. Changes of the degree centrality (nb of partners) in universal proteins during the ABE>B and ABE>A transitions.
C. Changes in degree, BC and EV centrality during the A->E transitions. Comparison of the values of centralities of each nodes of the archaeal and eukaryotic networks.

	**Proteins**			**Extensions**			**Connections**		
	**Ribosome**	**SSU**	**LSU**	**ribosome**	**SSU**	**LSU**	**ribosome**	**SSU**	**LSU**
**Universal**	34	15	19	31	19	12	49	29	20
**Bacteria**	18	4	14	27	7	20	51	12	39
Ub				8	5	3			
B				19	2	17			
**Archaea**	30	11	19	49	21	28	79	35	44
Ua				25	11	14			
A				24	10	14			
**Eukarya**	13	7	6	53	17	36	95	39	56
Ue				12	1	11			
Ae				28	9	19			
E				13	7	6			

Table 1

Table 2

Protein	Evolution Status		Eukarya	Archaea	Bacteria	Network interactions			
	Protein	Extension	Prot. size<br>Ext. range	Prot. size<br>Ext. range	Prot. size<br>Ext. range	ABE	AE	E	B
**uL1**	ABE	G	217	216	220	tRNA-E		eS25 (37jr)<br>eL13 (4ug0)	
	ABE	seg-N	1-12	1-10	1-4				
**uL2**	ABE	G	254	239	276		eL43		**bS6**
	ABE	mix-N	2-33	1-36	1-34		eL15		
	E	Loop-1	64-71	-	-			eL8	
	ABE	mix-C	177-253	167-239	199-276	uL2<br>PTC			
**uL3**	ABE	G	387	365	206	uL14	eL24	eS6	**bL19**
	A	seg-N	1-21	1-24	-		uL14<br>PTC		
	A	Loop-1	59-71	54-65			uL14		
	A	β-HP-2	84-106	78-100	31-51		uL13		
	ABE	loop-3	220-274	225-276	109-162	uL13<br>PTC			**bL17**
	A	Seg-C	340-367	341-365	-			eL24	
	E	Helix-C	367-387	-	-			eL24	
**uL4**	ABE	G	362	255	210		eL18		
	ABE	loop-1	44-115	41-107	44-103	Tunnel	eL37	eL15<br>eL13	**bL20**<br>**uL15**
	A	loop-2	183-202	174-195	-		uL24		
	E	mix-C	262-362	-	-			uL30<br>eL18<br>eL20<br>eL21	
**uL5**	ABE	G	174	186	182	uS13<br>uS19			**bL31**
	B	mix-N	-	-	2-28				**bL31**
	ABE	loop-1	48-66	60-76	72-88	tRNA-P		uL33	
	AE	loop-2	131-156	144-166	-		uL18		
**uL6**	ABE	G	191	184	180	PTC	uL13<br>eL14<br>eL20<br>eL40	eL14	
**eL6**	E	G	176	x	x			eL14<br>eL33	
	E	seg-N	2-30	x	x			eL32	
	E	seg-C	160-176	x	x			eL14<br>eL33	
**eL8**	A	G	256	123	x		eL15	eL36	
	E	mix-N	24-73	1-9	x<br>(bL9-bL28)			uL2<br>uL23<br>eL27	
	E	loop-1	97-131	-	x				
	E	mix-C	231-256	-	x			eS1 (4ug0)	
**bL9**	B		x (eL8)	x (eL8)	148				**bL28**
	B	Dom-1	x (eL8)	x (eL8)	1-41				**bL28**
	B	Hel-1	x	x	41-77				**bL28**
	B	Dom-2	x	x	77-146				
**uL10**	ABE	G	217	217	133	uL11			
**uL11**	ABE	G	134	134	137	uL10			**bL12**
**bL12**	B	G	x	x	122				**uL11**
**uL13**	ABE	G	199	142	140		uL3<br>eL33	eL20	**bL20**
	B	seg-N	-	-	1-12				**bL20**<br>**bL21**
	ABE	loop-1	58-76	54-68	71-86	uL3			
	A	loop-2	120-135	112-128	-		uL6		
	E	Helix-C	145-199	-	-			eL14	
**eL13**	E	G	199	x	x			uL29<br>eL36	
	E	mix-N	2-46	x	x			uL4<br>uL15<br>eL15<br>eL18	
	E	mix-C	151-194	x	x			uL1 (4ug0)<br>uL15	

Protein	Evolution Status		Eukarya	Archaea	Bacteria	Network interactions			
	Protein	Extension	Prot. size<br>Ext. range	Prot. size<br>Ext. range	Prot. size<br>Ext. range	ABE	AE	E	B
uL14	ABE	G	137	141	122	uL3<br>PTC	eL24		bL19
	A	Seg-N	2-17	10-20	-		uL3		
	A	loop-1	39-51	43-55	24-32		uL3		
eL14	A	G	138	83	x		uL6<br>eL20		
	E	seg-N	3-15	-	x			uL6<br>eL20	
	E	Helix-C	89-138	-	x			uL13<br>eL6	
uL15	ABE	G	149	146	150		eL18	eL13<br>eL36	
	ABE	mix-N	2-72	1-61	1-79		eL32<br>uL33	eL18<br>eL13	bL21<br>bL35<br>uL4
eL15	A	G	204	194	x		eL8	uL29<br>eL36	
	A	loop-1	67-98	67-96	x		uL2<br>uL33		
	A	seg-C	172-194	170-194	x				
	E	mix-C	194-204	-				eL13<br>uL4	
uL16	ABE	G	221	170	141	tRNA-A	eL20	eL21	bL25
	ABE	seg-N	2-35	3-39	1-31	tRNA-A<br>tRNA-P			bL25
	ABE	loop-1	99-121	94-119	76-89	tRNA-A<br>tRNA-P<br>PTC			bL27
	E	mix-C	199-221	-	-			uL18	
bL17	B	G	x (eL31)	x (eL31)	118				
	B	Seg-N	x	x	1-14				uL3
	B	Loop-1	x	x	99-111				uL22<br>bL32
uL18	ABE	G	297	197	112				bL27
	A	mix-N	1-30	1-20	-		uL5<br>eL21		
	E	mix-C	254-297	-	-			uL16	
bL19	B	G	(eL24)	(eL24)	146				uL3<br>uL14
	B	mix-N	x	x	1-14				uL3
	B	mix-C	x	x	116-135				
eL18	A	G	186	120	x		uL4<br>uL15	uL4	
	E	seg-N	2-20	-	(bL35-<br>uL15)			uL30	
	E	mix-C	144-186	-	x			uL15<br>eL13	
eL19	A	G	189	150	x		eL34		
	A	mix-C	53-150	53-150	x		uS17	eL43	
	E	hel-C	150-189	-	x			uS17<br>eS7	
bL20	B	G	(eL33)	(eL33)	118				uL13<br>bL21
	B	mix-N	(eL32)	(eL32)	1-72				uL4<br>uL13<br>bL21<br>bL32
eL20	A	G	172	77 (LX)	x (bL25)		uL16<br>eL14		
	E	Dom-1	1-70	-	x			uL4<br>uL30	
	E	loop-1	11-25	-	x			eL21	
	A	mix-C	128-145	60-77	x		uL6		
	E	seg-C	145-172	-	x			uL13<br>eL14	

Protein	Evolution Status		Eukarya	Archaea	Bacteria	Network interactions			
	Protein	Extension	Prot. size<br>Ext. range	Prot. size<br>Ext. range	Prot. size<br>Ext. range	ABE	AE	E	B
bL21	B	G	x	x	101				uL13<br>bL20
	B	β-HP	(eL32)	(eL32)	70-87				uL15<br>bL20
eL21*	A	G	160	97	x (bL27)			eL29	
	A	seg-N	2-27	1-28	x		uL18<br>PTC		
	E	mix-C	98-160	-	x			uL4<br>uL16<br>uL30<br>eL20	
uL22	ABE	G	184	155	113				bL17<br>bL32
	A	Loop-1	53-84	57-88					
	ABE	β-HP	122-143	125-141	78-101	tunnel			
	E	mix-C	152-184	-	-			eL33	
eL22	E	G	121	x	x				
uL23	ABE	G	142	86	96	uL29			
	E	seg-N	22-59	-	-			eL8	
	B	β-HP-1	-	-	59-76<br>(eL39)				Tunnel<br>bL34
	A	loop-1	114-120	56-62	-		eL39		
uL24	ABE	G	127	121	110			eL39<br>eL37	
	A	mix-N	2-24	1-23	-		uL4		
	ABE	β-HP	83-100	80-94	44-64	tunnel			
bL25	B	G	x (eL20)	x (LX)	206				uL16
	B	mix-C	x	x	177-202				
eL24	A	G	155	66	x (bl19)		uL3<br>uL14<br>eS6		
	E	mix-C	59-98	-	x			eS6<br>uL3	
bL27*	B	G	(eL21)	(eL21)	85				uL18
	B	Seg-N	x	x	2-28				uL16<br>tRNA-A<br>tRNA-P<br>PTC
eL27	A	G	136	83 (eL14)	x		eL30<br>eL34		
	E	loop-1	48-69	-	x			eL8	
bL28	B	G	x	x	98				bL9
	B	β-HP	x	x	13-42				tRNA-E
uL29	ABE	G	120	72	72	uL23			
	E	mix-C	70-120	-	-			uL23<br>eL15<br>eL37<br>eL13	
eL29	E	G	59	x	x				
	E	mix-NC	2-59	x	x			eL21<br>PTC	
uL30	ABE	G	244	155	60			eL18	
	E	mix-N	23-83	-	-			uL4<br>eL20<br>eL21	
eL30	A	G	105	99	x		uS15<br>eL27<br>eL34<br>eL43		
eL31	A	G	113	95	x				
bL31	B	G	x	x	71				uL5<br>uS13
	B	seg-N	x	x	1-9				
	B	seg-C	x	x	37-69				uS13<br>uS19
bL32	B	G	x	x	60				uL22<br>bL17
	B	mix-N	x	x	2-31				uL22<br>PTC<br>bL20

Protein	Evolution Status		Eukarya	Archaea	Bacteria	Network interactions			
	**Protein**	**Extension**	**Prot. size**<br>**Ext. range**	**Prot. size**<br>**Ext. range**	**Prot. size**<br>**Ext. range**	**ABE**	**AE**	**E**	**B**
**eL32**	A	G	130	129	x			eL6	
	A	loop-1	2-72	1-80	x (ext of bL20-bL21)		uL15<br>eL33		
**uL33**	ABE	G	106	94	54	tRNA-E			**bL35**
	A	Loop-1	27-69	26-66	-		uL15<br>tRNA-E<br>eL15		
	E	Seg-C	95-106	-	-			uL5	
**eL33**	A	G	107	87	x (bL20-G)		uL13		
	E	seg-N	2-8	-	x			eL6	
	A	loop-1	16-28	10-22	x		eL32		
	E	loop-2	54-64	-	x			uL22	
	E	seg-C	101-107	-	x			eL6	
**eL34**	A	G	121	89	x		eL27		
	A	seg-N	2-58	1-52	x		eL39<br>eL19*		
	A	Hel-C	80-113	73-89	x		eL27<br>eL30		
**bL34**	B	G	x (eL37)	x (eL37)	49				**uL23**
	B	mix-N	x	x	1-9				**Tunnel***
**bL35**	B	G	x	x	65				**uL15**
	B	Loop-1	x	x	25-46				**tRNA-E**<br>**uL33**
**bL36**	B		x (eL40)	x (eL40)	37				
**eL36**	E	G	100	x	x			eL8<br>eL15	
	E	mix-N	2-49	x	x			uL15<br>eL13	
**eL37**	A	mix-NC	88	62	x				
	A	mix-NC	2-62	1-62	x (bL34)		Tunnel<br>uL4<br>eL39		
	E	mix-C	62-88	-	x			uL24<br>uL29	
**eL38***	E	G	78	x	x				
**eL39**	A		51	51	x				
	A	mix-NC	2-51	1-51	x		Tunnel<br>uL23<br>eL37<br>eL34	uL24	
**eL40**	A	G	128	51	x (bL36)		uL6		
	AE	mix-N	1-77 NV	-	x		uL6		
**eL41**	A	G	25	25	x				
**eL43**	A	G	92	83	x		uL2<br>eL30		
	A	mix-N	2-35	6-35	x			eL19*	
	A	mix-C	65-92	64-83	x		uL2		

Protein	Evolution status		Euk	Archaea	Bact	Network interactions			
	protein	extension	Prot. size<br>Ext. range	Prot. size<br>Ext. range	Prot. size<br>Ext. range	ABE	A	E	B
**eS1**	A	G	255	198	x		uS11	eL8(4ug0)<br>eS26	
	A	seg-N	20-29	9-16	(bS6+bS18)		uS11		
	E	loop-1	144-154	-	x			eS17	
	A	seg-C	233-254	192-198	x				
**uS2**	ABE	G	252	202	256		uS5<br>eS17<br>eS21		
	B	Dom-1	(99-120)	(90-114)	94-161				uS3 ?
	ABE	seg-C	207-252 NV	195-202	237-256 NV		eS17		
**uS3**	ABE	G	240	210	239	mRNA<br>uS5<br>uS10<br>uS14		eS10	
	A	hel-N*	3-16	1-16	-		uS10<br>uS14		
	B	mix-N*	-	-	2-29				uS10<br>uS14
	ABE	seg-C	189-225<br>>225 **NV**	183-186<br>>210 **NV**	203-207<br>>239 **NV**		eS17	RACK1	uS2 ?
**uS4**	ABE	G	197	180	209	uS5		eS30	
	A	seg-N*	2-20	9-20<br>(1-9 NV)	-		eS4		
	B	Dom-1	(eS30)	(eS30)	2-53				
	A	Dom-1	51-99	51-96			uS5<br>uS8	eS4	
	B	Dom-2	-	-	148-209				uS5
	A	Mix-C	156-186	153-180	-		eS24		
**eS4**	A	G	261	243	(bS16)		eS24	eS6	
	A	seg-N	2-37	1-44			uS4<br>eS8		
	A	Loop-1	197-211	201-212	x		uS17		
	E	mix-C	242-261	-	x			uS4	
**uS5**	ABE	G	254	236	162	uS3<br>uS4<br>uS8			
	A	Dom-1	34-74<br>1-33 NV	1-60	-		uS2<br>eS21		
	ABE	β-HP	80-105	62-91	7-36	mRNA			
	A	loop-1	141-153	126-139	-		eS21		
	ABE	mix-C	225-250	211-217<br>(>236 NV)	141-152<br>(>162 NV)		uS8<br>eS21		uS8
**bS6**	B	G	(es1)	(eS1)	101				uS15<br>uL2<br>bS18
	B	seg-C	x	x	92-100				bS18
**eS6**	A	G	236	125	x		eL24	uL3<br>eS24	
	E	mix-C	118-226	-	x			eS4	
**uS7**	ABE	G	225	215	156	uS9<br>uS11	eS28	eS25	
	ABE *	mix-N	20-81	1-63	2-21*	uS9	eS19		
	ABE	loop-1	147-158	137-148	78-85	mRNA<br>tRNA-E			
**eS7**	E	G	190	x	x			uS8<br>uS15<br>eS27<br>eL19	
	E	loop-1	94-113	x	x				
**uS8**	ABE	G	130	130	138	uS5<br>uS12<br>uS17	uS4<br>uS15<br>eS21<br>eS27	eS7	
	B (thermus)	loop-1	-	-	63-82				uS2
**eS8**	A	G	200	127	(bS20)		eS4	uS17	
	A	loop-1	2-29	1-29	bS20		uS17		
	E	Dom-1	103-200	-	x			uS17	
**Protein**	**Evolution status**		**Euk**	**Archaea**	**Bact**	**Network interactions**			

	protein	extension	Prot. size<br>Ext. range	Prot. size<br>Ext. range	Prot. size<br>Ext. range	ABE	A	E	B
**uS9**	ABE	G	143	135	128	uS7	eS19	RACK1	
	ABE	seg-C	113-143	102-135	97-126	tRNA-P<br>uS10			
**uS10**	ABE	G	121	102	105	uS3			
	ABE	β-HP	60-87	43-68	43-67	uS3<br>uS9<br>uS14			
**eS10**	E	G	105	x	x			uS3 uS14<br>eS12	
	E	seg-C	80-96	x	x			uS3	
**uS11**	ABE	G	137	137	129	tRNA-E<br>uS7	eS1<br>eS28	eS26	bS18
	ABE	seg-N	11-14<br>1-13 NV	5-13	13-16<br>1-12 NV				
	A	loop-1	84-95	84-96	-	mRNA		eS26	
	ABE	seg-C	117-137	117-137	110-126	mRNA		eS26	
**uS12**	ABE	G	145	147	132			eS30	
	ABE	mix-N	2-48	3-48	5-25	uS8<br>uS17			
	ABE	loop-1	57-71	61-69	43-53	mRNA<br>tRNA-A			
**eS12**	E	G	143	uL30 (5jbh)<br>eL8 (4v6u)	x			eS31<br>eS10	
	E	loop-1	101-121		x			eS10	
**uS13**	ABE	G	146	148	126	uL5	eS19		bL31
	ABE	seg-N*	2-31	1-21	1-15			eS25	
	ABE	mix-C	102-146	93-148	94-124	tRNA-A<br>uS19			tRNA-P
**uS14**	ABE	G	56	56	61				
	ABE	mix-NC	4-56	1-56	2-61	uS10<br>uS3		eS10	
**uS15**	ABE	G	151	158	89	uS17	eL30		uS17#<br>bS6
	A	seg-N	2-28	1-30	-		uS8<br>eS27	eS7	
	A	Dom-1	29-70	31-77	-		eS27		
	A	mix-C	132-151	139-158	-		uS17		
**bS16**	B	G	x	x	88				
**uS17**	ABE	G	156	113	105		eS4<br>eS8<br>eL19		uS15#
	E	seg-N<br>(loops 1+2)	2-33	-	-			eS8<br>eL19	
	A	loop-3	34-45	1-30	-		eS4		
	ABE	β-HP	83-110	46-73	18-45	uS12<br>uS8			
	ABE	mix-C	142-156	106-113	79-100	uS15		eL19	
**eS17**	A	G	136	67	x		uS3<br>uS2	RACK1	
	E	mix-C	71-129<br>(37jr)	-	Ext uS2			uS2<br>eS1	
**bS18**	B	G	X (eS1)	X (eS1)	88				bS6
	B	Seg-C	x	x	77-87				uS11<br>bS6
**uS19**	ABE	G	142	132	93	uS13			bL31
	ABE	seg-N	8-21	6-19	2-14	uL5			
	ABE	seg-C**	120-131<br>>142 NV	107-116<br>>132 NV	75-84<br>>93 NV				
**bS20**	B	G	x	x	106				
**eS19**	A	G	144	150	x		uS9<br>uS13		
	A	β-HP	83-93	76-94	x				
**eS21 (sX)**	A	G	87	57 (sX)	x		uS2<br>uS8		
	A	seg-N	1-31	1-20	x		uS8<br>uS5<br>uS2		
	E	loop-1	37-50	-				uS2	
	E	seg-C	75-87	-	x			uS2<br>eS2	
**Protein**	**Evolution status**		**Euk**	**Archaea**	**Bact**	**Network interactions**			

	protein	extension	Prot. size<br>Ext. range	Prot. size<br>Ext. range	Prot. size<br>Ext. range	ABE	A	E	B
**eS24**	AE	G	135	99	x		uS4<br>eS4		
	E >>A	mix-C	95-135	90-99	x			eS6	
**eS25**	E	G	108	x	x			uL1<br>uS13<br>uS7<br>tRNA-E	
**eS26**	E	G	119	x	x			uS11<br>eS28	
	E	seg-N	2-20	x	x				
	E	loop-1	42-67	x	x			uS11<br>eS1	
	E	seg-C	81-98	x	x				
**eS27**	A	G	82	63	x		uS15		
	E >> A	mix-N	2-32	1-13	x		uS8 uS15	eS21<br>eS7	
**eS28**	A	G	67	71	x		uS7		
	A	Seg-N	5-8	1-7	x		uS11		
	A	Seg-C	56-67	57-71	x		uS11	eS26	
**eS30**	E		63	x	x				
	E	mix-NC	2-61	x	x			tRNA-A<br>uS4 uS12	
**eS31**	A	G	152	50	x			eS12	
	E	seg-N	82-107<br>1-82 NV	?	x			tRNA-A	
**RACK1**	E	G	319	x	x		uS3<br>uS9<br>eS17		

Table 3: ABE universal network.

Protein	part	status	Node	part	status
uS3	G	ABE	mRNA		ABE
	G	ABE	uS5	G	ABE
	G	ABE	uS10	G	ABE
	G	ABE	uS10	b-HP	ABE
	G	ABE	uS14	mix-NC	ABE
uS4	G	ABE	uS5	G	ABE
uS5	G	ABE	uS3	G	ABE
	G	ABE	uS4	G	ABE
	G	ABE	uS8	G	ABE
	b-HP	ABE	mRNA		ABE
	mix-C	ABE	uS8	G	ABE
uS7	G	ABE	uS9	G	ABE
	G	ABE	uS11	G	ABE
	mix-N	ABE	uS9	G	ABE
	loop-1	ABE	mRNA		ABE
	loop-1	ABE	tRNA-E	AC-stem	ABE
uS8	G	ABE	uS5	G	ABE
	G	ABE	uS5	mix-C	ABE
	G	ABE	uS12	mix-N	ABE
	G	ABE	uS17	b-HP	ABE
uS9	G	ABE	uS7	G	ABE
	G	ABE	uS7	mix-N	ABE
	seg-C	ABE	tRNA-P	AC-stem	ABE
	seg-C	ABE	uS10	b-HP	ABE
uS10	G	ABE	uS3	G	ABE
	b-HP	ABE	uS3	G	ABE
	b-HP	ABE	uS9	seg-C	ABE
	b-HP	ABE	uS14	mix-NC	ABE
uS11	G	ABE	tRNA-E	AC-stem	ABE
	G	ABE	uS7	G	ABE
	seg-C	ABE	mRNA		ABE
uS12	mix-N	ABE	uS8	G	ABE
	mix-N	ABE	uS17	b-HP	ABE
	loop-1	ABE	mRNA		ABE
	loop-1	ABE	tRNA-A	AC-stem	ABE
uS13	G	ABE	uL5	G	ABE
	mix-C	ABE	tRNA-A	AC-stem	ABE
	mix-C	ABE	uS19	G	ABE
uS14	mix-NC	ABE	uS10	b-HP	ABE
	mix-NC	ABE	uS3	G	ABE
uS15	G	ABE	uS17	mix-C	ABE
uS17	b-HP	ABE	uS8	G	ABE
	b-HP	ABE	uS12	mix-N	ABE
	mix-C	ABE	uS15	G	ABE
uS19	G	ABE	uS13	mix-C	ABE
	seg-N	ABE	uL5	G	ABE

Protein	part	status	Node	part	status
uL1	G	ABE	tRNA-E	CCA-stem	ABE
uL2	mix-N	ABE	uL2	mix-C	ABE
	mix-C	ABE	uL2	mix-N	ABE
	mix-C	ABE	PTC		ABE
uL3	G	ABE	uL14	G	ABE
	loop-3	ABE	uL13	loop-1	ABE
	loop-3	ABE	PTC		ABE
uL4	loop-1	ABE	tunnel		ABE
uL5	G	ABE	uS13	G	ABE
	G	ABE	uS19	seg-N	ABE
	loop-1	ABE	tRNA-P	CCA-stem	ABE
uL6	G	ABE	PTC		ABE
uL10	G	ABE	uL11	G	ABE
uL11	G	ABE	uL10	G	ABE
uL13	loop-1	ABE	uL3	loop-3	ABE
uL14	G	ABE	uL3	G	ABE
	G	ABE	PTC		ABE
uL15	mix-N	ABE	uL4	loop-1	ABE
uL16	G	ABE	tRNA-A	CCA-stem	ABE
	seg-N	ABE	tRNA-A	CCA-stem	ABE
	seg-N	ABE	tRNA-P	CCA-stem	ABE
	loop-1	ABE	tRNA-P	CCA-stem	ABE
	loop-1	ABE	PTC		
uL22	b-HP	ABE	tunnel		ABE
uL23	G	ABE	uL29	G	ABE
uL24	b-HP	ABE	tunnel		ABE
uL29	G	ABE	uL23	G	ABE
uL33	G	ABE	tRNA-E	CCA-stem	ABE

Table 3 (continued): interactions acquired in the bacterial network

Protein	part	status	Node	part	status
uS2	G	ABE	uS8	Loop-1	B
	dom-1	B	uS3	seg-C	ABE
uS3	mix-N	B	uS10	b-HP	ABE
	mix-N	B	uS14	mix-NC	ABE
	seg-C	ABE	uS2	dom-1	B
uS4	dom-2	B	uS5	G	ABE
uS5	G	ABE	uS4	dom-2	B
uS8	loop-1	B	uS2	G	ABE
uS10	b-HP	ABE	uS3	mix-N	B
uS11	G	ABE	bS18	seg-C	B
uS13	G	ABE	bL31	G	B
	G	ABE	bL31	seg-C	B
	mix-C	ABE	tRNA-P	AC-stem	ABE
uS14	mix-NC	ABE	uS3	mix-N	B
	G	ABE	bS6	G	B
uS19	G	ABE	bL31	seg-C	B

bS6	G	B	uS15	G	ABE
	G	B	uL2	G	ABE
	G	B	bS18	G	B
	seg-C	B	bS18	G	B
bS18	G	B	bS6	G	B
	G	B	bS6	seg-C	B
	seg-C	B	uS11	G	ABE

Protein	part	status	Node	part	status
uL2	G	ABE	bS6	G	B
uL3	G	ABE	bL19	G	B
	G	ABE	bL19	mix-N	B
	loop-3	ABE	bL17	seg-N	B
uL4	loop-1	ABE	bL20	mix-N	B
uL5	G	ABE	bL31	G	B
	G	ABE	bL31	seg-N	B
	mix-N	B	bL31	G	B
uL11	G	ABE	bL12	G	B
uL13	G	ABE	bL20	mix-N	B
	seg-N	B	bL20	G	B
	seg-N	B	bL21	G	B
uL14	G	ABE	bL19	G	B
uL15	mix-N	ABE	bL21	b-HP	B
	mix-N	ABE	bL35	G	B
	mix-N	ABE	uL4	loop-1	ABE
uL16	G	ABE	bL25	G	B
	seg-N	ABE	bL25	G	B
	loop-1	ABE	bL27	seg-N	B
uL18	G	ABE	bL27	G	B
uL22	G	ABE	bL17	loop-1	B
	G	ABE	bL32	G	B
	G	ABE	bL32	mix-N	B
uL23	b-HP	B	tunnel	tunnel	ABE
	b-HP	B	bL34	G	B
uL33	G	ABE	bL35	loop-1	B

bL9	dom-1	B	bL28	G	B
	hel-1	B	bL28	G	B
bL12	G	B	uL11	G	ABE
bL17	seg-N	B	uL3	loop-3	ABE
	loop-1	B	uL22	G	ABE
	loop-1	B	bL32	G	B
bL19	G	B	uL3	G	ABE
	G	B	uL14	G	ABE
	mix-N	B	uL3	G	ABE
bL20	G	B	uL13	seg-N	B
	G	B	bL21	G	B
	mix-N	B	uL4	loop-1	ABE
	mix-N	B	uL13	G	ABE
	mix-N	B	bL21	b-HP	B
	mix-N	B	bL32	mix-N	B
bL21	G	B	uL13	seg-N	B
	G	B	bL20	G	B
	b-HP	B	uL15	mix-N	ABE
	b-HP	B	bL20	mix-N	B
bL25	G	B	uL16	G	ABE
	G	B	uL16	seg-N	ABE
bL27	G	B	uL18	G	ABE
	seg-N	B	uL16	loop-1	ABE
	seg-N	B	tRNA-A	CCA-stem	ABE
	seg-N	B	tRNA-P	CCA-stem	ABE
	seg-N	B	PTC		ABE

bL28	G	B	bL9	dom-1	B
	G	B	bL9	hel-1	B
	b-HP	B	tRNA-E	CCA	ABE
bL31	G	B	uL5	G	ABE
	G	B	uL5	mix-N	B
	G	B	uS13	G	ABE
	seg-N	B	uL5	G	ABE
	seg-C	B	uS13	G	ABE
	seg-C	B	uS19	G	ABE
bL32	G	B	uL22	G	ABE
	G	B	bL17	loop-1	B
	mix-N	B	uL22	G	ABE
	mix-N	B	bL20	mix-N	B
	mix-N	B	PTC		ABE
bL34	G	B	uL23	b-HP	B
	mix-N	B	tunnel		ABE
bL35	G	B	uL15	mix-N	ABE
	loop-1	B	tRNA-E	CCA-stem	ABE
	loop-1	B	uL33	G	ABE

Coevolutions in Bacteria

Coevolution of extensions

Protein (ABE)	ext	B	Protein(ABE)	ext	B

Target of new contact

Protein(ABE)	G/ext	ABE	Protein(ABE)	ext	B
Protein(ABE)	G/ext	ABE	Protein(B)	G/ext	B

Donnor of new contact

Protein(ABE)	ext	B	Protein(ABE)	G/ext	ABE
Protein(B)	G/ext	B	Protein(ABE)	G/ext	ABE

Coevolutions in Archaea

Coevolution of extensions

Protein (ABE)	ext	A	Protein (ABE)	ext	A

Target of new contact (T)

Protein (ABE)	G/ext	ABE	Protein (ABE)	ext	A
Protein (ABE)	G/ext	ABE	Protein (A)	G/ext	A

Donnor of new contact (D)

Protein (ABE)	ext	A	Protein (ABE)	G/ext	ABE
Protein (A)	G/ext	A	Protein (ABE)	G/ext	ABE

Table 3 (continued): interactions acquired in the archaeal network

Protein	part	status	Node	part	status
uS2	G	ABE	uS5	dom-1	A
	G	ABE	eS21	G	A
	seg-C	ABE	eS17	G	A
uS3	hel-N	A	uS10	b-HP	ABE
	hel-N	A	uS14	mix-NC	ABE
	seg-C	ABE	eS17	G	A
uS4	seg-N	A	eS4	seg-N	A
	dom-1	A	uS5	G	ABE
	dom-1	A	uS8	G	ABE
	mix-C	A	eS24	G	A
uS5	G	ABE	eS21	seg-N	A
	G	ABE	uS4	dom-1	A
	dom-1	A	uS2	G	ABE
	dom-1	A	uS5	mix-C	ABE
	dom-1	A	eS21	G	A
	loop-1	A	eS21	seg-N	A
	mix-C	ABE	uS5	dom-1	A
	mix-C	ABE	eS21	G	A
uS7	G	ABE	eS28	G	A
uS8	G	ABE	uS15	seg-N	A
	G	ABE	uS4	dom-1	A
	G	ABE	eS21	G	A
	G	ABE	eS21	seg-N	A
	G	ABE	eS27	mix-N	A
uS9	G	ABE	eS19	G	A
uS10	G	ABE	uS3	hel-N	A
uS11	G	ABE	eS1	seg-N	A
	G	ABE	eS28	seg-N	A
	G	ABE	eS28	seg-C	A
	G	ABE	eS1	G	A
	loop-1	A	mRNA		ABE
uS13	G	ABE	eS19	G	A
uS14	mix-NC	ABE	uS3	hel-N	A
uS15	G	ABE	eL30	G	A
	seg-N	A	uS8	G	ABE
	seg-N	A	eS27	mix-N	A
	dom-1	A	eS27	G	A
uS17	G	ABE	eS4	loop-1	A
	G	ABE	eS8	loop-1	A
	G	ABE	eL19	mix-C	A
	loop-3	A	eS4	loop-1	A

eS1	G	A	uS11	G	ABE
	seg-N	A	uS11	G	ABE
eS4	G	A	eS24	G	A
	seg-N	A	uS4	seg-N	A
	seg-N	A	eS8	loop-1	A
	loop-1	A	uS17	G	ABE
	loop-1	A	uS17	loop-3	A
eS6	G	A	eL24	G	A
eS8	loop-1	A	eS4	seg-N	A
	loop-1	A	uS17	G	ABE
eS17	G	A	uS3	seg-C	ABE
	G	A	uS2	seg-C	ABE
eS19	G	A	uS9	G	ABE
	G	A	uS13	G	ABE
eS21	G	A	uS2	G	ABE
	G	A	uS8	G	ABE
	G	A	uS5	dom-1	A
	G	A	uS5	mix-C	ABE
	seg-N	A	uS5	G	ABE
	seg-N	A	uS5	loop-1	A
	seg-N	A	uS8	G	ABE
eS24	G	A	uS4	mix-C	A
	G	A	eS4	G	A
eS27	G	A	uS15	dom-1	A
	mix-N	A	uS8	G	ABE
	mix-N	A	uS15	seg-N	A
eS28	G	A	uS7	G	ABE
	seg-N	A	uS11	G	ABE
	seg-C	A	uS11	G	ABE

Protein	part	status	Node	part	status
uL2	G	ABE	eL43	G	A
	G	ABE	eL43	mix-C	A
	mix-N	ABE	eL15	loop-1	A
	mix-C	ABE	eL43	mix-N	A
uL3	G	ABE	eL24	G	A
	seg-N	A	uL14	loop-1	A
	seg-N	A	PTC		ABE
	loop-1	A	uL14	seg-N	A
	b-HP-2	A	uL13	G	ABE
uL4	G	ABE	eL18	G	A
	loop-1	ABE	eL37	mix-NC	A
	loop-2	A	uL24	mix-N	A
uL5	loop-2	A	uL18	mix-N	A
uL6	G	ABE	eL14	G	A
	G	ABE	uL13	loop-2	A
	G	ABE	eL20	mix-C	A
	G	ABE	eL40	G	A
uL13	G	ABE	uL3	b-HP-2	A
	G	ABE	eL33	G	A
	loop-2	A	uL6	G	ABE
uL14	G	ABE	eL24	G	A
	seg-N	A	uL3	loop-1	A
	loop-1	A	uL3	seg-N	A
uL15	G	ABE	eL18	G	A
	mix-N	ABE	eL32	loop-1	A
	mix-N	ABE	uL33	loop-1	A
uL16	G	ABE	eL20	G	A
uL18	mix-N	A	uL5	loop-2	A
	mix-N	A	eL21	seg-N	A
uL23	loop-1	A	eL39	mix-NC	A
uL24	mix-N	A	uL4	loop-2	A
uL33	loop-1	A	uL15	mix-N	ABE
	loop-1	A	eL15	loop-1	A

eL8	G	A	eL15	G	A
eL14	G	A	uL6	G	ABE
	G	A	eL20	G	A
eL15	G	A	eL8	G	A
	loop-1	A	uL2	mix-N	ABE
	loop-1	A	uL33	loop-1	A
eL18	G	A	uL4	G	ABE
	G	A	uL15	G	ABE
eL19	G	A	eL34	seg-N	A
	mix-C	A	uS17	G	ABE
eL20	G	A	uL16	G	ABE
	G	A	eL14	G	A
	mix-C	A	uL6	G	ABE
eL21	seg-N	A	uL18	mix-N	A
	seg-N	A	PTC		ABE
eL24	G	A	uL3	G	ABE
	G	A	uL14	G	ABE
	G	A	eS6	G	A
eL27	G	A	eL30	G	A
	G	A	eL34	G	A
	G	A	eL34	hel-C	A
eL30	G	A	uS15	G	ABE
	G	A	eL27	G	A
	G	A	eL34	hel-C	A
	G	A	eL43	G	A
eL32	loop-1	A	uL15	mix-N	ABE
	loop-1	A	eL33	loop-1	A
eL33	G	A	uL13	G	ABE
	loop-1	A	eL32	loop-1	A

eL34	G	A	eL27	G	A
	seg-N	A	eL39	mix-NC	A
	seg-N	A	eL19	G	A
	hel-C	A	eL27	G	A
	hel-C	A	eL30	G	A
eL37	mix-NC	A	tunnel		ABE
	mix-NC	A	uL4	loop-1	ABE
	mix-NC	A	eL39	mix-NC	A
eL39	mix-NC	A	tunnel		ABE
	mix-NC	A	uL23	loop-1	A
	mix-NC	A	eL37	mix-NC	A
	mix-NC	A	eL34	seg-N	A

eL40	G	A	uL6	G	ABE
eL43	G	A	uL2	G	ABE
	G	A	eL30	G	A
	[illegible]	[illegible]	[illegible]	[illegible]	[illegible]

Table 3 (continued) : interaction acquired in the eukaryotic network

Protein	part	status	Node	part	status
uS2	G	ABE	eS17	mix-C	E
uS2	G	ABE	eS21	loop-1	E
	G	ABE	eS21	mix-C	E
	seg-C	ABE	eS17	mix-C	E
uS3	G	ABE	eS10	G	E
	G	ABE	eS10	seg-C	E
	seg-C	ABE	RACK1	RACK1	E
uS4	G	ABE	eS30	mix-NC	E
	dom-1	A	eS4	mix-C	E
uS7	G	ABE	eS25	G	E
uS8	G	ABE	eS7	G	E
uS9	G	ABE	RACK1	RACK1	E
uS11	G	ABE	eS26	loop-1	E
uS11	loop-1	A	eS26	G	E
	seg-C	ABE	eS26	G	E
uS12	G	ABE	eS30	mix-NC	E
uS13	seg-N	ABE	eS25	G	E
uS14	mix-NC	ABE	eS10	G	E
uS15	seg-N	A	eS7	G	E
uS17	G	ABE	eL19	G	A
	seg-N	E	eS8	G	A
	seg-N	E	eS8	dom-1	E
	seg-N	E	eL19	hel-C	E
	mix-C	ABE	eL19	mix-C	A

eS1	G	A	eL8	mix-C	E
eS1	G	A	eS26	G	E
eS1	G	A	eS26	loop-1	E
eS1	loop-1	E	eS17	mix-C	E
eS4	G	A	eS6	mix-C	E
eS4	mix-C	E	uS4	dom-1	A
eS6	G	A	uL3	G	ABE
eS6	G	A	eL24	mix-C	E
eS6	G	A	eS24	mix-C	E
eS6	mix-C	E	eS4	G	A
eS7	G	E	uS8	G	ABE
eS7	G	E	uS15	seg-N	A
eS7	G	E	eS27	mix-N	A
eS7	G	E	eL19	hel-C	E
eS8	G	A	uS17	seg-N	E
eS8	dom-1	E	uS17	seg-N	E
eS10	G	E	uS3	G	ABE
eS10	G	E	uS14	mix-NC	ABE
eS10	G	E	eS12	G	E
eS10	G	E	eS12	loop-1	E
eS10	seg-C	E	uS3	G	ABE
eS12	G	E	eS31	G	A
eS12	G	E	eS10	G	E
eS12	loop-1	E	eS10	G	E
eS17	G	A	RACK1	RACK1	E
eS17	mix-C	E	uS2	G	ABE
eS17	mix-C	E	uS2	seg-C	ABE
eS17	mix-C	E	eS1	loop-1	E
eS21	loop-1	E	uS2	G	ABE
eS21	mix-C	E	uS2	G	ABE
eS21	mix-C	E	eS27	mix-N	A
eS24	mix-C	E	eS6	G	A
eS25	G	E	uS13	seg-N	ABE
eS25	G	E	uS7	G	ABE
eS25	G	E	uL1	G	ABE
eS25	G	E	tRNA-E	AC-stem	ABE
eS26	G	E	eS1	G	A
eS26	loop-1	E	eS28	seg-C	A
eS26	G	E	uS11	loop-1	A
eS26	G	E	uS11	seg-C	ABE
eS26	seg-N	E	eS26	seg-C	E
eS26	loop-1	E	uS11	G	ABE
eS26	loop-1	E	eS1	G	A
eS26	seg-C	E	eS26	seg-N	E
eS27	mix-N	A	eS21	mix-C	E
eS27	mix-N	A	eS7	G	E
eS28	seg-C	A	eS26	loop-1	E
eS30	mix-NC	E	tRNA-A	AC-stem	ABE
eS30	mix-NC	E	uS4	G	ABE
eS30	mix-NC	E	uS12	G	ABE
eS31	G	A	eS12	G	E
eS31	[illegible]	E	tRNA-A	AC-stem	ABE

Protein	part	status	Node	part	status
uL1	G	ABE	eS25	G	E
uL1	G	ABE	eL13	mix-C	E
uL2	loop-1	E	eL8	mix-N	E
uL3	G	ABE	eS6	G	A
	hel-C	E	eL24	mix-C	E
uL4	loop-1	ABE	eL15	mix-C	E
	loop-1	ABE	eL13	mix-N	E
	mix-C	E	uL30	mix-N	E
	mix-C	E	eL18	G	A
	mix-C	E	eL20	dom-1	E
	mix-C	E	eL21	mix-C	E
uL5	loop-1	ABE	uL33	seg-C	E
uL6	G	ABE	eL14	seg-N	E
uL13	G	ABE	eL20	seg-C	E
	hel-C	E	eL14	hel-C	E
uL15	G	ABE	eL13	mix-C	E
	G	ABE	eL36	mix-N	E
	mix-N	ABE	eL18	mix-C	E
	mix-N	ABE	eL13	mix-N	E
uL16	G	ABE	eL21	mix-C	E
	mix-C	E	uL18	mix-C	E
uL18	mix-C	E	uL16	mix-C	E
uL22	mix-C	E	eL33	loop-2	E
uL23	seg-N	E	uL29	mix-C	E
uL23	seg-N	E	eL8	mix-N	E
uL24	G	ABE	eL37	mix-C	E
	G	ABE	eL39	mix-NC	A
uL29	mix-C	E	uL23	seg-N	E
	mix-C	E	eL15	G	A
	mix-C	E	eL13	G	E
	mix-C	E	eL37	mix-C	E
uL30	G	ABE	eL18	seg-N	E
	G	ABE	eL20	dom-1	E
	mix-N	E	uL4	mix-C	E
	mix-N	E	eL20	dom-1	E
	mix-N	E	eL21	mix-C	E
uL33	seg-C	E	uL5	loop-1	ABE

eL6	G	E	eL14	hel-C	E
	G	E	eL33	seg-N	E
	G	E	eL33	seg-C	E
	seg-N	E	eL32	G	A
	seg-C	E	eL14	hel-C	E
	seg-C	E	eL33	seg-N	E
eL8	G	A	eL36	mix-N	E
	mix-N	E	uL2	loop-1	E
	mix-N	E	uL23	seg-N	E
	mix-N	E	eL27	loop-1	E
	mix-C	E	eS1	G	A
eL13	G	E	uL29	mix-C	E
	G	E	eL36	mix-N	E
	mix-N	E	uL4	loop-1	ABE
	mix-N	E	uL15	mix-N	ABE
	mix-N	E	eL15	mix-C	E
	mix-N	E	eL18	mix-C	E
	mix-C	E	uL1	G	ABE
	mix-C	E	uL15	G	ABE
	mix-C	E	eL36	mix-N	E
eL14	seg-N	E	uL6	G	ABE
	seg-N	E	eL20	seg-C	E
	hel-C	E	uL13	hel-C	E
	hel-C	E	eL6	G	E
	hel-C	E	eL6	seg-C	E
eL15	G	A	uL29	mix-C	E
	G	A	eL36	G	E
	mix-C	E	uL4	loop-1	ABE
	mix-C	E	eL13	mix-N	E
eL18	G	A	uL4	mix-C	E
	seg-N	E	uL30	G	ABE
	mix-C	E	uL15	mix-N	ABE
	mix-C	E	eL13	mix-N	E
eL19	mix-C	A	uS17	seg-N	E
	hel-C	E	uS17	G	ABE
	hel-C	E	uS17	mix-C	ABE
	hel-C	E	eS7	G	E

Coevolutions in Eukarya

Coevolution of extensions in ABE proteins

Protein(ABE)	Ext	E	Protein(ABE)	ext	E

ABE Target of new contact

Protein(ABE)	G/ext	ABE	Protein(ABE)	ext	E
Protein(ABE)	G/ext	ABE	Protein(E)	G/ext	E

ABE Donnor of new contact

Protein(ABE)	ext	E	Protein(ABE)	G/ext	AB[illegible]
Protein(E)	G/ext	E	Protein(ABE)	G/ext	AB[illegible]

Coevolutions of extensions in A proteins

Protein (A)	ext	E	Protein(A)	ext	E

A Target of new contact

Protein(A)	G/ext	ABE	Protein(A)	ext	E
Protein(A)	G/ext	ABE	Protein(E)	G/ext	E

A Donnor of new contact

Protein(A)	ext	E	Protein(A)	G/ext	AB[illegible]
Protein(E)	G/ext	E	Protein(A)	G/ext	AB[illegible]

Coevolutions of extensions in ABE and A proteins

Protein (A)	ext	E	Protein(ABE)	ext	E

ABE or A Target of new contact

Protein(ABE)	G/ext	ABE	Protein(A)	ext	E
Protein(A)	G/ext	ABE	Protein(ABE)	ext	E

ABE or A Donnor of new contact

Protein(A)	ext	E	Protein(ABE)	G/ext	AB[illegible]
Protein(ABE)	G/ext	E	Protein(A)	G/ext	AB[illegible]

eL20	dom-1	E	uL4	mix-C	E
	dom-1	E	uL30	G	ABE
	dom-1	E	uL30	mix-N	E
	loop-1	E	eL21	mix-C	E
	seg-C	E	uL13	G	ABE
	seg-C	E	eL14	seg-N	E
eL21	G	A	eL29	mix-NC	E
	mix-C	E	uL4	mix-C	E
	mix-C	E	uL16	G	ABE
	mix-C	E	uL30	mix-N	E
	mix-C	E	eL20	loop-1	E
eL24	mix-C	E	uL3	hel-C	E
	mix-C	E	eS6	G	A
eL27	loop-1	E	eL8	mix-N	E
eL29	mix-NC	E	eL21	G	A
	mix-NC	E	PTC		ABE
eL32	G	A	eL6	seg-N	E
eL33	seg-N	E	eL6	G	E
	seg-N	E	eL6	seg-C	E
	loop-2	E	uL22	mix-C	E
	seg-C	E	eL6	G	E
eL36	mix-N	E	eL8	G	A
	G	E	eL15	G	A
	mix-N	E	uL15	G	ABE
	mix-N	E	eL13	G	E
	mix-N	E	eL13	mix-C	E
eL37	mix-C	E	uL24	G	ABE
	mix-C	E	uL29	mix-C	E
eL39	mix-NC	A	uL24	G	ABE

Table 4

Bacteria

bL21	bL20	2
bL25	uL16	2
bL9	bL28	2
bS18	bS6	2
uL13	bL20	2
uL16	tRNA-A	2
uL16	tRNA-P	2
uL22	bL32	2
uL3	bL19	2
uS13	bL31	2
uS3	uS14	2
uS5	uS4	2
uS5	uS8	2
uS7	uS9	2
uL5	bL31	3
uS3	uS10	3

Archaea

eL34	eL27	2
eS21	uS8	2
eS28	uS11	2
eS4	uS17	2
mRNA	uS11	2
uL16	tRNA-A	2
uL16	tRNA-P	2
uL3	PTC	2
uL3	uL13	2
uL33	tRNA-E	2
uS11	eS1	2
uS15	eS27	2
uS3	uS14	2
uS5	uS4	2
uS5	uS8	2
uS7	uS9	2
uL2	eL43	3
uL3	uL14	3
uS3	uS10	3
eS21	uS5	4

Eukarya

eL14	eL20	2
eL14	eL6	2
eL18	uL15	2
eL34	eL27	2
eL36	eL13	2
eS10	uS3	2
eS12	eS10	2
eS21	uS8	2
eS26	eS1	2
eS28	uS11	2
eS4	uS17	2
eS4	uS4	2
eS6	eL24	2
mRNA	uS11	2
uL15	eL13	2
uL16	tRNA-A	2
uL16	tRNA-P	2
uL29	uL23	2
uL3	PTC	2
uL3	eL24	2
uL3	uL13	2
uL30	eL20	2
uL33	tRNA-E	2
uL4	eL18	2
uL6	eL14	2
uS11	eS1	2
uS15	eS27	2
uS3	uS14	2
uS5	uS4	2
uS5	uS8	2
uS7	uS9	2
eL6	eL33	3
eS17	uS2	3
eS26	uS11	3
uL2	eL43	3
uL3	uL14	3
uS17	eL19	3
uS17	eS8	3
uS2	eS21	3
uS3	uS10	3
eS21	uS5	4

Table5

Kingdom		ABE		B		A				E	
LSU	uL1	seg-N									
	uL2	mix-N	mix-C							loop-1	E
	uL3	loop-3				seg-N	loop-1	b-HP-2	seg-C	hel-C	A + E
	uL4	loop-1				loop-2				mix-C	A + E
	uL5	loop-1		mix-N		loop-2					B, A
	uL6										
	uL10										
	uL11										
	uL13	loop-1		seg-N		loop-2				hel-C	B, A + E
	uL14					seg-N	loop-1				A
	uL15	mix-N									
	uL16	seg-N	loop-1							mix-C	E
	uL18					mix-N				mix-C	A + E
	uL22	b-HP				loop-1				mix-C	A + E
	uL23			b-HP		loop-1				seg-N	B, A + E
	uL24	b-HP				mix-N					A
	uL29									mix-C	E
	uL30									mix-N	E
	uL33					loop-1				seg-C	A + E
SSU	uS2	seg-C		dom-1							B
	uS3	seg-C		mix-N		hel-N					B, A
	uS4			dom-1	dom-2	seg-N	dom-1	mix-C			B, A
	uS5	b-HP	mix-C			dom-1	loop-1				A
	uS7	mix-N	loop-1								
	uS8			loop-1							B
	uS9	seg-C									
	uS10	b-HP									
	uS11	seg-N	seg-C			loop-1					A
	uS12	mix-N	loop-1								
	uS13	seg-N	mix-C								
	uS14	mix-NC									
	uS15					seg-N	dom-1	mix-C			A
	uS17	b-HP	mix-C			loop-3				seg-N	A + E
	uS19	seg-N									

Kingdom		A		E			
LSU	eL8			mix-N	loop-1	mix-C	E
	eL14			seg-N	hel-C		E
	eL15	loop-1	seg-C	mix-C			A + E
	eL18			seg-N	mix-C		E
	eL19	mix-C		hel-C			A + E
	eL20	mix-C		dom-1	loop-1	seg-C	A + E
	eL21	seg-N		mix-C			A + E
	eL24			mix-C			E
	eL27			loop-1			E
	eL30						
	eL32	loop-1					
	eL33	loop-1		seg-N	loop-2	seg-C	A + E
	eL34	seg-N	hel-C				
	eL37	mix-NC		mix-C			A + E
	eL39	mix-NC					
	eL40	mix-N					
	eL43	mix-N	mix-C				
SSU	eS1	seg-N	seg-C	loop-1			A + E
	eS4	seg-N	loop-1	mix-C			A + E
	eS6			mix-C			E
	eS8	loop-1		dom-1			A + E
	eS17			mix-C			E
	eS19	b-HP					
	eS21	seg-N		loop	mix-C		A + E
	eS24			mix-C			E
	eS27	mix-N					
	eS28	seg-N	seg-C				
	eS31			seg-N			E

Kingdom		B		
LSU	bL9	Dom-1	Hel-1	Dom-2
	bL12			
	bL17	Seg-N	Loop-1	
	bL19	mix-N	mix-C	
	bL20	mix-N		
	bL21	β-HP		
	bL27	Seg-N		
	bL28	β-HP		
	bL31	Seg-N	Seg-C	
	bL32	mix-N		
	bL34	mix-N		
	bL35	Loop-1		
SSU	bS6	seg-C		
	bS16			
	bS18	seg-C		
	bS20			

Kingdom		E		
LSU	eL6	seg-N	seg-C	
	eL13	mix-N	mix-C	
	eL22			
	eL29	mix-NC		
	eL36	mix-N		
	eL38			
SSU	eS7	loop-1		
	eS10	seg-C		
	eS12	loop-1		
	eS25			
	eS26	seg-N	loop-1	seg-C
	eS30	mix-NC		

protein	extension	protein	extension	Conserved residues in extensions	rRNA ES	Other proteins
**uL2**	loop-1	**eL8**	mix-N	**uL2**: **phe 63**, **arg 64**, tyr 67, lys 68, tyr 69, arg 70, **glu 74**<br><br>**eL8:** lys 51, **tyr 49, arg 48, leu 46, asn 45, arg 44,** lys 43, **pro 42**, ile 36, **phe 34**, asn 33, lys 32, thr 30	U2558*, A2557*, A2561*, A2580*, U2550*, G2549*, G2525*, A2524 *	-
**uL3**	hel-C	**eL24**	mix-C	uL3: **phe 370**, **lys 376**, phe 379, **gly 381**, leu 383<br><br>eL24: **lys 35, pro 46 trp 51, thr 52**, val 53, phe 55, **arg 56**, lys 57, his 59, **lys 60, lys 61**	-	-
**uL4**	mix-C	**uL30**	mix-N	uL4: **lys 314**, lys 315, **asn 316**, **pro 317**, **leu 318, asn 320,** val 323, leu 324, arg 326, **leu 327**, **asn 328, pro 329,** tyr 330<br><br>**uL30:** arg 41, arg 47, **tyr 51, glu 54, tyr 55**	A607*, C596, A608, G597	-
**uL4**	mix-C	**eL20**<br><br><br><br><br><br><br>**uL30**<br><br><br>**eL21**	dom-1<br><br><br><br><br><br><br>mix-N<br><br><br>mix-C	uL4: (pro 351, phe 355, lys 360, his 361, asp 362) variability intra-kingdom<br><br>eL20: phe 25, arg 26, **met 27**, arg 28, ile 29, **phe 30**<br><br>**uL30:** ala 65, **arg 67**, **ala 69, gly 73,** tyr 75, **tyr 76**<br><br>eL21: tyr 156, **pro 155**, val 154, **pro 153,** leu 151, val 141, arg 136, **pro 135**	G518*, A519*, U520*	uL4, uL30 and eL20 form a crevice for binding eL21 (E)
**eL6**	seg-C	**eL14**	hel-C	eL6: **tyr 158, arg 51, phe 163, leu 165, pro 171, his 172, lys 175**<br><br>eL14, gln 119, phe 118, **arg 117**, glu 116, **phe 115**, **asp 114**, thr 113, leu 112	A3209*, A3210, C3211, C3212, A3213*,	-
**eL6**	seg-C	**eL33**	seg-N	eL6: **arg 31**, tyr 83, **leu 43**, arg 48, **phe 163**, **leu 165, pro 171, his 172, lys 175**<br><br>**eL33: arg6,** tyr 8, lys 10, glu 33, **phe 43, tyr 44, leu 45, lys 47,** arg 48, **trp 68**, **arg 99, leu 102, tyr 103, pro 104**	A3213*, G3176	-
**eL8**	mix-N	**uL23**	seg-N	eL8: gln 59, val 56, tyr 55, **pro 53, trp 52, val 50**<br><br>**uL23: leu 34, phe 32,** ser 29, **arg 27,** val 26, leu 24, lys 25	A1558*, G2585*	-
**eL8**	mix-N	**eL27**	loop-1	eL8: leu 26, **pro 25,** thr 27<br>eL27: **pro 50,** lys 52, **val 53,** lys 55, **his 57**, lys 60, lys 61, lys 64, **arg 65**	A2562, G2563, G2564	-
**uL13**	hel-C	**eL14**	hel-C	uL13: ala 181, leu 194, **gly [illegible]**	-	-

				eL14: arg 109, **leu 112, glu 116,** gln 119, val 120, leu 123, arg 128		
**eL13**	mix-N	**eL18**<br><br><br><br><br><br><br>**uL15**	mix-C<br><br><br><br><br><br><br>mix-N	eL13: **asn 6**, leu 7<br><br>eL18: **his 152, phe 153, pro 163, ile 165,** ser 167, **thr 168, lys 171, phe 172, glu 173, arg 176, arg 178, arg 179,** phe 184, lys 185<br><br>uL15: phe 61, **arg 59, met 58, val 56, lys 55, phe 53, tyr 52, pro 50, his 49, tyr 48, lys 47, asp 46, arg 42, his 39**	-	eL13 and eL18 coevolved to form a common interface with uL15 mix-N (ABE)
**eL13**	mix-N	**eL15**<br><br><br><br><br><br><br><br><br>**uL4**	mix-C<br><br><br><br><br><br><br><br><br>loop-1	eL13: **his 13, phe 14, lys 16, trp 18, gln 19, val 22, val 24, his 25, phe 26, gln 28,** lys 31, **lys 32**<br><br>eL15: **trp 191**, lys 192, arg 193, **asn 195**, leu 197, **leu 199**, **arg 201, arg 203**<br><br>uL4: **arg 47, gln 48, tyr 50, val 52, pro 102, lys 104, trp 106, arg 107,** lys 108, **trp 109, asn 110**	-	eL13 and eL15 coevolved to form a common interface with uL4 loop-1 (ABE)
**eL13**	mix-C	**eL36**	mix-N	eL13: val 159, gln 160, phe 167, **leu 170, arg 171, arg 174**<br><br>**eL36: thr 18,** lys 15, **lys 13**, leu 11, **gly 10,** ile 9, ile 7<br><br>uL15: **pro 71**, trp 69, arg 128, phe 129, lys 132		eL13 and eL36 coevolved to bind the globular domain of uL15 (ABE)
**eL14**	seg-N	**eL20**	seg-C	eL14: asn 11, arg 13, leu 14, **glu 16, val 15**<br><br>**eL20: leu 152, pro 151, phe 150,** lys 149, leu 148	A3186*, A3187, G3205, C3206	
**uL16**	mix-C	**uL18**	mix-C	uL16: **gly 204**, asn 208, ile 210<br>uL18: arg 282, arg 285, val 286, **lys 289**	-	
**uL22**	mix-C	**eL33**	loop-2	-	-	-
**uL23**	seg-N	**uL29**	mix-C	uL23: val 51, **ser 48, tyr 46, lys 45, pro 44, arg 42**<br>uL29: **arg 81, leu 80, asp 79, pro 77,** tyr 75, **lys 74,** lys 73, **tyr 70**	-	-
**uL29**	mix-C	**eL37**	mix-C	uL29: **lys 83, thr 85, ala 87**, leu 88, **arg 86, arg 89**<br><br>**eL37:** tyr 66, **leu 67,** val 70, arg 73, **phe 74, phe 78**	-	-
**eS8**	dom-1	**uS17**	seg-N	eS8: **glu 185, glu 188, tyr 192, phe 191,** arg 194, arg 195<br>uS17: **phe 20, lys 15, gln 14,** phe 13, arg 11, **glu 10**	-	-

Table 6

protein	Ne	Np	Nc	Bet	Eig	size	cons	N_gly	R_gly	N_pro	R_pro	N_arm	R_arm	trp	his	tyr	phe	N_acid	R_acid	asp	glu	N_basi	R_basi	lys	arg	N_pol	R_pol	asn	gln	ser	thr	N_HP	R_HP	leu	val	ile	met	ala	N_cys	R_cys	function
**bS16**	0	0	0	0,00	0,00	88,00	46,59	4	4,55	2	2,27	4	4,55	1	0	1	0	5	5,68	2	1	8	9,09	1	4	1	1,14	0	0	0	0	17	19,32	1	2	2	0	1	0	0	
**bS20**	0	0	0	0,00	0,00	106,00	32,08	0	0	0	0	1	0,94	0	1	0	0	1	0,94	1	0	8	7,55	2	2	6	5,66	3	0	2	1	18	16,98	1	0	0	0	3	0	0	
**uL30**	0	0	0	0,00	0,00	0,00	0	0	0	0	0	0	0	0	0	0	0	0	0	0	0	0	0	0	0	0	0	0	0	0	0	0	0	0	0	0	0	0	0	0	
**bL12**	0	1	1	0,00	0,00	125,00	61,6	5	4	1	0,8	2	1,6	0	0	0	2	12	9,6	0	8	9	7,2	8	1	4	3,2	0	0	0	1	44	35,2	5	6	1	0	8	0	0	
**uL1**	1	1	1	0,00	0,04	0,00	0	0	0	0	0	0	0	0	0	0	0	0	0	0	0	0	0	0	0	0	0	0	0	0	0	0	0	0	0	0	0	0	0	0	
**uL10**	0	1	1	0,00	0,00	173,00	31,21	2	1,16	1	0,58	1	0,58	0	0	0	1	1	0,58	0	0	7	4,05	2	1	5	2,89	1	0	0	0	37	21,39	2	3	0	0	3	0	0	
**uL18**	0	1	1	0,00	0,25	112,00	52,68	5	4,46	0	0	7	6,25	0	1	2	2	4	3,57	1	1	12	10,71	0	8	4	3,57	0	1	1	0	27	24,11	4	3	1	0	8	0	0	double_R1-P1
**uL24**	1	1	1	0,00	0,02	110,00	44,55	7	6,36	0	0	1	0,91	0	1	0	0	3	2,73	2	1	12	10,91	3	2	3	2,73	2	0	1	0	23	20,91	0	5	1	0	0	0	0	tunnel
**uL29**	0	1	1	0,00	0,01	72,00	33,33	0	0	0	0	1	1,39	0	0	0	1	1	1,39	0	0	5	6,94	0	2	4	5,56	0	2	0	1	13	18,06	3	0	1	0	1	0	0	
**uL6**	0	1	1	0,00	0,24	0,00	0	0	0	0	0	0	0	0	0	0	0	0	0	0	0	0	0	0	0	0	0	0	0	0	0	0	0	0	0	0	0	0	0	0	
**bL25**	1	1	2	0,00	0,25	226,00	11,5	3	1,33	1	0,44	2	0,88	0	1	1	0	1	0,44	1	0	3	1,33	0	2	1	0,44	0	1	0	0	15	6,64	0	0	0	0	0	0	0	double_R1-P1
**bL34**	1	2	2	0,00	0,03	49,00	55,1	3	6,12	1	2,04	3	6,12	0	1	0	1	0	0	0	0	11	22,45	2	8	4	8,16	0	1	0	2	5	10,2	1	0	0	1	0	0	0	tunnel
**bL9**	1	1	2	0,00	0,01	148,00	33,78	7	4,73	0	0	5	3,38	0	0	1	1	1	0,68	0	0	3	2,03	0	0	3	2,03	1	0	1	0	31	20,95	3	2	1	1	3	0	0	
**uL11**	0	2	2	0,00	0,00	147,00	63,27	8	5,44	8	5,44	5	3,4	0	0	1	4	4	2,72	2	1	12	8,16	9	1	16	10,88	2	3	3	3	39	26,53	3	2	3	2	10	1	0,68	
**uL33**	0	2	2	0,00	0,06	54,00	40,74	0	0	1	1,85	4	7,41	0	0	1	0	1	1,85	0	1	8	14,81	3	1	4	7,41	1	0	0	1	4	7,41	1	0	0	0	0	0	0	tRNA-E
**uS15**	0	2	2	0,04	0,06	89,00	53,93	3	3,37	0	0	5	5,62	0	1	2	0	4	4,49	2	1	8	8,99	1	6	6	6,74	0	1	2	2	22	24,72	8	2	2	0	1	0	0	
**uS2**	2	2	2	0,01	0,10	256,00	49,61	9	3,52	5	1,95	13	5,08	2	2	0	3	12	4,69	5	3	14	5,47	5	3	14	5,47	5	1	0	6	60	23,44	10	3	6	3	9	0	0	
**uS4**	2	1	2	0,00	0,05	209,00	45,93	8	3,83	3	1,44	11	5,26	0	2	4	1	10	4,78	1	4	20	9,57	2	7	13	6,22	1	5	3	1	31	14,83	5	5	1	1	2	0	0	
**bL17**	2	3	3	0,04	0,28	118,00	44,92	3	2,54	0	0	3	2,54	0	0	1	1	3	2,54	1	2	13	11,02	3	8	4	3,39	0	0	0	3	27	22,88	4	1	3	0	3	0	0	double_T1-P2
**bL19**	2	2	3	0,00	0,24	146,00	45,89	8	5,48	2	1,37	7	4,79	0	0	2	3	5	3,42	1	4	13	8,9	3	7	5	3,42	0	1	2	2	27	18,49	2	6	3	0	2	0	0	single_P2
**bL28**	1	2	3	0,12	0,04	98,00	22,45	3	3,06	0	0	2	2,04	0	1	0	0	0	0	0	0	5	5,1	0	1	4	4,08	1	0	1	1	7	7,14	1	0	0	1	0	1	1,02	tRNA-E
**bL35**	1	3	3	0,41	0,07	65,00	38,46	1	1,54	1	1,54	2	3,08	0	1	0	1	1	1,54	0	0	8	12,31	5	2	4	6,15	0	0	0	2	8	12,31	1	0	0	1	0	0	0	tRNA-E
**bS18**	1	2	3	0,11	0,07	88,00	44,32	2	2,27	2	2,27	3	3,41	0	0	1	0	3	3,41	2	1	11	12,5	4	4	3	3,41	0	1	0	1	14	15,91	3	0	2	0	2	1	1,14	
**uL14**	0	3	3	0,04	0,43	122,00	72,13	8	6,56	4	3,28	4	3,28	0	0	0	2	7	5,74	4	2	15	12,3	4	7	11	9,02	2	1	3	2	39	31,97	5	9	6	2	7	0	0	PTC
**uL15**	1	3	3	0,40	0,07	150,00	38	16	10,67	2	1,33	2	1,33	0	0	0	2	1	0,67	0	1	9	6	4	5	6	4	0	2	1	1	21	14	3	0	1	0	1	0	0	double_R1-T1
**uL23**	1	3	3	0,12	0,03	96,00	37,5	2	2,08	1	1,04	2	2,08	0	0	0	2	2	2,08	0	1	8	8,33	7	1	3	3,12	0	0	0	0	18	18,75	1	5	0	0	3	0	0	tunnel
**uL4**	1	3	3	0,27	0,09	210,00	37,14	7	3,33	3	1,43	6	2,86	0	0	0	1	1	0,48	0	0	14	6,67	6	4	8	3,81	1	1	0	3	39	18,57	2	1	0	0	4	0	0	tunnel
**uS14**	1	2	3	0,00	0,09	61,00	52,46	3	4,92	2	3,28	3	4,92	1	0	0	1	0	0	0	0	12	19,67	3	6	3	4,92	0	0	2	0	8	13,11	0	0	0	1	2	1	1,64	
**uS17**	2	3	3	0,06	0,16	105,00	39,05	2	1,9	1	0,95	4	3,81	0	2	1	0	6	5,71	2	3	7	6,67	5	1	5	4,76	1	0	2	0	16	15,24	0	4	1	1	0	0	0	
**uS19**	2	3	3	0,00	0,55	93,00	65,59	6	6,45	3	3,23	9	9,68	1	2	0	2	4	4,3	1	1	15	16,13	6	4	8	8,6	0	0	3	4	16	17,2	2	3	1	2	0	0	0	SB
**bL21**	1	3	4	0,11	0,12	101,00	45,54	6	5,94	1	0,99	3	2,97	0	1	0	0	1	0,99	0	1	10	9,9	3	1	4	3,96	0	2	0	1	21	20,79	1	3	1	0	1	0	0	
**bS6**	1	3	4	0,19	0,10	101,00	31,68	1	0,99	0	0	4	3,96	1	0	3	0	4	3,96	0	2	4	3,96	0	2	1	0,99	0	0	0	0	18	17,82	1	0	1	0	1	0	0	SB
**uL13**	2	3	4	0,10	0,21	140,00	55	9	6,43	4	2,86	12	8,57	1	4	2	0	3	2,14	3	0	12	8,57	6	2	7	5	1	1	0	3	30	21,43	6	1	0	1	5	0	0	
**uL2**	2	3	4	0,19	0,26	276,00	59,06	26	9,42	12	4,35	13	4,71	2	5	5	1	12	4,35	4	5	27	9,78	4	14	18	6,52	5	1	2	3	54	19,57	3	3	6	2	7	1	0,36	PTC
**uL22**	1	3	4	0,31	0,20	113,00	46,9	3	2,65	0	0	2	1,77	0	1	0	0	1	0,88	0	0	11	9,73	4	6	6	5,31	2	0	2	0	30	26,55	2	2	1	0	6	0	0	tunnel
**uS11**	2	4	4	0,28	0,20	129,00	72,87	11	8,53	6	4,65	6	4,65	1	2	0	2	6	4,65	2	2	18	13,95	3	6	15	11,63	4	1	2	4	31	24,03	1	4	3	0	6	1	0,78	tRNA-E
**uS12**	2	4	4	0,41	0,37	135,00	68,15	9	6,67	5	3,7	6	4,44	0	3	3	0	4	2,96	2	2	23	17,04	8	12	17	12,59	2	4	3	4	27	20	8	8	2	1	3	1	0,74	mRNA
**uS9**	1	3	4	0,41	0,35	128,00	51,56	11	8,59	1	0,78	3	2,34	0	0	1	1	3	2,34	2	1	19	14,84	5	10	8	6,25	1	2	1	2	21	16,41	2	2	1	0	5	0	0	tRNA-P
**bL27**	1	5	5	0,43	1,00	86,00	55,81	10	11,63	0	0	3	3,49	0	0	0	2	2	2,33	2	0	10	11,63	3	4	10	11,63	2	1	2	2	13	15,12	2	3	1	0	2	0	0	double_R1-P1
**bL32**	1	4	5	0,51	0,41	60,00	31,67	3	5	0	0	2	3,33	0	1	1	0	0	0	0	0	5	8,33	0	2	1	1,67	0	0	1	0	8	13,33	0	1	0	1	1	0	0	PTC
**uS10**	1	3	5	0,10	0,16	105,00	68,57	3	2,86	5	4,76	6	5,71	0	2	0	0	9	8,57	4	1	11	10,48	2	6	9	8,57	0	1	1	6	29	27,62	6	4	5	1	1	0	0	single_R2
**uS13**	2	4	5	0,17	0,91	126,00	61,9	8	6,35	2	1,59	3	2,38	0	1	1	0	6	4,76	1	2	21	16,67	5	11	7	5,56	1	1	0	3	31	24,6	5	2	5	0	3	0	0	tRNA-A
**uS7**	2	4	5	0,40	0,25	156,00	61,54	4	2,56	3	1,92	10	6,41	1	2	2	1	10	6,41	3	5	22	14,1	4	11	7	4,49	3	1	0	0	40	25,64	2	4	1	4	8	0	0	single_R2
**uS8**	1	4	5	0,13	0,20	138,00	51,45	7	5,07	4	2,9	3	2,17	0	0	2	0	5	3,62	3	2	8	5,8	1	5	9	6,52	1	0	3	2	35	25,36	4	2	4	0	1	0	0	single_R2
**bL20**	1	4	6	0,14	0,21	118,00	57,63	4	3,39	0	0	8	6,78	1	0	3	2	3	2,54	1	0	21	17,8	6	8	3	2,54	1	0	1	0	29	24,58	4	2	2	1	8	0	0	double_T1-P1
**bL31**	2	3	6	0,00	0,55	71,00	42,25	3	4,23	2	2,82	7	9,86	0	2	1	2	1	1,41	0	0	5	7,04	1	1	4	5,63	0	0	1	2	8	11,27	0	1	1	1	0	0	0	SB
**uL3**	1	5	6	0,24	0,53	206,00	47,09	18	8,74	3	1,46	9	4,37	0	3	1	4	6	2,91	3	1	14	6,8	7	2	11	5,34	0	1	1	3	36	17,48	1	6	0	2	2	0	0	PTC
**uL5**	2	4	6	0,07	0,74	182,00	64,84	10	5,49	5	2,75	12	6,59	0	0	3	7	12	6,59	4	3	19	10,44	5	6	14	7,69	2	2	1	3	46	25,27	6	1	4	2	5	0	0	tRNA-P
**uS5**	2	4	6	0,14	0,19	162,00	54,32	15	9,26	2	1,23	3	1,85	0	1	0	1	5	3,09	1	3	12	7,41	5	4	8	4,94	1	0	1	3	43	26,54	2	9	2	0	9	0	0	mRNA
**uL16**	2	5	8	0,43	1,00	141,00	63,83	11	7,8	6	4,26	7	4,96	2	0	0	2	6	4,26	0	5	22	15,6	7	7	4	2,84	0	1	0	2	34	24,11	3	2	2	2	9	0	0	double_R1-P1
**uS3**	2	5	8	0,12	0,21	239,00	56,07	14	5,86	4	1,67	12	5,02	3	1	3	1	13	5,44	3	6	20	8,37	3	9	9	3,77	1	2	1	2	62	25,94	7	2	6	0	12	0	0	mRNA
**Av**	1,10	2,59	3,27	0,13	0,22	122,14	45,91	6,10	4,45	2,14	1,55	4,82	3,79	0,33	0,88	0,96	1,12	4,02	2,91	1,29	1,51	11,45	9,47	3,33	4,45	6,35	4,98	0,94	0,82	0,98	1,61	24,94	18,62	2,69	2,43	1,65	0,67	3,29	0,14	0,12	
**Sd**	0,76	1,36	1,93	0,15	0,25	60,13	17,35	5,25	2,95	2,44	1,52	3,61	2,37	0,68	1,14	1,23	1,37	3,74	2,30	1,40	1,84	6,38	4,97	2,49	3,52	4,56	3,00	1,27	1,07	1,05	1,56	14,97	7,66	2,41	2,39	1,83	0,93	3,31	0,35	0,35	

Table 7: Bacterial network

protein	Ne	Np	Nc	Bet	Eig	size	cons	N_gly	R_gly	N_pro	R_pro	N_arm	R_arm	trp	his	tyr	phe	N_acid	R_acid	asp	glu	N_basi	R_basi	lys	arg	N_pol	R_pol	asn	gln	ser	thr	N_HP	R_HP	leu	val	ile	met	ala	N_cys	R_cys	function
eS31	0	0	0	0,00	0,00	52	40,38	3	5,77	1	1,92	4	7,69	0	1	2	0	1	1,92	0	1	3	5,77	0	1	1	1,92	0	0	0	1	4	7,69	0	0	0	0	1	4	7,69	
uL30	0	0	0	0,00	0,00	155	43,87	7	4,52	3	1,94	4	2,58	0	1	0	0	5	3,23	0	2	10	6,45	3	4	4	2,58	0	0	0	1	35	22,58	7	0	1	2	2	0	0	
eL40	0	1	1	0,00	0,01	51	33,33	0	0	0	0	0	0	0	0	0	0	1	1,96	0	0	6	11,76	1	3	1	1,96	1	0	0	0	5	9,8	1	0	0	0	2	4	7,84	
eL8	0	1	1	0,00	0,00	124	64,52	6	4,84	4	3,23	5	4,03	0	1	2	1	10	8,06	1	4	9	7,26	3	1	5	4,03	1	0	0	1	41	33,06	3	5	3	0	7	0	0	
eS6	0	1	1	0,00	0,00	125	44,8	10	8	3	2,4	2	1,6	0	0	0	1	5	4	2	0	8	6,4	1	3	5	4	1	2	0	1	23	18,4	2	1	3	1	0	0	0	SB
uL1	1	1	1	0,00	0,04	216	53,24	9	4,17	8	3,7	4	1,85	0	0	1	3	9	4,17	2	2	20	9,26	4	3	8	3,7	2	0	1	1	57	26,39	4	4	1	4	10	0	0	tRNA-E
uL10	0	1	1	0,00	0,00	339	33,04	6	1,77	6	1,77	3	0,88	1	0	0	2	6	1,77	1	0	8	2,36	2	1	5	1,47	1	0	0	2	78	23,01	7	2	1	0	8	0	0	
uL11	0	1	1	0,00	0,00	166	45,18	11	6,63	5	3,01	3	1,81	0	0	0	0	8	4,82	1	1	4	2,41	3	0	5	3,01	1	0	1	2	39	23,49	2	6	1	0	2	0	0	
uL22	2	1	1	0,00	0,00	157	38,22	5	3,18	3	1,91	7	4,46	0	2	1	0	3	1,91	0	2	11	7,01	2	3	4	2,55	2	0	1	0	27	17,2	3	1	2	0	6	0	0	tunnel
uL29	0	1	1	0,00	0,00	71	33,8	1	1,41	0	0	0	0	0	0	0	0	4	5,63	0	4	4	5,63	0	2	2	2,82	1	0	0	1	13	18,31	2	0	1	0	1	0	0	
eL14	0	2	2	0,00	0,01	83	65,06	6	7,23	1	1,2	2	2,41	0	1	0	1	8	9,64	2	4	9	10,84	2	5	3	3,61	2	0	0	0	25	30,12	0	5	1	1	3	0	0	
eL18	0	2	2	0,31	0,01	148	35,81	5	3,38	3	2,03	3	2,03	1	0	0	1	1	0,68	0	0	6	4,05	2	1	5	3,38	1	0	1	1	30	20,27	4	5	3	0	3	0	0	
eL19	1	2	2	0,37	0,20	150	43,33	9	6	1	0,67	5	3,33	1	0	2	1	4	2,67	0	0	19	12,67	1	9	3	2	0	1	1	1	24	16	5	0	3	0	5	0	0	SB
eL21	1	2	2	0,09	0,01	96	47,92	9	9,38	3	3,12	5	5,21	0	1	0	0	0	0	0	0	9	9,38	2	4	3	3,12	0	0	1	0	17	17,71	2	1	0	0	1	0	0	PTC
eL32	1	2	2	0,17	0,00	126	42,06	6	4,76	4	3,17	4	3,17	1	1	0	1	2	1,59	0	0	16	12,7	2	4	4	3,17	1	0	1	0	17	13,49	1	3	2	0	1	0	0	
eL33	1	2	2	0,15	0,00	87	44,83	7	8,05	1	1,15	4	4,6	0	1	1	1	0	0	0	0	4	4,6	0	0	2	2,3	0	2	0	0	21	24,14	2	3	1	0	3	0	0	
eS1	2	1	2	0,00	0,08	198	41,41	3	1,52	2	1,01	8	4,04	2	0	2	2	8	4,04	2	2	15	7,58	3	4	10	5,05	0	1	2	5	36	18,18	1	0	2	0	2	0	0	
eS17	0	2	2	0,01	0,20	67	43,28	2	2,99	0	0	4	5,97	0	0	1	2	2	2,99	1	0	6	8,96	3	1	5	7,46	2	0	0	1	10	14,93	1	1	0	0	1	0	0	
eS19	1	2	2	0,04	0,05	150	50,67	10	6,67	5	3,33	8	5,33	3	0	4	0	6	4	2	2	10	6,67	2	5	7	4,67	0	2	1	2	30	20	5	1	1	1	3	0	0	single_R2
eS24	0	2	2	0,00	0,25	99	34,34	1	1,01	1	1,01	4	4,04	0	0	1	0	3	3,03	0	2	3	3,03	0	1	4	4,04	1	0	0	1	18	18,18	0	0	0	0	0	0	0	
eS8	1	2	2	0,00	0,28	127	48,82	9	7,09	1	0,79	2	1,57	0	0	0	0	3	2,36	1	1	11	8,66	5	3	12	9,45	3	2	1	3	24	18,9	1	3	1	0	4	0	0	
uL18	1	2	2	0,04	0,01	188	46,28	10	5,32	2	1,06	10	5,32	0	1	4	1	6	3,19	3	1	12	6,38	1	8	6	3,19	1	1	2	1	41	21,81	5	2	2	0	8	0	0	double_R1-P1
uL23	1	2	2	0,13	0,01	94	41,49	2	2,13	0	0	1	1,06	0	0	0	0	3	3,19	0	1	5	5,32	4	0	5	5,32	1	0	0	3	23	24,47	2	3	2	0	3	0	0	
uL24	2	2	2	0,00	0,01	124	40,32	6	4,84	2	1,61	2	1,61	0	1	0	0	6	4,84	3	0	7	5,65	1	4	5	4,03	0	1	3	0	22	17,74	4	4	1	0	1	0	0	tunnel
uS19	2	2	2	0,00	0,03	132	59,09	8	6,06	3	2,27	10	7,58	0	3	1	4	7	5,3	0	4	12	9,09	2	7	9	6,82	1	0	2	3	29	21,97	4	1	1	1	1	0	0	SB
eL15	2	3	3	0,32	0,02	198	53,54	10	5,05	3	1,52	12	6,06	3	1	3	0	10	5,05	3	4	31	15,66	4	14	9	4,55	2	1	2	2	31	15,66	3	5	3	0	5	0	0	double_R1-P1
eL20	1	3	3	0,16	0,02	77	24,68	2	2,6	0	0	4	5,19	0	0	1	1	1	1,3	0	1	2	2,6	0	1	2	2,6	0	0	2	0	8	10,39	0	0	2	0	0	0	0	double_R1-P2
eL24	0	3	3	0,13	0,01	69	42,03	4	5,8	2	2,9	5	7,25	1	0	0	1	1	1,45	1	0	5	7,25	1	2	3	4,35	0	0	1	0	5	7,25	0	1	0	0	0	4	5,8	SB
eL27	0	2	3	0,00	0,07	83	65,06	6	7,23	1	1,2	2	2,41	0	1	0	1	8	9,64	2	4	9	10,84	2	5	3	3,61	2	0	0	0	25	30,12	0	5	1	1	3	0	0	
eL37	1	3	3	0,07	0,02	57	50,88	5	8,77	0	0	5	8,77	1	1	1	1	0	0	0	0	9	15,79	3	3	5	8,77	0	0	1	1	1	1,75	0	0	0	0	0	4	7,02	single_T2
eS27	1	2	3	0,00	0,37	63	39,68	3	4,76	1	1,59	2	3,17	0	0	0	2	1	1,59	0	1	2	3,17	1	0	2	3,17	1	1	0	0	10	15,87	1	2	0	0	0	4	6,35	
eS28	2	2	3	0,00	0,16	71	67,61	7	9,86	1	1,41	0	0	0	0	0	0	7	9,86	1	4	8	11,27	0	4	5	7,04	1	1	0	3	20	28,17	2	3	1	0	2	0	0	single_R2
uL15	1	3	3	0,50	0,01	146	35,62	14	9,59	0	0	2	1,37	0	0	0	1	1	0,68	0	0	11	7,53	5	3	4	2,74	0	0	0	1	20	13,7	2	0	0	0	1	0	0	
uS14	1	2	3	0,00	0,16	56	48,21	3	5,36	0	0	5	8,93	0	0	2	2	1	1,79	0	1	7	12,5	1	3	0	0	0	0	0	0	7	12,5	1	0	0	0	1	4	7,14	
uS2	1	3	3	0,03	0,38	202	55,45	8	3,96	9	4,46	14	6,93	1	1	4	5	14	6,93	7	3	12	5,94	2	7	10	4,95	3	1	0	4	45	22,28	8	2	4	1	3	0	0	
eL30	0	4	4	0,58	0,18	99	46,46	6	6,06	2	2,02	2	2,02	0	0	0	0	2	2,02	0	0	3	3,03	1	0	4	4,04	1	0	1	1	27	27,27	1	1	1	0	1	0	0	SB
eL39	1	4	4	0,63	0,04	51	50,98	0	0	2	3,92	2	3,92	2	0	0	0	0	0	0	0	9	17,65	4	4	3	5,88	1	0	0	1	10	19,61	2	1	0	0	1	0	0	tunnel
eL43	2	2	4	0,42	0,05	116	27,59	8	6,9	2	1,72	6	5,17	1	0	2	0	0	0	0	0	7	6,03	1	4	1	0,86	0	0	0	0	4	3,45	0	0	1	0	0	4	3,45	
uL13	2	3	4	0,20	0,01	145	45,52	8	5,52	5	3,45	3	2,07	0	0	1	0	2	1,38	0	1	12	8,28	2	3	4	2,76	1	0	1	0	32	22,07	3	0	1	1	5	0	0	single_P2
uL33	1	3	4	0,59	0,05	94	32,98	2	2,13	2	2,13	3	3,19	0	2	1	0	2	2,13	0	1	12	12,77	2	2	1	1,06	0	0	0	0	5	5,32	1	1	0	1	0	4	4,26	tRNA-E
uL4	2	4	4	0,27	0,01	246	46,75	16	6,5	7	2,85	5	2,03	0	1	1	1	9	3,66	3	1	20	8,13	3	9	8	3,25	2	1	1	3	50	20,33	4	5	1	0	10	0	0	single_T2
uL5	2	4	4	0,12	0,04	177	46,89	10	5,65	4	2,26	11	6,21	0	2	1	4	6	3,39	3	1	11	6,21	2	4	6	3,39	1	0	0	3	35	19,77	3	4	3	2	2	0	0	tRNA-P
uS12	2	4	4	0,90	0,60	147	65,31	14	9,52	5	3,4	7	4,76	1	1	1	1	9	6,12	5	4	19	12,93	7	7	9	6,12	3	2	2	1	32	21,77	6	9	4	0	5	1	0,68	mRNA
uS13	2	4	4	0,23	0,07	148	50,68	10	6,76	1	0,68	5	3,38	1	2	0	0	8	5,41	4	0	15	10,14	0	6	7	4,73	1	1	0	1	29	19,59	3	3	0	0	1	0	0	tRNA-A
eL34	2	4	5	0,69	0,12	89	44,94	3	3,37	3	3,37	3	3,37	0	1	0	0	0	0	0	0	13	14,61	1	4	3	3,37	0	0	2	0	11	12,36	1	0	0	0	0	4	4,49	
eS4	2	4	5	0,07	0,44	243	39,09	11	4,53	4	1,65	8	3,29	0	2	2	0	6	2,47	4	1	14	5,76	3	3	7	2,88	1	1	0	1	45	18,52	3	2	2	0	1	0	0	
uL14	2	3	5	0,11	0,02	133	62,41	8	6,02	4	3,01	3	2,26	0	0	1	1	9	6,77	4	4	15	11,28	1	6	8	6,02	2	1	0	0	36	27,07	1	4	3	0	8	0	0	PTC
uL6	0	5	5	0,33	0,02	181	46,41	9	4,97	2	1,1	10	5,52	0	2	3	3	6	3,31	2	3	7	3,87	0	2	9	4,97	2	1	0	3	41	22,65	0	7	5	1	3	0	0	PTC
uS10	1	3	5	0,13	0,19	102	62,75	4	3,92	5	4,9	3	2,94	1	1	0	0	7	6,86	2	4	12	11,76	1	5	3	2,94	0	0	0	3	30	29,41	6	2	6	2	3	0	0	single_R2
uS15	3	4	5	0,29	0,54	158	47,47	5	3,16	6	3,8	7	4,43	1	1	3	0	9	5,7	3	3	14	8,86	3	3	4	2,53	0	0	3	0	30	18,99	8	1	3	0	2	0	0	SB
uS4	3	4	5	0,11	0,60	180	45,56	5	2,78	4	2,22	11	6,11	2	3	1	0	6	3,33	1	4	15	8,33	2	6	8	4,44	1	3	1	2	33	18,33	10	2	3	1	3	0	0	
uS9	1	4	5	0,44	0,15	135	58,52	7	5,19	2	1,48	4	2,96	0	0	2	0	8	5,93	3	4	17	12,59	5	8	7	5,19	1	2	1	1	34	25,19	2	2	0	0	8	0	0	tRNA-P
uL16	2	4	6	0,78	0,07	216	38,89	7	3,24	4	1,85	9	4,17	0	1	3	1	5	2,31	1	3	18	8,33	2	7	6	2,78	1	1	0	1	35	16,2	2	0	1	3	8	0	0	tRNA-A
uS7	2	5	6	0,47	0,34	215	47,44	5	2,33	6	2,79	10	4,65	1	1	2	3	12	5,58	4	7	16	7,44	4	8	9	4,19	4	1	0	2	44	20,47	4	5	4	1	7	0	0	single_R2
uL2	2	4	7	0,63	0,03	240	54,58	28	11,67	11	4,58	9	3,75	0	2	0	0	8	3,33	1	4	20	8,33	3	9	11	4,58	3	2	2	2	44	18,33	1	5	1	2	6	0	0	PTC
uS17	3	6	7	0,67	0,72	113	47,79	5	4,42	4	3,54	7	6,19	0	2	2	0	3	2,65	2	1	9	7,96	2	5	3	2,65	0	0	1	1	20	17,7	1	5	1	0	1	3	2,65	SB
uL3	5	4	8	0,22	0,02	338	50	23	6,8	12	3,55	17	5,03	2	3	4	3	11	3,25	1	5	26	7,69	9	13	17	5,03	1	3	4	4	63	18,64	0	1	0	1	2	0	0	PTC
uS11	3	5	8	0,31	0,33	137	67,15	12	8,76	6	4,38	6	4,38	1	3	1	0	7	5,11	3	2	14	10,22	0	5	12	8,76	2	1	3	5	35	25,55	1	4	6	1	12	0	0	tRNA-E
uS3	2	5	8	0,25	0,48	210	43,81	12	5,71	5	2,38	6	2,86	0	0	1	2	6	2,86	1	2	14	6,67	3	2	5	2,38	0	1	1	2	44	20,95	2	4	3	1	6	0	0	mRNA
uS8	0	7	9	0,52	1,00	130	54,62	9	6,92	3	2,31	9	6,92	0	1	3	1	6	4,62	2	3	5	3,85	1	1	7	5,38	2	0	1	1	32	24,62	7	4	2	2	3	0	0	single_R2
uS5	4	7	13	0,38	0,86	236	49,58	17	7,2	6	2,54	8	3,39	3	1	1	1	6	2,54	3	3	12	5,08	3	2	15	6,36	3	1	2	5	50	21,19	7	8	3	0	8	3	1,27	single_R2
Av	1,28	2,84	3,54	0,21	0,15	141,41	47,08	7,49	5,24	3,21	2,09	5,39	3,89	0,51	0,82	1,13	0,92	5,05	3,51	1,43	1,84	11,02	8,20	2,18	4,05	5,62	4,00	1,07	0,62	0,82	1,39	28,07	19,19	2,69	2,44	1,61	0,51	3,26	0,70	0,96	
Sd	2,83	4,95	9,19	0,27	0,61	130,11	6,51	9,90	1,01	3,54	0,44	2,83	3,04	2,12	0,00	0,71	0,71	3,54	0,44	2,12	1,41	6,36	0,49	2,12	0,71	9,90	3,14	2,12	0,71	1,41	2,83	32,53	9,55	4,95	5,66	2,12	0,00	4,95	0,71	4,54	

Table 7 (continued) : Archaeal network

protein	Ne	Np	Nc	Bet	Eig	size	cons	N_gly	R_gly	N_pro	R_pro	N_arm	R_arm	trp	his	tyr	phe	N_acid	R_acid	asp	glu	N_basi	R_basi	lys	arg	N_pol	R_pol	asn	gln	ser	thr	N_HP	R_HP	leu	val	ile	met	ala	N_cys	R_cys	function
**eL38**	0	0	0	0,00	0,00	78	62,82	0	0	3	3,85	4	5,13	0	0	1	2	5	6,41	2	0	15	19,23	7	3	5	6,41	0	1	2	1	16	20,51	6	0	3	1	2	1	1,28	
**eL40**	1	1	1	0,00	0,07	128	58,59	6	4,69	2	1,56	4	3,12	0	1	1	2	11	8,59	5	5	12	9,38	7	4	17	13,28	2	6	2	7	23	17,97	9	4	7	1	1	0	0	
**uL10**	0	1	1	0,00	0,00	312	54,17	12	3,85	6	1,92	15	4,81	0	1	4	6	18	5,77	7	5	15	4,81	6	3	20	6,41	4	2	3	6	83	26,6	10	5	6	2	7	0	0	
**uL11**	0	1	1	0,00	0,00	165	75,15	13	7,88	10	6,06	3	1,82	1	1	0	0	13	7,88	5	3	21	12,73	12	4	18	10,91	2	2	5	4	45	27,27	9	5	6	2	7	1	0,61	
**eL29**	1	2	2	0,00	0,23	59	59,32	2	3,39	1	1,69	5	8,47	0	3	0	1	0	0	0	0	12	20,34	6	1	10	16,95	4	1	2	1	5	8,47	0	0	1	1	1	0	0	PTC
**eS19**	1	2	2	0,02	0,03	144	50	7	4,86	3	2,08	10	6,94	2	1	2	1	8	5,56	4	3	12	8,33	3	5	4	2,78	0	0	0	0	28	19,44	3	3	0	0	3	0	0	single_R2
**eS31**	1	2	2	0,15	0,03	152	84,87	10	6,58	4	2,63	12	7,89	0	3	5	3	14	9,21	6	6	31	20,39	22	8	21	13,82	2	6	3	10	33	21,71	12	6	7	2	3	4	2,63	tRNA-A
**uL22**	3	2	2	0,05	0,11	184	53,8	6	3,26	4	2,17	12	6,52	1	4	3	2	7	3,8	1	4	23	12,5	6	9	12	6,52	5	2	2	2	34	18,48	6	4	2	1	8	1	0,54	tunnel
**uS19**	2	2	2	0,00	0,05	142	78,17	8	5,63	6	4,23	12	8,45	0	3	3	5	9	6,34	0	5	29	20,42	13	10	12	8,45	2	0	4	2	35	24,65	10	3	3	4	2	0	0	single_R2
**RACK1**	0	3	3	0,07	0,03	319	57,05	14	4,39	4	1,25	28	8,78	10	6	1	3	17	5,33	13	0	15	4,7	4	6	38	11,91	2	1	18	2	63	19,75	17	9	3	0	4	3	0,94	single_R2
**eL32**	1	3	3	0,13	0,12	130	69,23	5	3,85	3	2,31	10	7,69	1	1	1	2	6	4,62	2	2	26	20	5	9	11	8,46	3	1	3	2	29	22,31	4	4	5	2	4	0	0	
**eS12**	1	2	3	0,06	0,01	143	47,55	6	4,2	0	0	4	2,8	1	0	1	0	8	5,59	1	2	9	6,29	5	2	2	1,4	0	0	1	0	35	24,48	8	4	0	0	4	4	2,8	
**eS24**	1	3	3	0,07	0,03	135	64,44	7	5,19	2	1,48	9	6,67	0	1	2	5	6	4,44	2	2	28	20,74	12	8	11	8,15	2	1	0	4	24	17,78	4	0	1	1	1	0	0	
**eS30**	1	3	3	0,09	0,05	63	71,43	5	7,94	1	1,59	3	4,76	0	1	1	1	1	1,59	0	0	16	25,4	6	5	8	12,7	2	2	1	1	11	17,46	1	3	0	0	3	0	0	tRNA-A
**uL1**	1	3	3	0,15	0,17	218	71,1	7	3,21	4	1,83	13	5,96	1	2	3	6	12	5,5	3	3	32	14,68	21	4	24	11,01	6	4	5	1	61	27,98	14	4	0	3	5	2	0,92	tRNA-E
**uL18**	2	3	3	0,07	0,25	297	55,89	14	4,71	4	1,35	24	8,08	0	2	9	5	18	6,06	5	4	33	11,11	10	13	18	6,06	4	3	0	6	55	18,52	9	4	2	2	8	0	0	double_R2-P2
**eL27**	1	3	4	0,17	0,09	136	64,71	6	4,41	2	1,47	13	9,56	1	1	1	4	5	3,68	1	0	26	19,12	8	4	8	5,88	1	0	0	1	28	20,59	2	4	0	3	1	0	0	
**eL30**	0	4	4	0,23	0,06	105	73,33	7	6,67	1	0,95	7	6,67	0	2	3	0	7	6,67	3	2	15	14,29	11	3	13	12,38	3	0	3	1	26	24,76	8	3	3	1	2	1	0,95	SB
**eL43**	2	2	4	0,09	0,07	92	76,09	8	8,7	0	0	7	7,61	1	1	3	1	3	3,26	0	1	19	20,65	10	6	9	9,78	0	1	2	3	20	21,74	2	2	1	1	4	4	4,35	
**eS25**	0	4	4	0,13	0,10	108	52,78	3	2,78	1	0,93	5	4,63	1	1	2	0	5	4,63	1	1	14	12,96	8	3	8	7,41	1	1	2	3	21	19,44	3	3	2	1	2	0	0	tRNA-E
**eS28**	2	3	4	0,01	0,05	67	73,13	4	5,97	1	1,49	1	1,49	0	0	0	1	6	8,96	1	4	11	16,42	1	9	7	10,45	1	1	1	2	19	28,36	4	6	1	1	2	0	0	single_R2
**eS7**	1	4	4	0,04	0,05	190	62,63	3	1,58	3	1,58	11	5,79	0	1	1	3	18	9,47	3	7	31	16,32	11	7	10	5,26	0	0	1	3	43	22,63	11	8	3	1	3	0	0	SB
**eS8**	2	2	4	0,00	0,02	202	62,38	12	5,94	2	0,99	15	7,43	2	2	5	4	9	4,46	3	5	36	17,82	11	14	20	9,9	5	2	5	4	32	15,84	8	5	3	1	4	0	0	
**uL23**	2	3	4	0,11	0,20	142	65,49	3	2,11	4	2,82	7	4,93	0	0	2	2	10	7,04	6	2	21	14,79	11	6	11	7,75	3	0	0	4	37	26,06	6	5	4	2	7	0	0	
**uL24**	2	4	4	0,04	0,44	127	76,38	5	3,94	3	2,36	7	5,51	0	1	2	1	9	7,09	4	1	31	24,41	12	13	13	10,24	1	0	6	0	29	22,83	4	7	2	2	1	0	0	single_T3
**uS14**	1	3	4	0,01	0,02	56	58,93	4	7,14	1	1,79	5	8,93	0	0	1	2	0	0	0	0	8	14,29	2	5	2	3,57	0	0	1	0	9	16,07	1	0	2	1	1	4	7,14	
**eL24**	1	3	5	0,01	0,08	155	42,58	3	1,94	2	1,29	9	5,81	1	0	1	1	4	2,58	1	0	24	15,48	8	6	6	3,87	0	0	1	1	17	10,97	1	0	2	0	2	1	0,65	SB
**eL34**	2	4	5	0,29	0,10	121	46,28	4	3,31	3	2,48	3	2,48	0	0	2	1	2	1,65	0	2	17	14,05	2	8	7	5,79	1	0	1	3	19	15,7	2	3	1	0	1	1	0,83	
**eL36**	1	4	5	0,12	0,38	110	49,09	5	4,55	1	0,91	3	2,73	0	0	1	0	6	5,45	0	4	19	17,27	7	7	3	2,73	0	0	0	2	17	15,45	1	2	0	0	1	0	0	
**eL37**	2	5	5	0,07	0,50	88	67,05	8	9,09	1	1,14	10	11,36	1	2	1	3	0	0	0	0	18	20,45	6	8	11	12,5	0	1	1	5	7	7,95	1	0	0	1	1	4	4,55	single_T3
**eL39**	1	5	5	0,30	0,33	51	68,63	0	0	2	3,92	5	9,8	2	1	1	1	0	0	0	0	14	27,45	5	7	7	13,73	3	1	0	2	7	13,73	2	0	1	0	1	0	0	single_T2
**eS10**	1	3	5	0,07	0,02	105	55,24	2	1,9	2	1,9	12	11,43	1	1	2	2	4	3,81	1	1	8	7,62	2	2	5	4,76	1	0	2	2	25	23,81	7	5	0	1	0	0	0	
**eS27**	1	4	5	0,02	0,05	82	78,05	4	4,88	3	3,66	6	7,32	0	2	0	3	4	4,88	2	2	10	12,2	4	3	13	15,85	1	1	3	5	18	21,95	6	4	1	2	2	6	7,32	
**eS6**	1	4	5	0,32	0,07	236	63,98	14	5,93	6	2,54	8	3,39	0	0	2	3	18	7,63	5	5	42	17,8	15	19	14	5,93	2	5	1	2	47	19,92	12	9	5	2	2	2	0,85	SB
**uL14**	2	3	5	0,04	0,15	137	81,02	11	8,03	5	3,65	7	5,11	1	0	2	1	9	6,57	4	3	21	15,33	11	8	13	9,49	6	1	2	1	43	31,39	7	8	4	2	11	2	1,46	PTC
**uL29**	1	4	5	0,06	0,39	120	60,83	0	0	2	1,67	4	3,33	0	0	1	0	5	4,17	1	1	27	22,5	10	10	7	5,83	0	0	0	3	28	23,33	10	3	2	1	3	0	0	
**uL30**	1	4	5	0,03	0,56	244	58,2	12	4,92	6	2,46	18	7,38	1	2	6	5	9	3,69	1	6	27	11,07	11	6	17	6,97	6	2	0	1	53	21,72	10	1	7	3	3	0	0	double_T1-P1
**uL33**	2	4	5	0,38	0,25	106	78,3	8	7,55	2	1,89	10	9,43	0	3	2	2	4	3,77	1	1	26	24,53	18	6	13	12,26	1	4	1	5	15	14,15	1	3	0	1	2	5	4,72	tRNA-E
**uL5**	2	5	5	0,26	0,15	174	76,44	15	8,62	3	1,72	17	9,77	1	2	5	6	16	9,2	4	7	24	13,79	8	9	17	9,77	5	2	3	3	41	23,56	9	7	7	1	4	0	0	tRNA-P
**uS10**	1	3	5	0,09	0,03	126	69,84	4	3,17	4	3,17	3	2,38	1	1	0	0	9	7,14	2	4	20	15,87	8	5	16	12,7	0	0	1	8	30	23,81	6	8	9	1	1	2	1,59	single_R2
**uS12**	2	5	5	0,62	0,08	145	79,31	13	8,97	5	3,45	9	6,21	1	1	1	4	10	6,9	4	5	28	19,31	16	8	12	8,28	4	2	2	0	36	24,83	8	10	4	0	6	2	1,38	mRNA
**uS13**	2	5	5	0,33	0,09	146	80,82	10	6,85	2	1,37	12	8,22	2	4	0	2	13	8,9	5	3	29	19,86	11	13	16	10,96	5	3	1	5	36	24,66	7	1	5	0	3	0	0	tRNA-A
**eL18**	2	4	6	0,03	0,50	186	63,44	9	4,84	6	3,23	9	4,84	0	1	2	4	5	2,69	4	1	30	16,13	5	12	14	7,53	2	1	5	4	45	24,19	11	3	1	1	5	0	0	
**eL19**	2	3	6	0,21	0,04	189	68,25	7	3,7	3	1,59	11	5,82	2	5	3	1	10	5,29	3	4	49	25,93	14	23	11	5,82	4	2	2	1	37	19,58	9	1	4	1	6	1	0,53	SB
**eL33**	4	4	6	0,14	0,13	110	56,36	7	6,36	2	1,82	10	9,09	1	1	5	2	2	1,82	0	0	14	12,73	1	6	8	7,27	1	0	1	0	19	17,27	4	3	1	0	2	0	0	
**eL6**	2	3	6	0,02	0,10	175	46,86	5	2,86	4	2,29	11	6,29	0	1	2	4	3	1,71	2	0	15	8,57	3	4	11	6,29	1	1	1	4	33	18,86	11	6	2	0	1	0	0	
**eL8**	3	6	6	0,80	0,33	262	57,25	7	2,67	11	4,2	12	4,58	2	1	3	3	12	4,58	5	4	35	13,36	13	9	15	5,73	2	3	0	3	57	21,76	13	8	4	2	7	1	0,38	SB
**eS1**	3	4	6	0,54	0,10	255	71,37	9	3,53	5	1,96	16	6,27	2	1	3	5	23	9,02	6	4	45	17,65	24	7	25	9,8	1	4	1	7	59	23,14	11	8	5	2	6	0	0	SB
**eS17**	1	4	6	0,30	0,04	137	63,5	4	2,92	2	1,46	6	4,38	0	1	2	1	12	8,76	2	6	22	16,06	8	7	11	8,03	2	1	3	4	30	21,9	5	5	4	2	2	0	0	
**uL13**	3	5	6	0,27	0,33	199	59,8	9	4,52	8	4,02	14	7,04	1	3	1	0	5	2,51	2	2	32	16,08	9	11	5	2,51	1	0	2	0	45	22,61	12	4	0	1	5	1	0,5	single_P2
**uL6**	0	5	6	0,21	0,36	191	59,69	9	4,71	3	1,57	12	6,28	1	2	3	4	10	5,24	4	4	18	9,42	6	7	12	6,28	4	1	4	2	50	26,18	3	9	5	3	4	0	0	PTC
**uS15**	3	5	6	0,22	0,06	151	80,79	8	5,3	7	4,64	11	7,28	2	3	5	1	10	6,62	4	3	28	18,54	14	11	17	11,26	1	2	6	3	41	27,15	12	5	7	2	5	0	0	SB
**uS9**	1	5	6	0,39	0,06	143	72,73	11	7,69	4	2,8	11	7,69	0	0	3	3	8	5,59	4	4	23	16,08	10	9	10	6,99	1	5	2	0	37	25,87	5	6	3	0	8	0	0	tRNA-P
**eL14**	2	4	7	0,09	0,29	138	42,75	3	2,17	1	0,72	6	4,35	0	0	0	1	6	4,35	3	1	12	8,7	1	4	3	2,17	0	0	0	1	28	20,29	3	3	1	0	0	0	0	
**eL15**	3	7	7	0,48	0,63	204	70,1	12	5,88	4	1,96	21	10,29	4	4	8	1	9	4,41	4	5	44	21,57	14	24	12	5,88	2	3	2	1	41	20,1	10	6	3	1	4	0	0	triple_R1-T1-P1
**eL21**	2	7	7	0,36	0,71	160	50	8	5	5	3,12	11	6,88	0	2	3	3	3	1,88	2	1	20	12,5	5	7	9	5,62	2	0	1	3	24	15	0	5	1	2	1	0	0	triple_R1-T1-P2
**eS4**	3	5	7	0,23	0,05	261	70,88	15	5,75	10	3,83	22	8,43	1	6	3	7	21	8,05	9	7	37	14,18	14	13	18	6,9	3	1	2	7	62	23,75	10	3	6	2	5	0	0	
**uL15**	1	5	7	0,25	0,37	149	68,46	17	11,41	6	4,03	17	11,41	1	9	2	1	4	2,68	2	1	24	16,11	11	7	7	4,7	2	0	2	0	27	18,12	5	4	1	2	4	0	0	
**uS2**	1	3	7	0,05	0,03	252	59,13	8	3,17	8	3,17	19	7,54	4	1	2	4	18	7,14	8	6	16	6,35	3	10	20	7,94	7	2	3	5	59	23,41	7	4	6	2	11	1	0,4	
**uS4**	3	5	7	0,28	0,06	197	70,56	9	4,57	5	2,54	14	7,11	1	4	2	4	16	8,12	5	7	37	18,78	11	20	12	6,09	2	3	4	3	46	23,35	16	8	5	0	5	0	0	single_R2
**uS7**	2	6	7	0,33	0,10	225	70,22	8	3,56	5	2,22	13	5,78	1	2	3	4	16	7,11	6	8	29	12,89	11	16	25	11,11	9	3	7	2	60	26,67	10	8	10	2	13	2	0,89	mRNA
**eS26**	3	4	8	0,02	0,05	119	59,66	2	1,68	4	3,36	7	5,88	0	2	3	1	4	3,36	2	0	23	19,33	7	12	6	5,04	3	0	3	0	21	17,65	1	3	2	1	3	4	3,36	
**uL16**	3	6	8	1,00	0,46	225	68	14	6,22	8	3,56	19	8,44	1	4	4	5	12	5,33	6	5	34	15,11	13	18	16	7,11	3	4	7	2	47	20,89	5	5	6	3	10	3	1,33	tRNA-A
**uL2**	3	5	8	0,48	0,29	252	75	31	12,3	8	3,17	22	8,73	1	6	3	3	12	4,76	3	5	37	14,68	11	19	17	6,75	4	3	4	5	60	23,81	6	7	4	2	12	2	0,79	PTC
**eL13**	2	7	9	0,26	0,66	199	50,25	6	3,02	5	2,51	10	5,03	2	1	2	4	4	2,01	2	2	30	15,08	5	13	11	5,53	3	2	1	3	34	17,09	7	4	2	1	4	0	0	double_R1-T1
**eL20**	4	7	9	0,58	0,72	172	54,07	4	2,33	5	2,91	17	9,88	2	2	6	5	5	2,91	1	3	24	13,95	5	9	9	5,23	2	1	2	0	29	16,86	2	2	3	3	3	0	0	triple_R1-T1-P3
**eS21**	3	4	10	0,04	0,05	87	52,87	4	4,6	1	1,15	3	3,45	0	1	1	0	6	6,9	4	0	5	5,75	2	3	7	8,05	2	1	2	0	19	21,84	2	2	1	1	3	1	1,15	
**uL3**	6	5	10	0,39	0,21	387	73,9	27	6,98	11	2,84	43	11,11	3	12	7	10	20	5,17	6	10	62	16,02	31	19	36	9,3	3	5	6	15	85	21,96	8	13	6	3	14	2	0,52	PTC
**uL4**	3	9	10	0,72	1,00	362	59,12	19	5,25	13	3,59	22	6,08	4	5	2	6	7	1,93	4	3	41	11,33	7	19	32	8,84	10	5	5	6	79	21,82	12	10	4	2	16	1	0,28	double_T2-P1
**uS8**	0	8	10	0,43	0,09	130	83,08	11	8,46	3	2,31	13	10	1	3	1	5	11	8,46	4	6	18	13,85	7	9	12	9,23	2	2	4	2	39	30	9	8	4	3	2	1	0,77	single_R2
**uS11**	3	6	11	0,30	0,10	137	90,51	15	10,95	5	3,65	8	5,84	0	3	1	3	13	9,49	7	4	21	15,33	6	12	20	14,6	1	2	5	8	41	29,93	6	7	5	3	14	1	0,73	tRNA-E
**uS17**	4	6	11	0,41	0,07	156	64,74	8	5,13	4	2,56	17	10,9	0	3	3	7	5	3,21	2	2	24	15,38	9	10	12	7,69	2	3	3	4	29	18,59	5	6	3	1	2	2	1,28	SB
**uS3**	2	7	11	0,40	0,05	240	71,25	17	7,08	6	2,5	12	5	0	1	4	5	22	9,17	5	12	32	13,33	12	16	19	7,92	1	4	5	5	61	25,42	13	13	6	5	10	2	0,83	mRNA
**uS5**	4	7	13	0,41	0,07	254	72,44	22	8,66	10	3,94	19	7,48	3	2	4	5	11	4,33	4	5	29	11,42	16	11	24	9,45	1	3	4	11	67	26,38	12	11	9	1	12	2	0,79	mRNA
**Av**		4,07	5,46	0,21	0,18	166,81	65,03	8,58	5,08	4,14	2,39	11,35	6,70	1,00	2,03	2,43	2,81	8,96	5,17	3,16	3,19	24,39	15,39	9,19	9,00	13,18	8,13	2,35	1,70	2,57	3,09	36,41	21,35	6,84	4,80	3,23	1,42	4,49	1,04	0,81	
**Sd**	2,83	4,95	9,19	0,29	0,05	124,45	6,80	15,56	6,12	4,95	0,06	10,61	1,66	2,12	1,41	2,12	2,12	4,24	1,47	1,41	3,54	9,90	5,52	6,36	5,66	13,44	2,15	0,71	1,41	1,41	7,07	36,06	4,15	4,24	7,78	4,24	0,00	7,07	0,71	0,35	

Table 7 (continued): eukaryotic network

	betweenness	degree	closeness
**N=2000**			
**ABE**	0,095	0,73	0,5615
**A**	0,0025	0,869	0,0395
**E**	0,001	0,561	0,0005
**B**	0,0045	0,955	0,0645
**N=4000**			
**ABE**	0,101	0,6355	0,56175
**A**	0,00975	0,6965	0,04425
**E**	0,00175	0,6515	0
**B**	0,01325	0,917	0,075
**N=10000**			
**ABE**	0,1885	0,5637	0,6104
**A**	0,0022	0,6327	0,0442
**E**	0,002	0,6122	0,0002
**B**	0,0075	0,9548	0,0385

Table 8

Table 9

	ABE	B	A	E
**Average distance**	**48.5**	**45.9**	**42.1**	**53.6**
**sigma**	**13.8**	**18.5**	**16.1**	**23.2**
**Largest distance**	**94.2**	**89.7**	**99.9**	**147.6**
**Protein-pairs > 70 Å**	**uS12 uS17**	**uS2 uS3**	**uL4 eL37**	**eS4 eS6**
	**uS13 tRNA-A**	**bL28 tRNA-E**	**eL15 uL33**	**eS6 eS24**
	**uS9 tRNA-P**	**bL27 tRNA-A**	**uL15 eL32**	**eL13 uL29**
		**uL4 bL20**	**eL19 uS17**	**uL4 uL30**
		**uL15 bL21**		**eS31 tRNA-A**
		**bL20 bL32**		**uL4 eL15**
				**uL16 uL18**
				**eS1 eS17**
				**eL8 eS1**
				**uL4 eL21**
				**eL8 eL27**
**Protein-pairs > 90 Å**				**eL8 uL23**
				**uL1 eL13**
				**eL19 uS17**
				**uL4 eL20**
				**eL19 eS7**

Organism	particle	Pdb_id	Method	Resolution (Å)
**Bacteria**				
**Actinobacteria**<br>*Mycobacterium smegmatis*	70S<br>mRNA<br>P/P tRNA-phe	5o61	Cryo-EM	3.31
**Proteobacteria**<br>*Escherichia coli*	70S	4ybb	X-ray	2.1
**Deinococcus-thermus**<br>*Thermus thermophilus*	70S<br>mRNA<br>A-, P-,E-tRNAs	4y4p	X-ray	2.5
**Archaea**				
**Euryarchaeota**<br>*Pyrococcus furiosus*	70S<br>P-, E-tRNAs	4v6u	Cryo-EM	6 .6
**Euryarchaeota**<br>*Pyrococcus abyssii*	50S<br>P-tRNA	5jbh	Cryo-EM	5.34
**Euryarchaeota**<br>*Haloarcula marismortui*	50S	1s72	X-ray	2.4
**Eukarya**				
**Chromoalveota**				
Plasmodium falciparum	80S<br>emetine	3j79	Cryo-EM	3.2
Toxoplasma gondii	80S	5xxb (large)<br>5xxu (small)	Cryo-EM	3.17
**Excavata**				
Leishmania donovani	80S	5t2a	Cryo-EM	2.9
Trichomonas vaginalis	80S	5xy3 (large)<br>5xyi (small)	Cryo-EM	3.35
**Opistokhont**				
Saccharomyces cerevisiae	80S	4v88	X-ray	3.0
Sus scrofa	80S-sec61<br>mRNA, A/P-tRNA, P/E-tRNA	3j7r	Cryo-EM	3.9
Homo sapiens	80S<br>init-tRNA	4ug0	Cryo-EM	3.6

Table 10

Supplementary document 1: Mathematical methods, Statistics of centralities

1 Centralities

In our study of the properties of the ribosomal network we have considered several centralities, let us recall their definition.

Notations: We will note a graph $G = (V, E)$ where V is its collection of vertices and E its collection of edges, in other words subsets of V of cardinal 2; in particular there are at most one edge between two vertices. For any finite W, we note $|W|$ its cardinal and in what follows $n = |V|$.

For $n \in \mathbb{N}$, we will denote the interval $[1, n] \subseteq \mathbb{N}$ as $[n]$.

Definition 1. (Degree centrality)
For any $v \in V$, the degree of v, noted as deg(v), is

$$deg(v) = |\{e \in E| \quad v \in e\}|$$

For any two $u, v \in V$, the length of a shortest path between u and v will be noted $d(u, v)$, a path being a sequence of edges and the length of a path being the size of the sequence. The number of shortest paths between $u, v \in V$ will be denoted $\sigma_{u,v}$.

Definition 2. (Closeness centrality)
The closeness centrality of a node u is given by,

$$C_c(u) = \frac{|V| - 1}{\sum\limits_{v:v \neq u} d(u, v)}$$

A vertex has a high closeness centrality when it is close to many vertices.

Remark 1. This definition is made according to the python package *Networkx.*

Definition 3. (Betweenness centrality)

For $v, v_1, u \in V$ the number of shortest path between v, v_1 that pass through u is denoted $\sigma_{v,v_1}(u)$.

The betweenness centrality of a vertex u is,

$$C_B(u) = \sum_{\substack{v,v_1: \\ v \neq u \neq v_1}} \frac{\sigma_{v,v_1}(u)}{\sigma_{v,v_1}}$$

A vertex has a high betweenness centrality when many shortest paths pass through it.

Definition 4. (Eigenvector centrality)

Let A be the adjacency matrix of G, and x the eigenvector (normalized for the l^2 norm) of its largest eigenvalue,

$$C_E(u) = x(u)$$

A vertex has high eigenvector centrality when it is specially involved in the heat propagation on the graph.

Remark 2. The adjacency matrix of a graph $G = (V, E)$ is defined as, for $u, v \in V$, $m_{u,v} = 1[\{u, v\} \in E]$ and there exist a unique normalized eigenvector for the maximal eigenvalue; all its components are strictly positive (Perron-Frobenius).

2 Sampling and coherence

In general when one wants to build a statistical test one needs to know the law of the test statistic; we show that in fact one can pass by this constraint but at the cost of defining a notion of coherence. Note that this argument is an improvement with respect to the litterature we know.

2.1 Sampling a probability law

Let Ω be a universe of events, i.e. a measurable space, P be a probability law on Ω. For a random variable $Y : \Omega \to \Omega^{'}$, i.e. a measurable function between two measurable spaces, we will note $P(Y \in A) = P(\{\omega : \quad Y(\omega) \in A\})$. For example $P(Id_\Omega \in \Omega) = 1$. We will note $cd(Y)$ the codomain of Y, i.e. $\Omega^{'}$.

Let us consider a random variable X from Ω to $[n]$ and $X_i, i \in [N]$ i.i.d random variables distributed as X, that we will denote as $X_{[N]}$. Then the empirical frequency count is

$$\forall k \in [n], \quad \widehat{XP}(k) := \frac{1}{N}\sum_{i\in[N]} 1[X_i = k]$$

where $1[X_i = k]$ is the indicatrix function of the set $\{X_i = k\}$. Then $\widehat{XP}$ is a random variable from $\Omega \times [n]$ to $[0,1] \subseteq \mathbb{R}$.

For $\omega \in \Omega$, $\widehat{XP}(\omega)$ is, most of the time, a good approximation of X_*P, i.e. the probability law of X; we will note X_*P as XP; this is a way to bypass the curse of dimensionality by going from exponential to polynomial complexity.

For any subset S of $cd(X)$,

$$\begin{aligned} P(||(\widehat{XP} - XP)_{|S}||_\infty \geq M) &\leq \sum_{k\in S} P(|\widehat{XP}(k) - XP(k)| \geq M) \\ &\leq \frac{1}{NM^2}\sum_{k\in S} V_P(1[X = k]) \end{aligned}$$

Where $V_P(1[X = k])$ is the variance of the random variable $1[X = k]$ which is given by $V(1[X = k]) = XP(k)(1 - XP(k))$. So the right hand side can be simplified into, $\frac{1}{NM^2}(\sum\limits_{k\in S} XP(k) - \sum\limits_{k\in S} XP(k)^2)$, which is always smaller than $\frac{1}{NM^2}(1 - \sum\limits_{k\in S} XP(k)^2) \leq \frac{1}{NM^2}$.

So to conclude,

$$P(||(\widehat{XP} - XP)_{|S}||_\infty \geq M) \leq \frac{1}{NM^2} \tag{1}$$

Therefore, if one wants to sample the marginal probability of X with an error of $\epsilon > 0$ at risk α, we need to have a sample of size, $N = \frac{1}{\alpha\epsilon^2}$. For $M = \epsilon = 0.1$, and $\alpha = 0.05$ one gets $N = 2000$.

For any $S \subseteq cd(X)$, let us denote $\widehat{XP}(X \in S) := \frac{1}{N}\sum\limits_{i\in[N]} 1[X_i \in S]$. Let $Y = 1[X \in S]$, $\widehat{YP}(1) = \frac{1}{N}\sum\limits_{i\in[N]} 1[X_i \in S] = \widehat{XP}(X \in S)$, we get from Equation.(1),

$$P(\widehat{XP}(X \in S) - P(X \in S)) \leq \frac{1}{NM^2} \tag{2}$$

2.2 Conditional test and coherence

We consider a variable T which will be the test statistic. We will assume that T goes from a measurable space Γ to $[n]$. Let us recall the usual decisions rule,

Definition 5. Let t be a realisation of T on the data; if under H_0, $P(T \geq t|H_0) \leq \alpha$, one rejects the null hypothesis, i.e. H_0.

Remark 3. $P(T \geq t|H_0)$ is the $p-$value.

Remark 4. A decision rule is in fact a way to force a binary logic on a non binary object. The usual decision rule 5 can be written as an indicatix function $1[P(T \geq .) \leq \alpha] : \Omega \to \{0,1\}$, where Ω is the universe and α is a given risk. So for any risk one has a truth value which corresponds to the rejection of the test and looking at the p-value is looking at all the $\omega \in \Omega$ such that H_0 can be rejected. In particular one concludes that for two tests, $1[P(T \geq .) \leq \alpha] \leq 1[P(T_1 \geq .) \leq \alpha]$, is the same than saying that the rejection for T implies the rejection for T_1.

$\Omega = [m]$, $\Gamma = \Omega$, hypothesis H_0 is that $X = Id_\Omega$ is distributed according to P. Let us choose a sample $x_i, i \in [N]$, in $\Omega^{[N]}$, for $t \in [n]$,

$$\widehat{TP}_{x_{[N]}}(t) = \frac{1}{N} \sum_{i \in [N]} 1[T(x_i) = t]$$

Definition 6. (Conditional test) Let t be a realisation of T on the data. The conditional test is defined as rejecting the null hypothesis if $\widehat{TP}_{x_{[N]}}(T \geq t) \leq \alpha$[1].

Note that

$$\widehat{TP}_{x_{[N]}}(T \geq t) := \frac{1}{N} \sum_{i \in [N]} 1[T(x_i) \geq t]$$

If $x_{[N]}$ and t are such that

$$|\widehat{TP}_{x_{[N]}}(T \geq t) - P(T \geq t)| > \epsilon \tag{3}$$

then $\widehat{TP}_{x_{[N]}}(T \geq t) \leq (\alpha - \epsilon)$ implies that $P(T \geq t) \leq \alpha$. This serves as motivation for the conditional test. And one can see that the testing problem is divided in two steps, the first one is to verifiy that $x_{[N]}$ verifies the inequality 3 and the second beeing the conditional test.

If we would like to refine the conditional test (6), we could ask that the rejection process is taken into consideration only when under H_0, $P(\widehat{TP}_{X_{[n]}}(Id_{cd(T)} \geq T) \leq \alpha) \leq \alpha_1$ for given α_1.

Under H_0 the law of $X_{[N]}$, i.i.d with respect to X, is $\underset{k \in [N]}{\otimes} P$. Therefore the law of $(X_{[N]}, X)$ is $\underset{[N+1]}{\otimes} P$ (as X independent of $X_{[N]}$ by hypothesis).

Definition 7. (Coherent conditional test) For a given couple α, α_1, one rejects the null hypothesis if $\widehat{TP}_{x_{[N]}}(T \geq t) \leq \alpha$ and $P(\widehat{TP}_{X_{[n]}}(Id_{cd(T)} \geq T) \leq \alpha) \leq \alpha_1$.

[1]The inequality is not strict as T takes values in a finite set: for example consider T a binary random variable and assume $T = 0$ almost surely, then astrict inequality would lead to an incoherent decision rule

As we said before it is difficult to compute TP and it was the whole point of the procedure to avoid computing it, however we can get a bound on the probability that appears in the second condition.

Theorem 1. *If* $\alpha_1 \geq \alpha + 3/(2^{2/3}N^{\frac{1}{3}})$ *then* $P(\widehat{TP}_{X_{[N]}}(Id_{cd(T)} \geq T) \leq \alpha) \leq \alpha_1$

Proof. For any $\epsilon > 0$

$$P(\widehat{TP}_{X_{[N]}}(Id_{cd(T)} \geq T) \leq \alpha) = \int \underset{[N+1]}{\otimes} P(x_{[N+1]})1[\widehat{TP}_{x_{[N]}}(Id_{cd(T)} \geq T(x_{N+1})) \leq \alpha]$$

$$= \int \underset{[N]}{\otimes} dP(x_{[N]}) \sum d[TP](t)1[\widehat{TP}_{x_{[N]}}(T \geq t) \leq \alpha)]1[|\widehat{TP}_{x_{[N]}}(T \geq t) - P(T \geq t)| \leq \epsilon]$$

$$+ \int \underset{[N]}{\otimes} dP(x_{[N]}) \sum d[TP](t)1[\widehat{TP}_{x_{[N]}}(T \geq t) \leq \alpha)]1[|\widehat{TP}_{x_{[N]}}(T \geq t) - P(T \geq t)| > \epsilon]$$

If $\widehat{TP}_{X_{[N]}}[(T \geq t) \leq \alpha]$ and $|\widehat{TP}_{X_{[N]}}(T \geq t) - P(T \geq t)| < \epsilon$ then $P(T \geq t) \leq \alpha + \epsilon$ and the first part of the sum is lower than $\alpha + \epsilon$. The second part of the sum is lower than $\int \underset{[n]}{\otimes} dP(x_{[N]}) \sum d[TP](t)1[|\widehat{TP}_{x_{[N]}}(T \geq t) - P(T \geq t)| > \epsilon] \leq 1/N\epsilon^2$ (by Equation.(2). So one gets that for any $\epsilon > 0$,

$$P(\widehat{TP}_{X_{[N]}}(T \geq t) \leq \alpha) \leq \alpha + \epsilon + 1/(N\epsilon^2)$$

One would like to find at least one $\epsilon > 0$ such that $\alpha + \epsilon + 1/(N\epsilon^2) \leq \alpha_1$.

This inequality has solutions if and only if $\alpha_1 \geq \alpha + 3/(2^{2/3}N^{\frac{1}{3}})$.

□

Remark 5. In the usual test, the consistency of the test is justified by the fact that, under H_0, when N goes to ∞, T is almost surely constant.

In the case of the conditional test and coherent conditional test, there are two possible forms of consistency. When $N \to \infty$, $\widehat{TP}_{X_{[N]}}(T \geq t)$ is almost surely $P(T \geq t)$ under H_0 and this form is satisfied by our method. The second one is to insure that the test converges when both N and n tend to infinity. However n is dependent on the experimental data and not of the method; then this form of consistency makes no sense for the ribosomal networks.

2.3 Application to centralities

Our data is the graph of connections inside of a ribosome $g = (V, E)$ and $n = |V|$. We will start with the hypothesis H_0 that the ribosomal networks are random in each kingdom and refute this hypothesis thanks to a test. The model for randomness that we choose, in other word the hypothesis H_0, is the Erdös-Rényi random graph model $G(n, p)$, where n is the number of nodes of the ribosomal network and p is the proportion of edges in the observed graph with respect to

the complete graph, i.e. $p = \frac{2|E|}{n(n-1)}$.

Most of the centralities, that we will generically denote as C, take values in $\mathbb{R}$ and we want to give ourselves bins on the values of these centralities; in other words we consider a collection of sets $R_k, k \in [n]$ and consider the random variable for all $x \in V$, $X(x) = \sum k1[C(G)(x) \in R_k]$. In practive we choose as R_k a disjoint union of sets on the range of values of the centralities of the graph g we observe, the one on wich we want to do a test. X is a finite random variable (as $cd(X)$ is finite).

We now consider the histogram of X, in other words:

$$Y(k) = \frac{1}{n} \sum_{x \in V} 1[C(G)(x) \in \underset{j \leq k}{\cup} R_j]$$

Y is a finite random variable. The test statistic we would like to consider is $T = ||Y - E[Y]||_\infty$, where $E[Y]$ is the expectation of Y under H_0. However we don't have access to $E[Y]$, therefore we sample $E[Y]$ and we get $\widehat{E}[Y]$. The test statistic we consider is $T = ||Y - \widehat{E}[Y]||_\infty$, which is a finite random variable, i.e. $cd(T)$ is finite; the conditional p-values are given in the table of p-values.

In our case $N = 2000$ and so α_1 has to be greater than $\alpha + 0.15$ by Theorem.(1), for $N = 4000$, α_1 must be greater or equal than $\alpha + 0.119$. We then conclude that H_0 is rejected.
